# Supplementary material for: Comprehensive Identification of Ginsenosides in the Roots and Rhizomes of Panax ginseng Based on Their Molecular Features-Oriented Precursor Ions Selection and Targeted MS/MS Analysis
Source: Molecules. 2023 Jan 17;28(3):941. doi: 10.3390/molecules28030941 (PMC9919916; doi:10.3390/molecules28030941)
Supplement: Supplementary file 1 [file molecules-28-00941-s001.zip › molecules-2176682-supplementary.pdf]

Hong-Ping Wang [1,*], Zi-Jian Wang [1], Jing Du [1], Zhao-Zhou Lin [2], Chen Zhao [1], Run Zhang [3], Qiong Yin [1], Chun-Lan Fan [2], Ping Peng [1] and Zhi-Bin Wang [1]

[1] Scientific Research Institute of Beijing Tongrentang Co., Ltd., Beijing 100011, China
[2] Beijing Tongrentang Technology Development Co., Ltd., Beijing 100079, China
[3] Beijing Zhongyan Tongrentang Pharmaceutical R & D Co., Ltd., Beijing 100000, China
* Correspondence: sungirl9626@163.com; Tel./Fax: +86-10-87632655

Supplementary Information

Table S1. The isolated ginsenosides from the roots and rhizomes of *panax ginseng*

No.	Ginsenosides	R_1	R_2	C_{20}	Molecular formula	Molecular weight	The calculated exact mass of $[M-H]^-$ ion

The protopanaxadiol ginsenosides

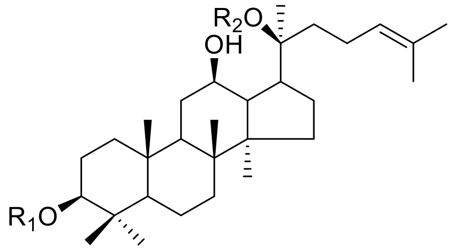

20(*S*)-protopanaxadiol

1	ginsenoside Ra_1	-glc(2-1)glc	-glc(6-1)ara(p)(4-1) xyl	*S*	$C_{58}H_{98}O_{26}$	1210	1209.6268
2	ginsenoside Ra_2	-glc(2-1)glc	-glc(6-1)ara(f)(2-1) xyl	*S*	$C_{58}H_{98}O_{26}$	1210	1209.6268
3	ginsenoside Ra_3	-glc(2-1)glc	-glc(6-1)glc(3-1) xyl	*S*	$C_{59}H_{100}O_{27}$	1240	1239.6374
4	ginsenoside Ra_4	-glc(2-1)glc(6)(*E*)-but-2-enoyl	-glc(6-1)ara(p)(4-1) xyl	*S*	$C_{62}H_{102}O_{27}$	1278	1277.6530
5	ginsenoside Ra_5	-glc(2-1)glc(6)-ace	-glc(6-1)ara(p)(4-1	*S*	$C_{60}H_{100}O_{27}$	1252	1251.6374

		tyl	) xyl				
6	ginsenoside Ra_6	-glc(2-1)glc(6)(*E*)-but-2-enoyl	-glc(6-1)glc	*S*	$C_{58}H_{96}O_{24}$	1176	1175.6213
7	ginsenoside Ra_7	-glc(2-1)glc(6)(*E*)-but-2-enoyl	-glc(6-1)ara(p)	*S*	$C_{57}H_{94}O_{23}$	1146	1145.6108
8	ginsenoside Ra_8	-glc(2-1)glc(4)(*E*)-but-2-enoyl	-glc(6-1)ara(f)	*S*	$C_{57}H_{94}O_{23}$	1146	1145.6108
9	ginsenoside Ra_9	-glc(2-1)glc(6)(*E*)-but-2-enoyl	-glc(6-1)ara(f)	*S*	$C_{57}H_{94}O_{23}$	1146	1145.6108
10	ginsenoside Rb_1	-glc(2-1)glc	-glc(6-1)glc	*S*	$C_{54}H_{92}O_{23}$	1108	1107.5951
11	ginsenoside Rb_2	-glc(2-1)glc	-glc(6-1)ara(p)	*S*	$C_{53}H_{90}O_{22}$	1078	1077.5845
12	ginsenoside Rb_3	-glc(2-1)glc	-glc(6-1)xyl	*S*	$C_{53}H_{90}O_{22}$	1078	1077.5845
13	ginsenoside Rc	-glc(2-1)glc	-glc(6-1)ara(f)	*S*	$C_{53}H_{90}O_{22}$	1078	1077.5845
14	ginsenoside Rd	-glc(2-1)glc	-glc	*S*	$C_{48}H_{82}O_{18}$	946	945.5423
15	ginsenoside Rg_3	-glc(2-1)glc	-H	*S*	$C_{42}H_{72}O_{13}$	784	783.4895
16	ginsenoside Rh_2	-glc	-H	*S*	$C_{36}H_{62}O_8$	622	621.4366
17	malonyl-ginsenoside Rb_1	-glc(2-1)glc(6)ma	-glc(6-1)glc	*S*	$C_{57}H_{94}O_{26}$	1194	1193.5955
18	malonyl-ginsenoside Rb_2	-glc(2-1)glc(6)ma	-glc(6-1)ara(p)	*S*	$C_{56}H_{92}O_{25}$	1164	1163.5849
19	malonyl-ginsenoside Rc	-glc(2-1)glc(6)ma	-glc(6-1)ara(f)	*S*	$C_{56}H_{92}O_{25}$	1164	1163.5849
20	malonyl-ginsenoside Rd	-glc(2-1)glc(6)ma	-glc	*S*	$C_{51}H_{84}O_{21}$	1032	1031.5427
21	malonyl-ginsenoside Ra_3	-glc(2-1)glc(6)ma	-glc(6-1)glc(3-1) xyl	*S*	$C_{62}H_{102}O_{30}$	1326	1325.6378
22	malonyl-notoginsenoside R_4	-glc(2-1)glc(6)ma	-glc(6-1)glc(6-1) xyl	*S*	$C_{62}H_{102}O_{30}$	1326	1325.6378
23	notoginsenoside R_4	-glc(2-1)glc	-glc(6-1)glc(6-1) xyl	*S*	$C_{59}H_{100}O_{27}$	1240	1239.6374
24	ginsenoside Rs_1	-glc(2-1)glc(6)Ac	-glc(6-1)ara(p)	*S*	$C_{55}H_{92}O_{23}$	1120	1119.5951

25	ginsenoside Rs$_2$	-glc(2-1)glc(6)Ac	-glc(6-1)ara(f)	*S*	$C_{55}H_{92}O_{23}$	1120	1119.5951
26	gypenoside XVII	-glc	-glc(6-1)glc	*S*	$C_{48}H_{82}O_{18}$	946	945.5423
27	pseudoginsenoside RC$_1$	-glc(2-1)glc(6)Ac	-glc	*S*	$C_{50}H_{84}O_{19}$	988	987.5529
28	quinquenoside R$_1$	-glc(2-1)glc(6)Ac	-glc(6-1)glc	*S*	$C_{56}H_{94}O_{24}$	1150	1149.6057
29	Vina-ginsenoside R$_{16}$	-glc(2-1)xyl	-glc	*S*	$C_{47}H_{80}O_{17}$	916	915.5317

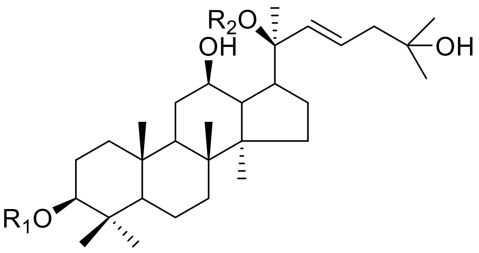

20(*S*)-protopanaxadiol

30	koryoginsenoside R$_2$	-glc(2-1)glc	-glc(6-1)glc	*S*	$C_{54}H_{92}O_{24}$	1124	1123.5900

The protopanaxatriol ginsenosides

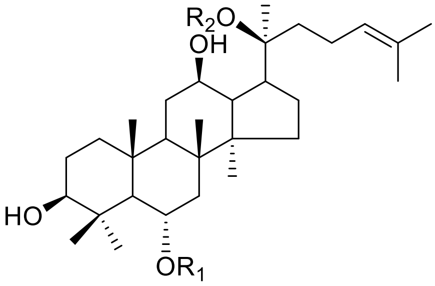

20(*S*)-protopanaxatriol

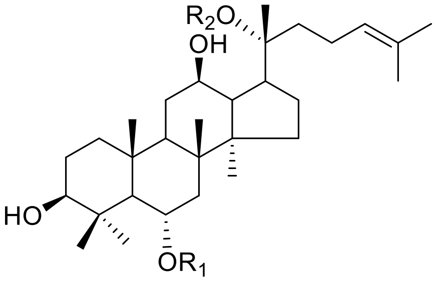

20(*R*)-protopanaxatriol

31	ginsenoside Re	-glc(2-1)rha	-glc	*S*	$C_{48}H_{82}O_{18}$	946	945.5423
32	ginsenoside Re$_1$	-glc	-glc(3-1)glc	*S*	$C_{48}H_{82}O_{19}$	962	961.5372
33	ginsenoside Re$_2$	-glc(3-1)glc	-glc	*S*	$C_{48}H_{82}O_{19}$	962	961.5372
34	ginsenoside Re$_3$	-glc	-glc(4-1)glc	*S*	$C_{48}H_{82}O_{19}$	962	961.5372
35	ginsenoside Re$_4$	-glc	-glc(6-1)ara(f)	*S*	$C_{47}H_{80}O_{18}$	932	931.5266
36	ginsenoside Re$_6$	-glc	-glc(6)(*E*)-but-2-enoyl	*S*	$C_{46}H_{76}O_{15}$	868	867.5106

37	ginsenoside Rf	-glc(2-1)glc	H	*S*	$C_{42}H_{72}O_{14}$	800	799.4844
38	ginsenoside Rg_1	-glc	-glc	*S*	$C_{42}H_{72}O_{14}$	800	799.4844
39	ginsenoside Rg_2	-glc(2-1)rha	-H	*S*	$C_{42}H_{72}O_{13}$	784	783.4895
40	20-(*R*)-ginsenoside Rg_2	-glc(2-1)rha	-H	*R*	$C_{42}H_{72}O_{13}$	784	783.4895
41	20-gluco-ginsenoside Rf	-glc(2-1)glc	-glc	*S*	$C_{48}H_{82}O_{19}$	962	961.5372
42	ginsenoside Rh_1	-glc	-H	*S*	$C_{36}H_{62}O_9$	638	637.4316
43	notoginsenoside R_1	-glc(2-1)xyl	-glc	*S*	$C_{47}H_{80}O_{18}$	932	931.5266
44	notoginsenoside R_2	-glc(2-1)xyl	-H	*S*	$C_{41}H_{70}O_{13}$	770	769.4738
45	koryoginsenoside R_1	-glc(6)(*E*)-but-2-enoyl	-glc	*S*	$C_{46}H_{76}O_{15}$	868	867.5106
46	yesanchinoside D	-glc(6)Ac	-glc	*S*	$C_{44}H_{74}O_{15}$	842	841.4949
47	notoginsenoside N	-glc(4-1)glc	-glc	*S*	$C_{48}H_{82}O_{19}$	962	961.5372

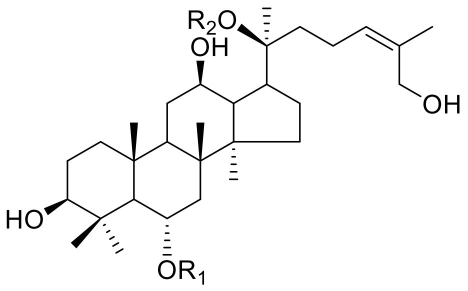

Ginsenoside Re_5

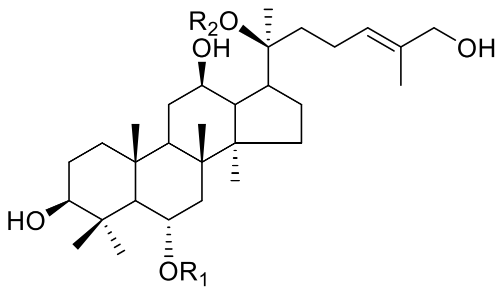

Ginsenjilinol

48	ginsenoside Re_5	-glc(2-1)glc	-H	*S*	$C_{42}H_{72}O_{15}$	816	815.4793
49	ginsenjilinol	-glc(2-1)glc	-H	*S*	$C_{42}H_{72}O_{15}$	816	815.4793

The protopanaxadiol ginsenosides

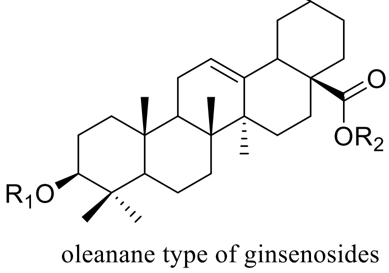

oleanane type of ginsenosides

50	ginsenoside Ro	-glu A(2-1)glc	-glc	-	$C_{48}H_{76}O_{19}$	956	955.4903
51	polyacetyleneginsenoside-Ro	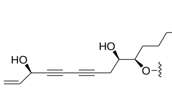	-H	-	$C_{65}H_{100}O_{21}$	1216	1215.6679

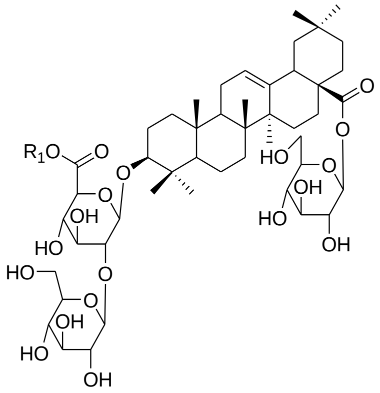

52	ginsenoside Ro methyl ester	Me	-H	-	$C_{49}H_{78}O_{19}$	970	969.5059

ara(p): α-L-arabinopyranosyl; ara(f): α-L-arabinofuranosyl; glc: β-D-glucopyranoside; xyl: β-D-xylopyranoside.

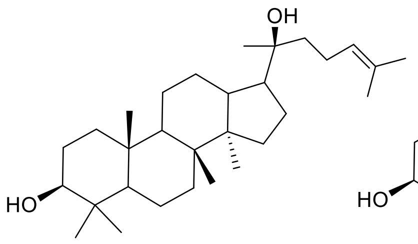

the aglycone of vinaginsenoside R_3

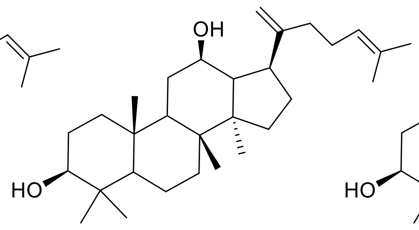

the aglycone of ginsenoside Rk_1

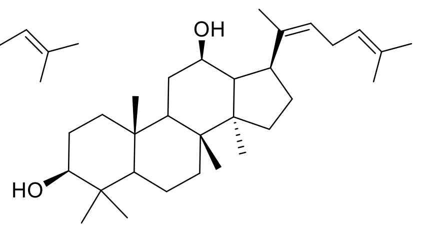

the aglycone of ginsenoside Rg_5

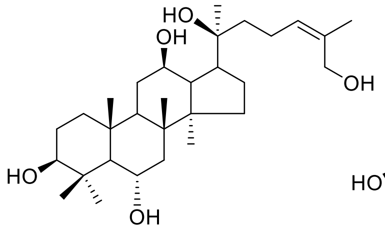

the aglycone of ginsenoside Re_5

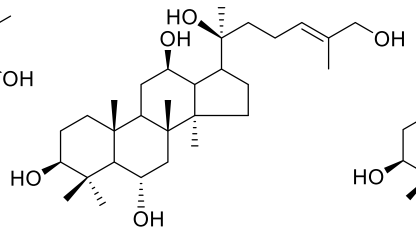

the aglycone of ginsenjilinol

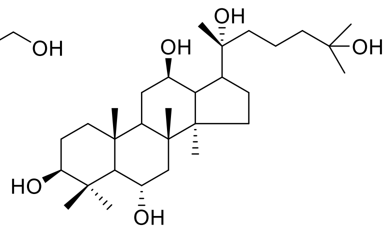

the aglycone of ginsenoside Rf_2

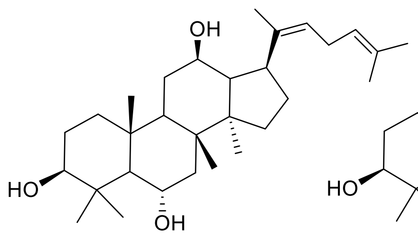

the aglycone of ginsenoside F4

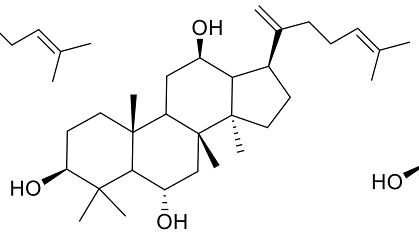

the aglycone of ginsenoside Rg_6

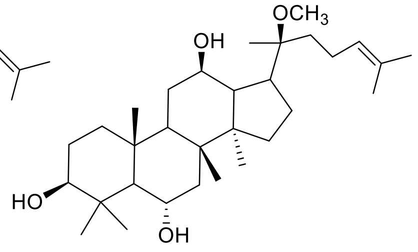

the aglycone of ginsenoside Rh_5

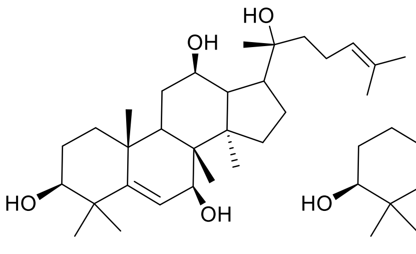

the aglycone of ginsenoside Rh_7

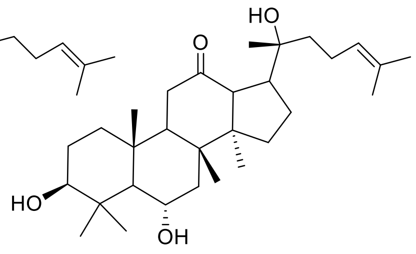

the aglycone of ginsenoside Rh_8

Figure S1. The aglycones of part of ginsenosides identified in the extract of *panax ginseng*.

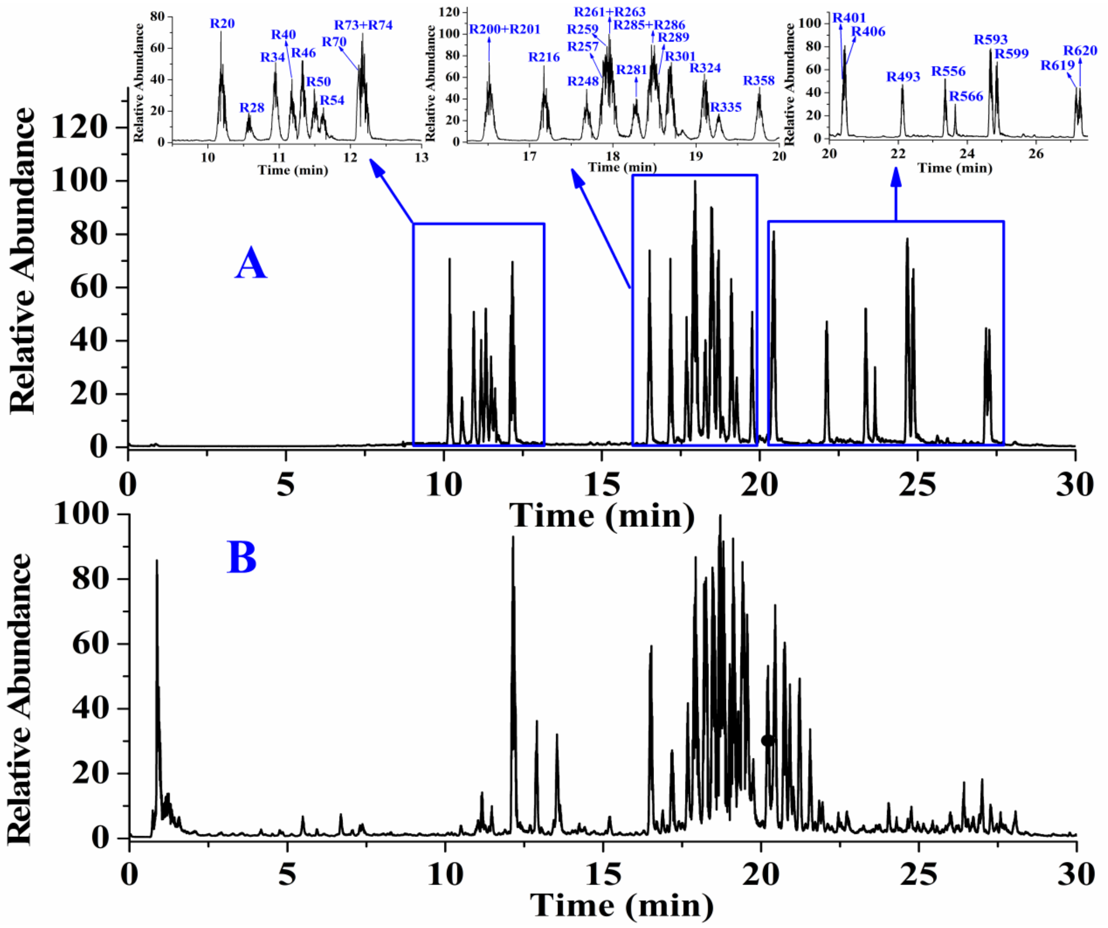

Figure S2. Total ion chromatograms of 35 reference standards (**A**) and the extract of RRPG (**B**).

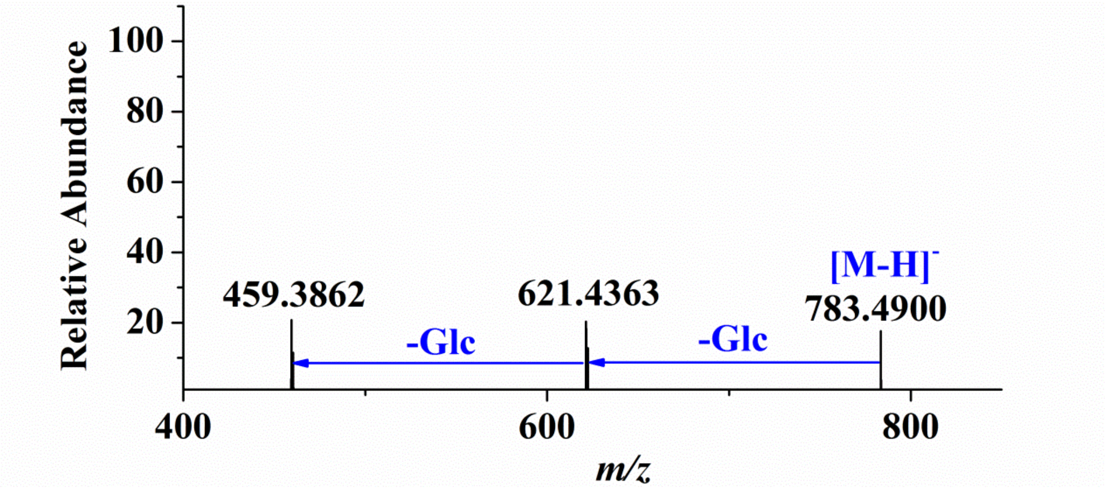

Figure S3. The MS/MS spectrum of the reference standard ginsenoside Rg_3.

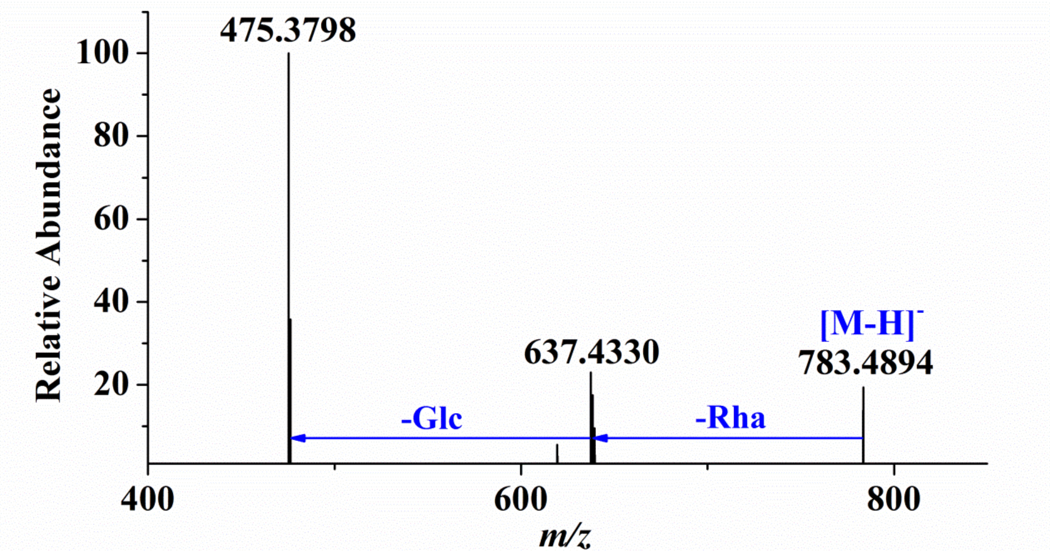

Figure S4. The MS/MS spectrum of the reference standard ginsenoside Rg_2.

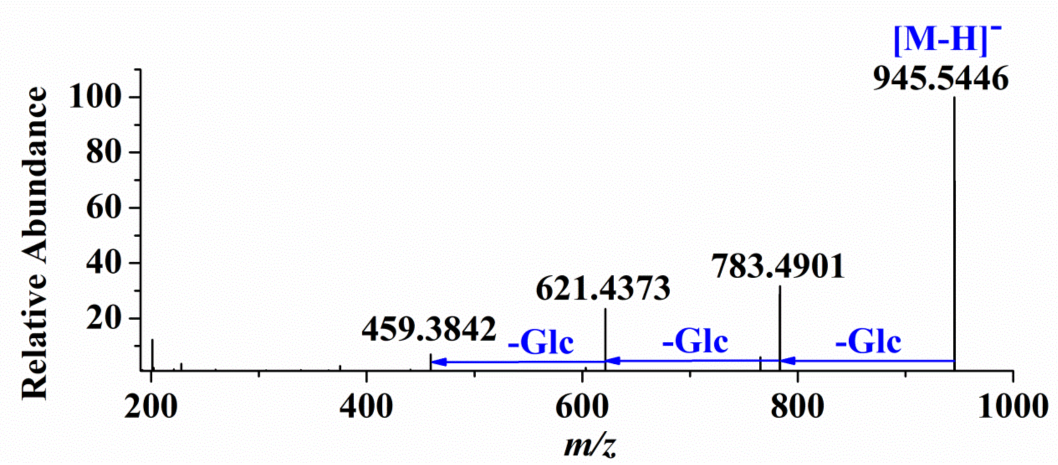

Figure S5. The MS/MS spectrum of the reference standard ginsenoside Rd.

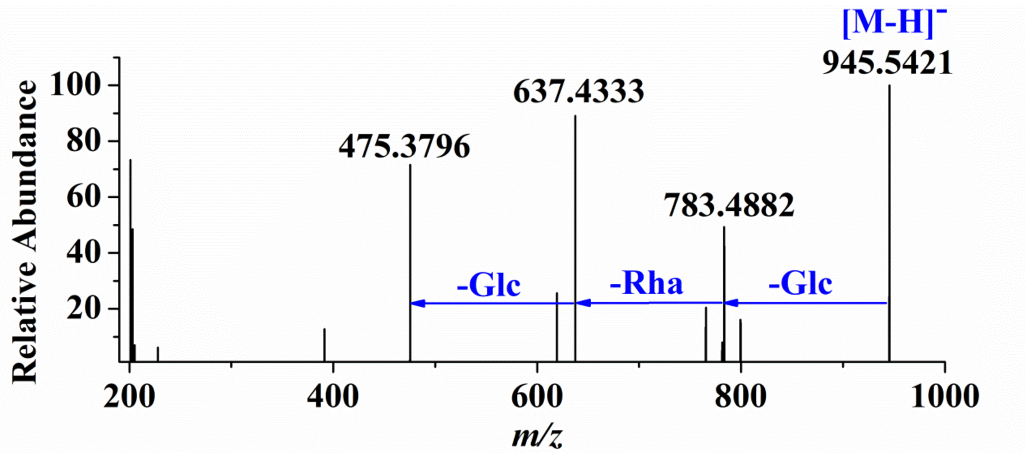

Figure S6. The MS/MS spectrum of the reference standard ginsenoside Re.

Table S2. The identified ginsenosides from the extract solution of RRPG.

No.	t_R (min)	Molecular formula	Measured value (m/z)	Diff (ppm)	Product ions	Compound name
R1	6.99	$C_{48}H_{84}O_{20}$	979.5486	0.82	817.4918[M-H-Glc]$^-$, 799.4863[M-H-Glc-H_2O]$^-$, 671.4409[M-H-Glc-Rha]$^-$, 653.4276[M-H-Glc-Rha-H_2O]$^-$, 635.4174[M-H-Glc-Rha-2H_2O]$^-$, 509.3840[M-H-2Glc-Rha]$^-$, 491.3755[M-H-2Glc-Rha-H_2O]$^-$,	Oxidated+hydrated protopanaxatriol+2 Glc+Rha
R2	7.20	$C_{48}H_{84}O_{20}$	1025.5530 [b]	-0.20	817.4996[M-H-Glc]$^-$, 799.4866[M-H-Glc-H_2O]$^-$, 671.4429[M-H-Glc-Rha]$^-$, 653.4279[M-H-Glc-Rha-H_2O]$^-$, 635.4166[M-H-Glc-Rha-2H_2O]$^-$, 509.3836[M-H-2Glc-Rha]$^-$, 491.3745[M-H-2Glc-Rha-H_2O]$^-$	Oxidated+hydrated protopanaxatriol+2 Glc+Rha
R3	8.04	$C_{42}H_{72}O_{15}$	861.4847 [b]	-0.12	653.4335[M-H-2Glc]$^-$, 491.3773[M-H-2Glc]$^-$	Ginsenoside Re_5 isomer/Ginsenjilinol isomer
R4	8.12	$C_{48}H_{82}O_{20}$	1023.5377 [b]	0.10	977.5248[M-H]$^-$, 815.4767[M-H-Glc]$^-$, 797.4711[M-H-Glc-H_2O]$^-$, 653.4276[M-H-2Glc]$^-$, 635.4193[M-H-2Glc-H_2O]$^-$, 491.3737[M-H-3Glc]$^-$	Oxidated-protopan axatriol+3Glc
R5	8.24	$C_{42}H_{74}O_{15}$	863.5000 [b]	-0.46	655.4158[M-H-Glc]$^-$, 493.3926[M-H-2Glc]$^-$	Double bond hydrated-protopana

						xatriol+2Glc
R6	8.28	$C_{48}H_{82}O_{19}$	961.5383	1.14	799.4803[M-H-Glc]$^-$, 781.4755[M-H-Glc-H_2O]$^-$, 635.4137[M-H-Glc-H_2O-Rha]$^-$, 491.3736[M-H-2Glc-Rha]$^-$	Oxidated-protopanaxatriol+2Glc+Rha
R7	8.44	$C_{48}H_{82}O_{19}$	961.5387	1.56	799.4922[M-H-Glc]$^-$, 781.4753[M-H-Glc-H_2O]$^-$, 635.4191[M-H-Glc-H_2O-Rha]$^-$, 491.3735[M-H-2Glc-Rha]$^-$	Oxidated-protopanaxatriol+2Glc+Rha
R8	8.47	$C_{42}H_{72}O_{15}$	861.4852[b]	0.46	653.4305[M-H-2Glc]$^-$, 491.3784[M-H-2Glc]$^-$	Ginsenoside Re$_5$ isomer/Ginsenjilinol isomer
R9	8.71	$C_{42}H_{72}O_{15}$	861.4856[b]	0.93	653.4321[M-H-2Glc]$^-$, 491.3744[M-H-2Glc]$^-$	Ginsenoside Re$_5$ isomer/Ginsenjilinol isomer
R10	8.80	$C_{42}H_{70}O_{15}$	859.4696[b]	0.58	651.4141[M-H-Glc]$^-$, 489.3577[M-H-2Glc]$^-$	Methyl etherified-protopanaxatriol+2Glc
R11	9.29	$C_{42}H_{72}O_{15}$	861.4854[b]	0.70	653.4230[M-H-2Glc]$^-$, 491.3721[M-H-2Glc]$^-$	Ginsenoside Re$_5$ isomer/Ginsenjilinol isomer
R12	9.34	$C_{42}H_{72}O_{16}$	877.4809[b]	1.37	831.4691[M-H]$^-$, 669.4186[M-H-Glc]$^-$, 507.3710[M-H-2Glc]$^-$	Dioxidated -protopanaxatriol+2Glc
R13	9.38	$C_{42}H_{70}O_{15}$	859.4695[b]	0.47	813.4571[M-H]$^-$, 651.4139[M-H-Glc]$^-$, 489.3561[M-H-2Glc]$^-$	Methyl etherified-protopanaxatriol+2Glc
R14	9.39	$C_{48}H_{82}O_{19}$	961.5380	0.83	781.4695[M-H-Glc-H_2O]$^-$, 635.4142[M-H-Glc-H_2O-Rha]$^-$, 491.3722[M-H-2Glc-Rha]$^-$	Oxidated-protopanaxatriol+2Glc+Rha

R15	9.44	$C_{42}H_{72}O_{15}$	815.4800	0.86	653.4324[M-H-2Glc]⁻， 491.3729[M-H-2Glc]⁻	Ginsenoside Re_5 isomer/Ginsenjilinol isomer
R16	9.70	$C_{42}H_{74}O_{15}$	863.5018 [b]	1.62	817.4910[M-H]⁻, 671.4387[M-H-Rha]⁻, 653.4279[M-H-Rha-H_2O]⁻, 509.3851[M-H-Rha-Glc]⁻	Oxidated+hydrated protopanaxatriol+Glc+Rha
R17	9.75	$C_{42}H_{72}O_{15}$	815.4793	0.00	653.4119[M-H-2Glc]⁻， 491.3717[M-H-2Glc]⁻	Ginsenoside Re_5 isomer/Ginsenjilinol isomer
R18	10.07	$C_{42}H_{72}O_{15}$	815.4795	0.25	653.4203[M-H-2Glc]⁻， 491.3749[M-H-2Glc]⁻	Ginsenoside Re_5 isomer/Ginsenjilinol isomer
R19	10.11	$C_{53}H_{90}O_{23}$	1093.5796	0.09	961.5309[M-H-Ara]⁻, 799.4929[M-H-Ara-Glc]⁻, 637.4329[M-H-Ara-2Glc]⁻, 475.3816[M-H-Ara-3Glc]⁻	Floralginsenoside P/isomer
R20	10.20	$C_{42}H_{72}O_{15}$	815.4794	0.12	653.4243[M-H-2lc]⁻， 491.3737[M-H-2Glc]⁻	Ginsenoside Re_5 [a]
R21	10.27	$C_{47}H_{80}O_{18}$	931.5269	0.32	491.3748[M-H-Ara/Xyl-Rha-Glc]⁻	Oxidated-protopanaxatriol+Ara/Xyl+Rha+Glc
R22	10.31	$C_{54}H_{92}O_{23}$	1107.5944	-0.63	945.5400[M-H-Glc]⁻, 783.4881[M-H-2Glc]⁻, 637.4347[M-H-2Glc-Rha]⁻, 475.3798[M-H-3Glc-Rha]⁻	Protopanaxatriol+3Glc+Rha
R23	10.34	$C_{47}H_{80}O_{18}$	931.5268	0.21	653.4296[M-H-Ara/Xyl-Rha]⁻, 491.3808[M-H-Ara/Xyl-Rha-Glc]⁻	Oxidated-protopanaxatriol+Ara/Xyl+Rha+Glc
R24	10.41	$C_{42}H_{72}O_{15}$	815.4804	1.35	653.4332[M-H-2Glc]⁻， 491.3715[M-H-2Glc]⁻	Ginsenoside Re_5 isomer/Ginsenjilinol isomer
R25	10.42	$C_{53}H_{90}O_{23}$	1093.5800	0.46	799.4976[M-H-Ara-Glc]⁻, 637.4310[M-H-Ara-2Glc]⁻,	Floralginsenoside

					475.3813[M-H-Ara-3Glc]-	P/isomer
R26	10.50	$C_{58}H_{98}O_{26}$	1209.6240	-2.30	1077.5824[M-H-Xyl/Ara]-, 945.5352[M-H-Xyl-Ara]-, 783.4899[M-H-Xyl-Ara-Glc]-, 637.4327[M-H-Xyl-Ara-Glc-Rha]-, 475.3799[M-H-Xyl-Ara-2Glc-Rha]-	Protopanaxatriol+2 Glc+Rha+Xyl+Ara
R27	10.53	$C_{54}H_{92}O_{23}$	1107.5938	-1.17	945.5454[M-H-Glc]-, 783.4970[M-H-2Glc]-, 637.4352[M-H-2Glc-Rha]-, 475.3832[M-H-3Glc-Rha]-	Protopanaxatriol+3 Glc+Rha
R28	10.57	$C_{48}H_{82}O_{19}$	961.5372	0.00	799.4865[M-H-Glc]-, 637.4351[M-H-2Glc]-, 475.3785[M-H-3Glc]-,	Ginsenoside Re_3 [a]
R29	10.63	$C_{48}H_{82}O_{20}$	1023.5371 [b]	-0.49	815.4866[M-H-Glc]-, 797.4678[M-H-Glc-H_2O]-, 653.4309[M-H-2Glc]-, 635.4155[M-H-2Glc-H_2O]-, 491.3808[M-H-3Glc]-, 473.3588[M-H-3Glc-H_2O]-	Oxidated-protopan axatriol+3Glc
R30	10.84	$C_{45}H_{74}O_{18}$	901.4805	0.89	815.4798[M-H-Malonyl]-, 653.4215[M-H-Malonyl-Glc]-, 491.3746[M-H-Malonyl-2Glc]-,	Oxidated-protopan axatriol+Glc+Malon yl Glc
R31	10.86	$C_{42}H_{70}O_{15}$	859.4698 [b]	0.81	813.4571[M-H]-, 651.4103[M-H-Glc]-, 489.3588[M-H-2Glc]-	Methyl etherified-protopan axatriol+2Glc
R32	10.88	$C_{51}H_{84}O_{22}$	1047.5388	1.15	961.5326[M-H-Malonyl]-, 799.4797[M-H-Malonyl-Glc]-, 637.4406[M-H-Malonyl-2Glc]-, 475.3806[M-H-Malonyl-3Glc]-	Malonyl-ginsenosid e Malonyl-ginsenosid e Re_3

R33	10.91	$C_{42}H_{72}O_{14}$	799.4855	1.38	653.4247[M-H-Rha]$^-$, 491.3707[M-H-Rha-Glc]$^-$	Oxidated-protopanaxatriol+Glc+Rha
R34	10.96	$C_{47}H_{80}O_{18}$	931.5270	0.43	799.4870[M-H-Ara(f)]$^-$, 637.4311[M-H-Ara(f)-Glc]$^-$, 475.3805[M-H-Ara(f)-2Glc]$^-$	Ginsenoside Re_4 [a]
R35	11.04	$C_{53}H_{90}O_{22}$	1077.5857	1.11	945.5451[M-H-Ara]$^-$, 799.4750[M-H-Ara-Rha]$^-$, 637.4280[M-H-Ara-Rha-Glc]$^-$, 475.3815[M-H-Ara-Rha-2Glc]$^-$	Floralginsenoside M/Floralginsenoside N
R36	11.13	$C_{42}H_{74}O_{14}$	847.5063 [b]	0.94	801.5004[M-H]$^-$, 655.4425[M-H-Rha]$^-$, 493.3891[M-H-Rha-Glc]$^-$	Ginsenoside Rf_2
R37	11.17	$C_{42}H_{72}O_{14}$	799.4828	-2.00	653.4296[M-H-Rha]$^-$, 635.4138[M-H-Rha-H_2O]$^-$, 491.3751[M-H-Rha-Glc]$^-$	Oxidated-protopanaxatriol+Glc+Rha
R38	11.17	$C_{42}H_{74}O_{15}$	863.4995 [b]	-1.04	817.4957[M-H]$^-$, 655.4329[M-H-Glc]$^-$, 493.3911[M-H-2Glc]$^-$	Double bond hydrated-protopanaxatriol+2Glc
R39	11.17	$C_{59}H_{98}O_{28}$	1299.6212 [b]	-0.69	1121.5668[M-H-Xyl/Ara]$^-$, 959.5083[M-H-Xyl/Ara-Glc]$^-$, 797.4700[M-H-Xyl/Ara-2Glc]$^-$, 635.4093[M-H-Xyl/Ara-3Glc]$^-$, 473.3625[M-H-Xyl/Ara-4Glc]$^-$	Dehydrogenated-protopanaxatriol+4Glc+Xyl/Ara
R40	11.18	$C_{48}H_{82}O_{19}$	961.5369	-0.31	799.4857[M-H-Glc]$^-$, 637.4313[M-H-2Glc]$^-$, 475.3779[M-H-3Glc]$^-$	20-Gluco-ginsenoside Rf [a]
R41	11.22	$C_{42}H_{72}O_{15}$	815.4801	0.98	653.4325[M-H-2Glc]$^-$, 491.3709[M-H-2Glc]$^-$	Ginsenoside Re_5 isomer/Ginsenjilinol isomer
R42	11.22	$C_{50}H_{84}O_{20}$	1003.5487	0.90	961.5366[M-H-Ac]$^-$,	Acetyl ginsenoside Re_1/ Acetyl

					799.4789[M-H-Ac-Glc]⁻, 637.4323[M-H-Ac-2Glc]⁻, 475.3793[M-H-Ac-3Glc]⁻	ginsenoside Re_2/ Acetyl ginsenoside Re_3/ Acetyl ginsenoside 20-Gluco-ginsenoside Rf/ Acetyl notoginsenoside N
R43	11.25	$C_{47}H_{80}O_{18}$	931.5271	0.54	637.4346[M-H-Xyl-Glc]⁻, 475.3808[M-H-Xyl-2Glc]⁻	Notoginsenoside R_1 isomer
R44	11.26	$C_{58}H_{98}O_{26}$	1209.6295	2.23	1077.5820[M-H-Xyl]⁻, 945.5355[M-H-Xyl-Ara]⁻, 783.5020[M-H-Xyl-Ara-Glc]⁻, 637.4327[M-H-Xyl-Ara-Glc-Rha]⁻, 475.3804[M-H-Xyl-Ara-2Glc-Rha]	Protopanaxatriol+2Glc+Rha+Xyl+Ara
R45	11.31	$C_{54}H_{92}O_{23}$	1107.5973	1.99	945.5414[M-H-Glc]⁻, 783.4889[M-H-2Glc]⁻, 637.4300[M-H-2Glc-Rha]⁻, 475.3807[M-H-3Glc-Rha]⁻	Protopanaxatriol+3Glc+Rha
R46	11.32	$C_{48}H_{82}O_{19}$	961.5381	0.94	799.4863[M-H-Glc]⁻, 637.4342[M-H-2Glc]⁻, 475.3794[M-H-3Glc]⁻,	Ginsenoside Re_1 [a]
R47	11.35	$C_{42}H_{74}O_{14}$	847.5060 [b]	0.59	801.4948[M-H]⁻, 655.4340[M-H-Rha]⁻, 493.3745[M-H-Rha-Glc]⁻	Ginsenoside Rf_2 isomer
R48	11.39	$C_{53}H_{90}O_{22}$	1077.5864	1.76	945.5461[M-H-Ara]⁻, 799.4789[M-H-Ara-Rha]⁻, 637.4276[M-H-Ara-Rha-Glc]⁻, 475.3737[M-H-Ara-Rha-2Glc]⁻	Floralginsenoside M/Floralginsenoside N
R49	11.43	$C_{54}H_{94}O_{25}$	1141.5999	-0.61	979.5508[M-H-Glc]⁻, 817.4980[M-H-2Glc]⁻, 799.4844[M-H-2Glc-H_2O]⁻, 655.4418[M-H-3Glc]⁻,	Double bond hydrated-protopanaxatriol+4Glc

					637.4325[M-H-3Glc-H_2O]$^-$, 493.3886[M-H-4Glc]$^-$	
R50	11.45	$C_{47}H_{80}O_{18}$	931.5268	0.21	799.4841[M-H-Xyl]$^-$, 637.4324[M-H-Xyl-Glc]$^-$, 475.3802[M-H-Xyl-2Glc]$^-$	Notoginsenoside R_1 [a]
R51	11.46	$C_{42}H_{70}O_{15}$	813.4651	1.84	651.4540[M-H-Glc]$^-$, 489.3578[M-H-2Glc]$^-$	Methyl etherified-protopanaxatriol+2Glc
R52	11.47	$C_{54}H_{92}O_{23}$	1107.5931	-1.81	945.5482[M-H-Glc]$^-$, 783.4904[M-H-2Glc]$^-$, 637.4229[M-H-2Glc-Rha]$^-$, 475.3813[M-H-3Glc-Rha]$^-$	Protopanaxatriol+3Glc+Rha
R53	11.55	$C_{36}H_{62}O_{10}$	699.4317 [b]	-0.43	491.3727[M-H-Glc]$^-$	Oxidated-protopanaxatriol+Glc
R54	11.60	$C_{48}H_{82}O_{19}$	961.5388	1.66	799.4866[M-H-Glc]$^-$, 637.4362[M-H-2Glc]$^-$, 475.3822[M-H-3Glc]$^-$	Ginsenoside Re_2 [a]
R55	11.60	$C_{53}H_{92}O_{24}$	1111.5915	1.35	979.5418[M-H-Xyl/Ara]$^-$, 817.4936[M-H-Xyl/Ara-Glc]$^-$, 799.4843[M-H-Xyl/Ara-Glc-H_2O]$^-$, 655.4416[M-H-Xyl/Ara-2Glc]$^-$, 637.4308[M-H-Xyl/Ara-2Glc-H_2O]$^-$, 493.3888[M-H-Xyl/Ara-3Glc]$^-$	Double bond hydrated-protopanaxatriol+3Glc+Xyl/Ara
R56	11.62	$C_{51}H_{84}O_{19}$	1045.5572 [b]	-1.05	931.5161[M-H-(*E*)-but-2-enoyl]$^-$, 799.4856[M-H-(*E*)-but-2-enoyl-Ara(f)/Xyl]$^-$, 637.4326[M-H-(*E*)-but-2-enoyl-Ara(f)/Xyl-Glc]$^-$, 475.3801[M-H-(*E*)-but-2-enoyl-Ara(f)/Xyl-2Glc]$^-$	(*E*)-But-2-enoyl-ginsenoside Re_4/(*E*)-But-2-enoyl-notoginsenoside R_1
R57	11.71	$C_{42}H_{72}O_{15}$	815.4794	0.12	653.4219[M-H-2Glc]$^-$，	Ginsenoside Re_5 isomer/Ginsenjilinol

					491.3742[M-H-2Glc]-	isomer
R58	11.73	$C_{53}H_{90}O_{22}$	1077.5830	-1.39	945.5437[M-H-Ara]-, 799.4862[M-H-Ara-Rha]-, 637.4237[M-H-Ara-Rha-Glc]-, 475.3768[M-H-Ara-Rha-2Glc]-	Floralginsenoside M isomer/Floralginsenoside N isomer
R59	11.81	$C_{47}H_{80}O_{18}$	931.5274	0.86	637.4318[M-H-Xyl-Glc]-, 475.3796[M-H-Xyl-2Glc]-	Notoginsenoside R_1 isomer
R60	11.89	$C_{54}H_{92}O_{23}$	1107.5970	1.72	945.5467[M-H-Glc]-, 783.4889[M-H-2Glc]-, 637.430[M-H-2Glc-Rha]-, 475.3810[M-H-3Glc-Rha]-	Protopanaxatriol+3 Glc+Rha
R61	11.89	$C_{51}H_{84}O_{22}$	1047.5377	0.10	961.5388[M-H-Malonyl]-, 799.4798[M-H-Malonyl-Glc]-, 637.4352[M-H-Malonyl-2Glc]-, 475.3803[M-H-Malonyl-3Glc]-	Malonyl-20-Gluco-ginsenoside Rf
R62	11.90	$C_{42}H_{70}O_{15}$	859.4703 [b]	1.40	489.3678[M-H-2Glc]-	Methyl etherified-protopanaxatriol+2Glc
R63	11.94	$C_{42}H_{74}O_{15}$	863.4990 [b]	-1.62	817.4906[M-H]-, 493.3920[M-H-2Glc]-	Double bond hydrated-protopanaxatriol+2Glc
R64	11.98	$C_{50}H_{84}O_{20}$	1003.5478	0.00	961.5382[M-H-Ac]-, 799.4834[M-H-Ac-Glc]-, 637.4323[M-H-Ac-2Glc]-, 475.3794[M-H-Ac-3Glc]-	Acetyl ginsenoside Re_1/ Acetyl ginsenoside Re_2/ Acetyl ginsenoside Re_3/ Acetyl ginsenoside 20-Gluco-ginsenoside Rf/ Acetyl notoginsenoside N
R65	11.99	$C_{54}H_{90}O_{25}$	1137.5707	1.23	961.5355[M-H-Glu A]-, 799.4926[M-H-Glu A-Glc]-, 637.4364[M-H-Glu A-2Glc]-,	Protopanaxatriol+ Glu A+3Glc

					475.3810[M-H-Glu A-3Glc]-	
R66	12.01	$C_{54}H_{92}O_{24}$	1123.5904	0.36	961.5287[M-H-Glc]-, 799.4835[M-H-2Glc]-, 637.4333[M-H-3Glc]-, 475.3820[M-H-4Glc]-	Koryoginsenoside R_2
R67	12.05	$C_{50}H_{90}O_{27}$	1121.5595	0.36	959.5172[M-H-Glc]-, 797.4690[M-H-2Glc]-, 635.4152[M-H-3Glc]-, 473.3636[M-H-4Glc]-	Dehydrogenated-protopanaxatriol+4Glc
R68	12.08	$C_{54}H_{92}O_{23}$	1107.5946	-0.45	945.5471[M-H-Glc]-, 783.4981[M-H-2Glc]-, 637.4362[M-H-2Glc-Rha]-, 475.3792[M-H-3Glc-Rha]-	Protopanaxatriol+3Glc+Rha
R69	12.09	$C_{48}H_{82}O_{19}$	961.5372	0.00	799.4891[M-H-Glc]-, 637.4326[M-H-2Glc]-, 475.3784[M-H-3Glc]-	Notoginsenoside N isomer
R70	12.12	$C_{42}H_{72}O_{15}$	815.4800	0.86	653.4303[M-H-2Glc]-, 491.3732[M-H-2Glc]-	Ginsenjilinol [a]
R71	12.12	$C_{55}H_{94}O_{25}$	1153.6014	0.69	1111.5779[M-H-Ac]-, 1093.5718[M-H-Ac-H_2O]-, 817.4957[M-H-Ac-Xyl/Ara-Glc]-, 799.4856[M-H-Ac-Xyl/Ara-Glc-H_2O]-, 781.4699[M-H-Ac-Xyl/Ara-Glc-2H_2O]-, 655.4407[M-H-Ac-Xyl/Ara-2Glc]-, 637.4338[M-H-Ac-Xyl/Ara-2Glc-H_2O]-, 619.4264[M-H-Ac-Xyl/Ara-2Glc-2H_2O]-, 493.3902[M-H-Ac-Xyl/Ara-3Glc]-, 475.3781[M-H-Ac-Xyl/Ara-3Glc-H_2O]-	Double bond hydrated-protopanaxatriol+2Glc+Acetyl Glc+Xyl/Ara

R72	12.13	$C_{54}H_{92}O_{24}$	1123.5907	0.62	961.5385[M-H-Glc]⁻, 799.4851[M-H-2Glc]⁻, 637.4326[M-H-3Glc]⁻, 475.3795[M-H-4Glc]⁻	Koryoginsenoside R_2 isomer
R73	12.16	$C_{42}H_{72}O_{14}$	799.4850	0.75	637.4320[M-H-Glc]⁻, 475.3784[M-H-2Glc]⁻	Ginsenoside Rg_1 [a]
R74	12.20	$C_{48}H_{82}O_{18}$	945.5421	-0.21	783.4882[M-H-Glc]⁻, 637.4333[M-H-Glc-Rha]⁻, 475.3796[M-H-2Glc-Rha]⁻	Ginsenoside Re [a]
R75	12.23	$C_{48}H_{84}O_{20}$	1025.5533 [b]	0.10	979.5458[M-H]⁻, 817.4959[M-H-Glc]⁻, 799.4861[M-H-Glc-H_2O]⁻, 655.4432[M-H-2Glc]⁻, 637.4311[M-H-2Glc-H_2O]⁻, 619.4230[M-H-2Glc-2H_2O]⁻, 493.3889[M-H-3Glc]⁻, 475.3819[M-H-3Glc-H_2O]⁻	Double bond hydrated-protopanaxatriol+3Glc
R76	12.35	$C_{53}H_{92}O_{24}$	1111.5895	-0.45	979.5455[M-H-Xyl/Ara]⁻, 817.4952[M-H-Xyl/Ara-Glc]⁻, 799.4852[M-H-Xyl/Ara-Glc-H_2O]⁻, 655.4422[M-H-Xyl/Ara-2Glc]⁻, 637.4312[M-H-Xyl/Ara-2Glc-H_2O]⁻, 493.3893[M-H-Xyl/Ara-3Glc]⁻	Double bond hydrated-protopanaxatriol+3Glc+Xyl/Ara
R77	12.36	$C_{48}H_{84}O_{20}$	1025.5522 [b]	-0.98	979.5372[M-H]⁻, 817.4875[M-H-Glc]⁻, 799.4833[M-H-Glc-H_2O]⁻, 655.4424[M-H-2Glc]⁻, 637.4407[M-H-2Glc-H_2O]⁻,	Double bond hydrated-protopanaxatriol+3Glc

					619.4232[M-H-2Glc-2H_2O]$^-$, 493.3875[M-H-3Glc]$^-$, 475.3818[M-H-3Glc-H_2O]$^-$	
R78	12.36	$C_{56}H_{94}O_{27}$	1197.5901	-0.25	1111.5878[M-H-Malonyl]$^-$, 817.4951[M-H-Malonyl-Xyl/Ara-Glc]$^-$, 799.4863[M-H-Malonyl-Xyl/Ara-Glc-H_2O]$^-$, 655.4430[M-H-Malonyl-Xyl/Ara-2Glc]$^-$, 637.4326[M-H-Malonyl-Xyl/Ara-2Glc-H_2O]$^-$, 493.3820[M-H-Malonyl-Xyl/Ara-3Glc]$^-$,	Double bond hydrated-protopanaxatriol+2Glc+Malonyl Glc+Xyl/Ara
R79	12.38	$C_{48}H_{82}O_{18}$	945.5444	2.22	783.4899[M-H-Glc]$^-$, 637.4322[M-H-Glc-Rha]$^-$, 475.3793[M-H-2Glc-Rha]$^-$	Ginsenoside Re isomer
R80	12.47	$C_{57}H_{92}O_{27}$	1207.5728	-1.49	1121.5715[M-H-Malonyl]$^-$, 959.5240[M-H-Malonyl-Glc]$^-$, 797.4722[M-H-Malonyl-2Glc]$^-$, 473.3679[M-H-Malonyl-4Glc]$^-$	Dehydrogenated-protopanaxatriol+3Glc+Malonyl Glc
R81	12.53	$C_{47}H_{80}O_{18}$	931.5276	1.07	637.4321[M-H-Xyl-Glc]$^-$, 475.3806[M-H-Xyl-2Glc]$^-$	Notoginsenoside R_1 isomer
R82	12.53	$C_{56}H_{96}O_{26}$	1183.6119	0.59	1141.6007[M-H-Ac]$^-$, 1123.5983[M-H-Ac-H_2O]$^-$, 817.4993[M-H-Ac-2Glc]$^-$, 799.4839[M-H-Ac-2Glc-H_2O]$^-$, 655.4398[M-H-Ac-3Glc]$^-$, 637.4357[M-H-Ac-3Glc-H_2O]$^-$, 493.3891[M-H-Ac-4Glc]$^-$	Double bond hydrated-protopanaxatriol+4Glc+Acetyl Glc

R83	12.55	$C_{53}H_{90}O_{22}$	1077.5850	0.46	945.5454[M-H-Ara]-, 799.4864[M-H-Ara-Rha]-, 637.4312[M-H-Ara-Rha-Glc]-, 475.3795[M-H-Ara-Rha-2Glc]-	Floralginsenoside M isomer/Floralginsenoside N isomer
R84	12.56	$C_{53}H_{90}O_{23}$	1093.5801	0.55	799.4872[M-H-Ara-Glc]-, 637.4290[M-H-Ara-2Glc]-, 475.3759[M-H-Ara-3Glc]-	Floralginsenoside P/isomer
R85	12.58	$C_{44}H_{74}O_{15}$	841.4953	0.48	799.4750[M-H-Ac]-, 653.4247[M-H-Ac-Rha]-, 635.4170[M-H-Ac-Rha-H_2O]-, 491.3756[M-H-Ac-Rha-Glc]-	Oxidated-protopanaxatriol+Acetyl Glc+Rha
R86	12.59	$C_{51}H_{84}O_{22}$	1047.5378	0.19	961.5299[M-H-Malonyl]-, 799.4873[M-H-Malonyl-Glc]-, 637.4313[M-H-Malonyl-2Glc]-, 475.3788[M-H-Malonyl-3Glc]-	Malonyl-ginsenoside Re_1
R87	12.62	$C_{48}H_{76}O_{21}$	987.4805	0.41	825.4283[M-H-Glc]-, 807.4205[M-H-Glc-H_2O]-, 487.3440[M-H-2Glc-Glu A]-	Methyl etherified+Dehydrogenated-protopanaxatriol+2Glc+Glu A
R88	12.64	$C_{47}H_{80}O_{18}$	931.5265	-0.11	637.4303[M-H-Xyl-Glc]-, 475.3780[M-H-Xyl-2Glc]-	Notoginsenoside R_1 isomer
R89	12.66	$C_{53}H_{90}O_{22}$	1077.5859	1.30	945.5455[M-H-Ara]-, 799.4836[M-H-Ara-Rha]-, 637.4388[M-H-Ara-Rha-Glc]-, 475.3786[M-H-Ara-Rha-2Glc]-	Floralginsenoside M isomer/Floralginsenoside N isomer
R90	12.71	$C_{50}H_{86}O_{21}$	1021.5587	0.39	979.5484[M-H-Ac]-, 817.4941[M-H-Ac-Glc]-, 799.4849[M-H-Ac-Glc-H_2O]-, 655.4421[M-H-Ac-2Glc]-, 637.4341[M-H-Ac-2Glc-H_2O]-, 619.4265[M-H-Ac-2Glc-2H_2O]-,	Double bond hydrated-protopanaxatriol+2Glc+Acetyl Glc

					493.3891[M-H-Ac-3Glc]$^-$, 475.3755[M-H-Ac-3Glc-H_2O]$^-$	
R91	12.72	$C_{48}H_{84}O_{19}$	1009.5579 [b]	-0.40	963.5515[M-H]$^-$, 801.4996[M-H-Glc]$^-$, 783.4870[M-H-Glc-H_2O]$^-$, 639.4477[M-H-2Glc]$^-$, 621.4380[M-H-2Glc-H_2O]$^-$, 477.3950[M-H-3Glc]$^-$	Dihydrogenated-protopanaxatriol+3Glc
R92	12.77	$C_{51}H_{84}O_{21}$	1031.5427	0.00	945.5460[M-H-Malonyl]$^-$, 783.4988[M-H-Malonyl-Glc]$^-$, 637.4340[M-H-Malonyl-Glc-Rha]$^-$, 475.3803[M-H-Malonyl-2Glc- Rha]$^-$	Malonyl-ginsenoside Re isomer
R93	12.81	$C_{50}H_{86}O_{21}$	1021.5568	-1.47	979.5511[M-H-Ac]$^-$, 799.4850[M-H-Ac-Glc-H_2O]$^-$, 655.4395[M-H-Ac-2Glc]$^-$, 637.4366[M-H-Ac-2Glc-H_2O]$^-$, 619.4227[M-H-Ac-2Glc-2H_2O]$^-$, 493.3882[M-H-Ac-3Glc]$^-$, 475.1438[M-H-Ac-3Glc-H_2O]$^-$	Double bond hydrated-protopanaxatriol+2Glc+Acetyl Glc
R94	12.86	$C_{44}H_{74}O_{16}$	903.4963 [b]	1.11	815.4803[M-H-Ac]$^-$, 653.4260[M-H-Ac-Glc]$^-$, 491.3755[M-H-Ac-2Glc]$^-$	Oxidated-protopanaxatriol+Glc+Acetyl Glc
R95	12.87	$C_{51}H_{86}O_{23}$	1065.5492	0.94	979.5419[M-H-Malonyl]$^-$, 817.4968[M-H-Malonyl-Glc]$^-$, 799.4857[M-H-Malonyl-Glc-H_2O]$^-$, 655.4406[M-H-Malonyl-2Glc]$^-$, 637.4335[M-H-Malonyl-2Glc-H_2O]$^-$, 619.4207[M-H-Malonyl-2Glc-2H_2O]$^-$,	Double bond hydrated-protopanaxatriol+2Glc+Malonyl Glc

					493.3882[M-H-Malonyl-3Glc]$^-$, 475.3813[M-H-Malonyl-3Glc-H_2O]$^-$	
R96	12.90	$C_{48}H_{82}O_{19}$	961.5375	0.31	799.3901[M-H-Glc]$^-$, 637.3561[M-H-2Glc]$^-$, 475.3701[M-H-3Glc]$^-$	Notoginsenoside N isomer
R97	12.90	$C_{44}H_{74}O_{15}$	841.4947	1.17	637.4321[M-H-Ac-Glc]$^-$, 475.3799[M-H-Ac-2Glc]$^-$	Acetyl-ginsenoside Rg_1
R98	12.90	$C_{45}H_{74}O_{17}$	885.4837	-1.24	637.4315[M-H-Malonyl-Glc]$^-$, 475.3835[M-H-Malonyl-2Glc]$^-$	Malonyl-ginsenoside Rg_1
R99	12.93	$C_{42}H_{72}O_{15}$	815.4800	0.86	815.4816[M-H]$^-$, 653.4277[M-H-2Glc]$^-$, 491.3759[M-H-2Glc]$^-$	Ginsenoside Re_5 isomer/Ginsenjilinol isomer
R100	12.99	$C_{42}H_{72}O_{14}$	799.4854	1.25	637.4279[M-H-Glc]$^-$, 475.3794[M-H-2Glc]$^-$	Ginsenoside Rg_1 isomer
R101	13.05	$C_{51}H_{84}O_{21}$	1031.5419	-0.78	945.5322[M-H-Malonyl]$^-$, 783.4980[M-H-Malonyl-Glc]$^-$, 637.4324[M-H-Malonyl-Glc-Rha]$^-$, 475.3753[M-H-Malonyl-2Glc- Rha]$^-$	Malonyl-ginsenoside Re isomer
R102	13.05	$C_{53}H_{88}O_{23}$	1137.5687 [b]	-0.53	1091.5598[M-H]$^-$, 959.5298[M-H-Xyl/Ara]$^-$, 797.4694[M-H-Xyl/Ara-Glc]$^-$, 779.4532[M-H-Xyl/Ara-Glc-H_2O]$^-$, 635.4178[M-H-Xyl/Ara-2Glc]$^-$, 473.3620[M-H-Xyl/Ara-3Glc]$^-$	Dehydrogenated-protopanaxatriol+3Glc +Xyl/Ara
R103	13.07	$C_{54}H_{92}O_{24}$	1123.5906	0.53	961.5416[M-H-Glc]$^-$, 799.4856[M-H-2Glc]$^-$, 637.4343[M-H-3Glc]$^-$, 475.3789[M-H-4Glc]$^-$	Koryoginsenoside R_2 isomer

R104	13.07	$C_{47}H_{80}O_{18}$	931.5257	-0.97	799.4843[M-H-Xyl]-, 637.4326[M-H-Xyl-Glc]-, 475.3800[M-H-Xyl-2Glc]-	Notoginsenoside R1 isomer
R105	13.08	$C_{48}H_{82}O_{18}$	945.5401	-2.33	783.4896[M-H-Glc]-, 637.4319[M-H-Glc-Rha]-, 475.3794[M-H-2Glc-Rha]-	Ginsenoside Re isomer
R106	13.08	$C_{50}H_{84}O_{19}$	987.5532	0.30	945.5494[M-H-Ac]-, 799.4935[M-H-Ac-Rha]-, 637.4324[M-H-Ac-Rha-Glc]-, 475.3795[M-H-Ac-Rha-2Glc]-	Acetyl-ginsenoside Re
R107	13.11	$C_{53}H_{90}O_{23}$	1093.5809	1.28	799.4849[M-H-Ara-Glc]-, 637.4339[M-H-Ara-2Glc]-, 475.3758[M-H-Ara-3Glc]-	Floralginsenoside P/isomer
R108	13.13	$C_{38}H_{64}O_{11}$	695.4376	0.89	491.3710[M-H-Ac-Glc]-	Oxidated-protopanaxatriol+Acetyl Glc
R109	13.15	$C_{53}H_{86}O_{22}$	1073.5525	-0.65	987.4690[M-H-Malonyl]-, 945.5401[M-H-Malonyl-Ac]-, 783.4783[M-H-Malonyl-Ac-Glc]-, 637.4191[M-H-Malonyl-Ac-Glc-Rha]-, 475.3823[M-H-Malonyl-Ac-2Glc-Rha]-	Acetyl malonyl-ginsenoside Re
R110	13.17	$C_{51}H_{84}O_{22}$	1047.5388	1.15	961.5355[M-H-Malonyl]-, 799.4765[M-H-Malonyl-Glc]-, 637.4330[M-H-Malonyl-2Glc]-, 475.3807[M-H-Malonyl-3Glc]-	Malonyl-ginsenoside Re2
R111	13.17	$C_{50}H_{84}O_{21}$	1019.5435 [b]	0.78	637.4393[M-H-Ac-Ara-Glc]-, 475.3796[M-H-Ac-Ara-2Glc]-	Acetyl-ginsenoside Re4
R112	13.24	$C_{54}H_{92}O_{24}$	1123.5889	-0.98	961.5415[M-H-Glc]-, 799.4851[M-H-2Glc]-, 637.4243[M-H-3Glc]-, 475.3768[M-H-4Glc]-	Koryoginsenoside R2 isomer

R113	13.37	$C_{44}H_{74}O_{15}$	841.4953	1.95	637.4342[M-H-Ac-Glc]$^-$, 475.3808[M-H-Ac-2Glc]$^-$	Acetyl-ginsenoside Rg_1
R114	13.42	$C_{50}H_{84}O_{19}$	987.5530	0.10	945.5394[M-H-Ac]$^-$, 799.4955[M-H-Ac-Rha]$^-$, 637.4340[M-H-Ac-Rha-Glc]$^-$, 475.3804[M-H-Ac-Rha-2Glc]$^-$	Acetyl-ginsenoside Re
R115	13.44	$C_{48}H_{82}O_{18}$	945.5444	2.22	783.4913[M-H-Glc]$^-$, 637.4334[M-H-Glc-Rha]$^-$, 475.3796[M-H-2Glc-Rha]$^-$	Ginsenoside Re isomer
R116	13.52	$C_{53}H_{90}O_{23}$	1093.5809	1.28	799.4889[M-H-Ara-Glc]$^-$, 637.4341[M-H-Ara-2Glc]$^-$, 475.3742[M-H-Ara-3Glc]$^-$	Floralginsenoside P/isomer
R117	13.54	$C_{51}H_{84}O_{21}$	1031.5422	-0.48	945.5392[M-H-Malonyl]$^-$, 783.4880[M-H-Malonyl-Glc]$^-$, 637.4326[M-H-Malonyl-Glc-Rha]$^-$, 475.3799[M-H-Malonyl-2Glc- Rha]$^-$	Malonyl-ginsenosid e Re isomer
R118	13.54	$C_{50}H_{84}O_{19}$	987.5533	0.41	945.5435[M-H-Ac]$^-$, 783.4855[M-H-Ac-Glc]$^-$, 637.4317[M-H-Ac-Glc-Rha]$^-$, 475.3792[M-H-Ac-2Glc-Rha]$^-$	Acetyl-ginsenoside Re
R119	13.58	$C_{42}H_{72}O_{15}$	815.4792	-0.12	653.4335[M-H-2Glc]$^-$, 491.3724[M-H-2Glc]$^-$	Ginsenoside Re_5 isomer/Ginsenjilinol isomer
R120	13.58	$C_{53}H_{90}O_{25}$	1125.5706 [b]	1.15	1079.5618[M-H]$^-$, 947.5281[M-H-Xyl/Ara]$^-$, 785.4716[M-H-Xyl/Ara-Glc]$^-$, 767.4621[M-H-Xyl/Ara-Glc-H_2O]$^-$, 623.4171[M-H-Xyl/Ara-2Glc]$^-$, 461.3641[M-H-Xyl/Ara-3Glc]$^-$	Dihydrogenated-pr otopanaxadiol+Xyl/ Ara+3Glc
R121	13.63	$C_{42}H_{72}O_{14}$	845.4904 [b]	0.59	799.4853[M-H]$^-$, 653.4266[M-H-Rha]$^-$, 491.3747[M-H-Rha-Glc]$^-$	Oxidated-protopan axatriol+Glc+Rha

R122	13.64	$C_{45}H_{74}O_{17}$	885.4843	-0.56	637.4318[M-H-Malonyl-Glc]-, 475.3798[M-H-Malonyl-2Glc]-	Malonyl-ginsenoside Rf
R123	13.65	$C_{42}H_{72}O_{13}$	783.4904	1.15	637.4343[M-H-Rha]-, 475.3813[M-H-Rha-Glc]-	Ginsenoside Rg_2 isomer
R124	13.70	$C_{54}H_{92}O_{24}$	1123.5895	-0.45	961.5392[M-H-Glc]-, 799.4860[M-H-2Glc]-, 637.4319[M-H-3Glc]-, 475.3795[M-H-4Glc]-	Koryoginsenoside R_2 isomer
R125	13.71	$C_{56}H_{92}O_{26}$	1179.5798	0.00	1093.5884[M-H-Malonyl]-, 961.5355[M-H-Malonyl-Ara/Xyl]-, 799.4839[M-H-Malonyl-Ara/Xyl-Glc]-, 637.4319[M-H-Malonyl-Ara/Xyl-2Glc]-, 475.3779[M-H-Malonyl-Ara/Xyl-3Glc]-	Protopanaxatriol+ Ara/Xyl+2Glc+ Malonyl Glc
R126	13.71	$C_{42}H_{70}O_{15}$	859.4701 [b]	1.16	813.4616[M-H]-, 651.4153[M-H-Glc]-, 489.3582[M-H-2Glc]-	Methyl etherified-protopanaxatriol+2Glc
R127	13.85	$C_{50}H_{84}O_{19}$	987.5536	0.71	945.5443[M-H-Ac]-, 799.4857[M-H-Ac-Rha]-, 783.4902[M-H-Ac-Glc]-, 637.4315[M-H-Ac-Glc-Rha]-, 475.3795[M-H-Ac-2Glc-Rha]-	Acetyl-ginsenoside Re
R128	13.85	$C_{53}H_{90}O_{23}$	1093.5815	1.83	799.4887[M-H-Ara-Glc]-, 637.4340[M-H-Ara-2Glc]-, 475.3739[M-H-Ara-3Glc]-	Floralginsenoside P/isomer
R129	13.87	$C_{48}H_{80}O_{19}$	959.5223	0.73	797.4679[M-H-Glc]-, 779.4590[M-H-Glc-H_2O]-, 635.4178[M-H-2Glc]-, 617.4015[M-H-2Glc-H_2O]-, 473.3635[M-H-3Glc]-	Dehydrogenated-protopanaxatriol+3Glc

R130	13.87	$C_{51}H_{86}O_{23}$	1065.5499	1.60	979.5517[M-H-Malonyl]$^-$, 817.5003[M-H-Malonyl-Glc]$^-$, 799.4806[M-H-Malonyl-Glc-H_2O]$^-$, 655.4362[M-H-Malonyl-2Glc]$^-$, 637.4373[M-H-Malonyl-2Glc-H_2O]$^-$, 493.3889[M-H-Malonyl-3Glc]$^-$	Double bond hydrated-protopanaxatriol+2Glc+Malonyl Glc
R131	13.88	$C_{48}H_{82}O_{18}$	945.5433	1.06	783.4896[M-H-Glc]$^-$, 637.4319[M-H-Glc-Rha]$^-$, 475.3794[M-H-2Glc-Rha]$^-$	Ginsenoside Re isomer
R132	13.88	$C_{50}H_{84}O_{21}$	1019.5422 [b]	-0.49	637.4276[M-H-Ac-Xyl/Ara-Glc]$^-$, 475.3781[M-H-Ac-Xyl/Ara-2Glc]$^-$	Acetyl-ginsenoside Re_4 isomer/ Acetyl-notoginsenoside R_1 isomer
R133	13.90	$C_{45}H_{72}O_{17}$	883.4695	0.45	635.4197[M-H-Malonyl-Glc]$^-$, 617.4048[M-H-Malonyl-Glc-H_2O]$^-$, 473.3653[M-H-Malonyl-2Glc]$^-$	Dehydrogenated-protopanaxatriol+Glc+ Malonyl Glc
R134	13.94	$C_{58}H_{98}O_{27}$	1225.6230	1.06	1093.5801[M-H-Xyl]$^-$, 961.5365[M-H-Xyl-Ara]$^-$, 799.4849[M-H-Xyl-Ara-Glc]$^-$, 781.4739[M-H-Xyl-Ara-Glc-H_2O]$^-$, 637.4312[M-H-Xyl-Ara-2Glc]$^-$, 619.4229[M-H-Xyl-Ara-2Glc-H_2O]$^-$, 475.3795[M-H-Xyl-Ara-3Glc]$^-$	Protopanaxatriol+3Glc+Xyl+Ara
R135	13.95	$C_{48}H_{82}O_{18}$	945.5446	2.43	783.4995 [M-H-Glc]$^-$, 637.4312[M-H-Glc-Rha]$^-$, 475.3776[M-H-2Glc-Rha]$^-$	Ginsenoside Re isomer
R136	14.01	$C_{56}H_{92}O_{26}$	1179.5801	0.25	1093.5836[M-H-Malonyl]$^-$, 961.5344[M-H-Malonyl-Ara/Xyl]$^-$, 799.4829[M-H-Malonyl-Ara/Xyl-Glc]$^-$, 637.4318[M-H-Malonyl-Ara/Xyl-2Glc]$^-$	Protopanaxatriol+ Ara/Xyl+2Glc+ Malonyl Glc

					, 475.3758[M-H-Malonyl-Ara/Xyl-3Glc]$^{-}$	
R137	14.05	$C_{58}H_{98}O_{27}$	1225.6222	0.41	1093.5773[M-H-Xyl]$^{-}$, 799.4880[M-H-Xyl-Ara-Glc]$^{-}$, 637.4340[M-H-Xyl-Ara-2Glc]$^{-}$, 475.3803[M-H-Xyl-Ara-3Glc]$^{-}$	Protopanaxatriol+3 Glc+Xyl+Ara
R138	14.06	$C_{54}H_{90}O_{24}$	1121.5742	-0.18	1079.5619[M-H-Ac]$^{-}$, 947.5371[M-H-Ac-Xyl/Ara]$^{-}$, 785.4726[M-H-Ac-Xyl/Ara-Glc]$^{-}$, 767.4586[M-H-Ac-Xyl/Ara-Glc-H_2O]$^{-}$, 623.4160[M-H-Ac-Xyl/Ara-2Glc]$^{-}$, 461.3647[M-H-Ac-Xyl/Ara-3Glc]$^{-}$	Dihydrogenated-protopanaxadiol+Xyl/Ara+2Glc+Acetyl Glc
R139	14.10	$C_{42}H_{72}O_{15}$	815.4791	-0.25	653.4305[M-H-2Glc]$^{-}$, 491.3717[M-H-2Glc]$^{-}$	Ginsenoside Re_5 isomer/Ginsenjilinol isomer
R140	14.11	$C_{53}H_{90}O_{23}$	1093.5790	-0.46	799.4835[M-H-Ara-Glc]$^{-}$, 637.4334[M-H-Ara-2Glc]$^{-}$, 475.3791[M-H-Ara-3Glc]$^{-}$	Floralginsenoside P/isomer
R141	14.12	$C_{50}H_{84}O_{19}$	987.5519	-1.01	637.4330[M-H-Ac-Glc-Rha]$^{-}$, 475.3794[M-H-Ac-2Glc-Rha]$^{-}$	Acetyl-ginsenoside Re
R142	14.12	$C_{48}H_{82}O_{19}$	961.5377	0.52	799.4821[M-H-Glc]$^{-}$, 637.4316[M-H-2Glc]$^{-}$, 475.3820[M-H-3Glc]$^{-}$	Notoginsenoside N
R143	14.18	$C_{51}H_{84}O_{21}$	1031.5450	2.23	945.5383[M-H-Malonyl]$^{-}$, 783.4823[M-H-Malonyl-Glc]$^{-}$, 637.4324[M-H-Malonyl-Glc-Rha]$^{-}$, 475.3781[M-H-Malonyl-2Glc- Rha]$^{-}$	Malonyl-ginsenoside Re isomer
R144	14.22	$C_{46}H_{76}O_{16}$	883.5067	1.36	637.4298[M-H-2Ac-Glc]$^{-}$, 619.4203[M-H-2Ac-Glc-H_2O]$^{-}$, 475.3790[M-H-2Ac-2Glc]$^{-}$	Protopanaxatriol+Diacetyl+2Glc

R145	14.25	$C_{51}H_{84}O_{22}$	1047.5388	1.15	961.5351[M-H-Malonyl]$^-$, 799.4763[M-H-Malonyl-Glc]$^-$, 637.4323[M-H-Malonyl-2Glc]$^-$, 475.3787[M-H-Malonyl-3Glc]$^-$	Malonyl-notoginsenoside N isomer
R146	14.28	$C_{53}H_{86}O_{22}$	1073.5546	1.30	945.5434[M-H-Malonyl-Ac]$^-$, 783.4911[M-H-Malonyl-Ac-Glc]$^-$, 637.4317[M-H-Malonyl-Ac-Glc-Rha]$^-$, 475.3812[M-H-Malonyl-Ac-2Glc-Rha]$^-$	Acetyl malonyl-ginsenoside Re
R147	14.31	$C_{56}H_{92}O_{26}$	1179.5796	-0.17	1093.5822[M-H-Malonyl]$^-$, 961.5355[M-H-Malonyl-Ara/Xyl]$^-$, 799.4937[M-H-Malonyl-Ara/Xyl-Glc]$^-$, 637.4329[M-H-Malonyl-Ara/Xyl-2Glc]$^-$, 475.3789[M-H-Malonyl-Ara/Xyl-3Glc]$^-$	Protopanaxatriol+ Ara/Xyl+2Glc+ Malonyl Glc
R148	14.31	$C_{53}H_{92}O_{23}$	1141.5999 [b]	-0.61	1095.5909[M-H]$^-$, 963.5483[M-H-Xyl/Ara]$^-$, 801.4924[M-H-Xyl/Ara-Glc]$^-$, 639.4419[M-H-Xyl/Ara-2Glc]$^-$, 477.3871[M-H-Xyl/Ara-3Glc]$^-$	Dihydrogenated-protopanaxatriol+3Glc +Xyl/Ara
R149	14.34	$C_{48}H_{82}O_{19}$	961.5379	0.73	799.4813[M-H-Glc]$^-$, 637.4316[M-H-2Glc]$^-$, 475.3793[M-H-3Glc]$^-$	Vina-ginsenoside R_4
R150	14.38	$C_{51}H_{84}O_{21}$	1031.5441	1.36	783.4922[M-H-Malonyl-Glc]$^-$, 637.4341[M-H-Malonyl-Glc-Rha]$^-$, 475.3785 [M-H-Malonyl-2Glc- Rha]$^-$	Malonyl-ginsenoside Re isomer
R151	14.39	$C_{50}H_{84}O_{19}$	987.5522	-0.71	945.5436[M-H-Ac]$^-$, 783.4905[M-H-Ac-Glc]$^-$, 637.4327[M-H-Ac-Glc-Rha]$^-$, 475.3798[M-H-Ac-2Glc-Rha]$^-$	Acetyl-ginsenoside Re

R152	14.40	$C_{54}H_{92}O_{24}$	1123.5909	0.80	961.5357[M-H-Glc]-, 799.4830 [M-H-2Glc]-, 637.4338[M-H-3Glc]-, 475.3849[M-H-4Glc]-	Koryoginsenoside R_2 isomer
R153	14.43	$C_{42}H_{72}O_{15}$	815.4790	-0.37	653.4233[M-H-2Glc]-, 491.3729[M-H-2Glc]-	Ginsenoside Re_5 isomer/Ginsenjilinol isomer
R154	14.49	$C_{53}H_{90}O_{23}$	1093.5813	1.65	799.4787[M-H-Ara-Glc]-, 637.4315[M-H-Ara-2Glc]-, 475.3788[M-H-Ara-3Glc]-	Floralginsenoside P/isomer
R155	14.60	$C_{46}H_{76}O_{16}$	929.5119 [b]	0.97	815.4778[M-H-(*E*)-but-2-enoyl]-, 653.4285[M-H-(*E*)-but-2-enoyl-Glc]-, 491.3733[M-H-(*E*)-but-2-enoyl-2Glc]-	(*E*)-But-2-enoyl Ginsenoside Re_5
R156	14.62	$C_{53}H_{92}O_{23}$	1141.5990 [b]	-1.40	1095.5920[M-H]-, 963.5554[M-H-Xyl/Ara]-, 801.4981[M-H-Xyl/Ara-Glc]-, 639.4517[M-H-Xyl/Ara-2Glc]-, 477.3948[M-H-Xyl/Ara-3Glc]-	Dihydrogenated-protopanaxatriol+3Glc +Xyl/Ara
R157	14.64	$C_{42}H_{72}O_{14}$	799.4852	1.00	637.4333[M-H-Glc]-, 475.3801[M-H-2Glc]-	Ginsenoside Rg_1 isomer
R158	14.64	$C_{42}H_{74}O_{15}$	863.5009 [b]	0.58	817.4905[M-H]-, 655.4430[M-H-Glc]-, 493.3898[M-H-2Glc]-	Double bond hydrated-protopanaxatriol+2Glc
R159	14.69	$C_{50}H_{90}O_{27}$	1121.5579	-1.07	959.5171[M-H-Glc]-, 797.4680[M-H-2Glc]-, 779.4545[M-H-2Glc-H_2O]-, 635.4135[M-H-3Glc]-, 617.4081[M-H-3Glc-H_2O]-, 473.3639[M-H-4Glc]-,	Dehydrogenated-protopanaxatriol+4Glc

					455.3554[M-H-4Glc-H_2O]$^-$	
R160	14.79	$C_{54}H_{90}O_{23}$	1105.5779	-1.45	943.5278[M-H-Glc]$^-$, 781.4628[M-H-2Glc]$^-$, 763.4622[M-H-2Glc-H_2O]$^-$, 619.4229[M-H-3Glc]$^-$, 601.4104[M-H-3Glc-H_2O]$^-$, 457.3698[M-H-4Glc]$^-$	Dehydrated-protopanaxatriol+4Glc
R161	14.83	$C_{42}H_{68}O_{15}$	811.4486	0.74	649.3958[M-H-Glc]$^-$, 473.3633[M-H-Glc-Glu A]$^-$	Dehydrogenated-protopanaxatriol+Glc+Glu A
R162	14.88	$C_{50}H_{84}O_{21}$	1019.5433 ^{b}	0.59	931.5258[M-H-Ac]$^-$, 637.4310[M-H-Ac-Xyl-Glc]$^-$, 475.3778[M-H-Ac-Xyl-2Glc]$^-$	Acetyl-notoginsenoside R_1
R163	14.95	$C_{50}H_{84}O_{20}$	1003.5495	1.69	961.5333[M-H-Ac]$^-$, 799.4811[M-H-Ac-Glc]$^-$, 637.4338[M-H-Ac-2Glc]$^-$, 475.3813[M-H-Ac-3Glc]$^-$	Acetyl ginsenoside Re_1/ Acetyl ginsenoside Re_2/ Acetyl ginsenoside Re_3/ Acetyl ginsenoside 20-Gluco-ginsenoside Rf/ Acetyl notoginsenoside N
R164	14.96	$C_{58}H_{98}O_{27}$	1225.6237	1.63	1093.5796[M-H-Xyl]$^-$, 961.5352[M-H-Xyl-Ara]$^-$, 799.4846[M-H-Xyl-Ara-Glc]$^-$, 781.4697[M-H-Xyl-Ara-Glc-H_2O]$^-$, 637.4341[M-H-Xyl-Ara-2Glc]$^-$, 619.4268[M-H-Xyl-Ara-2Glc-H_2O]$^-$, 475.3808[M-H-Xyl-Ara-3Glc]$^-$	Protopanaxatriol+3Glc+Xyl+Ara
R165	14.97	$C_{42}H_{74}O_{15}$	863.4991 ^{b}	-1.51	817.4826[M-H]$^-$, 655.4399[M-H-Glc]$^-$,	Double bond hydrated-protopana

					493.3890[M-H-2Glc]$^-$	xatriol+2Glc
R166	15.06	$C_{49}H_{82}O_{20}$	989.5315	-0.61	947.5269[M-H-Ac]$^-$, 785.4678[M-H-Ac-Glc]$^-$, 767.4631[M-H-Ac-Glc-H$_2$O]$^-$, 623.4160[M-H-Ac-2Glc]$^-$, 461.3662[M-H-Ac-3Glc]$^-$	Dihydrogenated-protopanaxadiol+2Glc +Acetyl Glc
R167	15.08	$C_{51}H_{84}O_{22}$	1047.5371	-0.48	961.5437[M-H-Malonyl]$^-$, 799.4800[M-H-Malonyl-Glc]$^-$, 637.4400[M-H-Malonyl-2Glc]$^-$, 475.3819[M-H-Malonyl-3Glc]$^-$	Malonyl-notoginsenoside N isomer
R168	15.11	$C_{46}H_{76}O_{16}$	883.5042	-1.47	637.4325[M-H-2Ac-Glc]$^-$, 619.4222[M-H-2Ac-Glc-H$_2$O]$^-$, 475.3806[M-H-2Ac-2Glc]$^-$	Protopanaxatriol+Diacetyl+2Glc
R169	15.11	$C_{60}H_{102}O_{27}$	1253.6544	1.12	1091.6016[M-H-Glc]$^-$, 1073.5828[M-H-Glc-H$_2$O]$^-$, 929.5506[M-H-2Glc]$^-$, 911.5439[M-H-2Glc-H$_2$O]$^-$, 767.4948[M-H-3Glc]$^-$, 749.4890[M-H-3Glc-H$_2$O]$^-$, 605.4434[M-H-4Glc]$^-$, 443.3906[M-H-5Glc]$^-$	Deoxidated-protopanaxadiol+5Glc
R170	15.22	$C_{42}H_{72}O_{15}$	815.4797	0.49	653.4265[M-H-2Glc]$^-$, 491.3747[M-H-2Glc]$^-$	Ginsenoside Re$_5$ isomer/Ginsenjilinol isomer
R171	15.27	$C_{50}H_{84}O_{19}$	987.5527	-0.20	945.5394[M-H-Ac]$^-$, 783.4873[M-H-Ac-Glc]$^-$, 637.4286[M-H-Ac-Glc-Rha]$^-$, 475.3809[M-H-Ac-2Glc-Rha]$^-$	Acetyl-ginsenoside Re

R172	15.32	$C_{42}H_{72}O_{14}$	799.4854	1.25	637.4328[M-H-Glc]$^-$, 491.3747[M-H-Glc-Rha]$^-$	Oxidated-protopanaxatriol+Glc+Rha
R173	15.35	$C_{52}H_{84}O_{23}$	1075.5315	-0.93	961.5285[M-H-(*E*)-but-2-enoyl]$^-$, 799.4877[M-H-(*E*)-but-2-enoyl-Glc]$^-$, 637.4316[M-H-(*E*)-but-2-enoyl-2Glc]$^-$, 475.3819[M-H-(*E*)-but-2-enoyl-3Glc]$^-$	(*E*)-But-2-enoyl-ginsenoside Re$_1$/(*E*)-But-2-enoyl ginsenoside Re$_2$/(*E*)-But-2-enoyl ginsenoside Re$_3$/(*E*)-But-2-enoyl ginsenoside 20-Gluco-ginsenoside Rf/(*E*)-But-2-enoyl notoginsenoside N
R174	15.46	$C_{48}H_{82}O_{19}$	961.5378	0.62	799.4857[M-H-Glc]$^-$, 637.4321[M-H-2Glc]$^-$, 475.3797[M-H-3Glc]$^-$	Vina-ginsenoside R$_4$ isomer
R175	15.53	$C_{50}H_{84}O_{19}$	987.5520	-0.91	945.5396[M-H-Ac]$^-$, 783.4919[M-H-Ac-Glc]$^-$, 637.4324[M-H-Ac-Glc-Rha]$^-$, 475.3675[M-H-Ac-2Glc-Rha]$^-$	Acetyl-ginsenoside Re
R176	15.58	$C_{56}H_{92}O_{26}$	1179.5796	-0.17	1093.5802[M-H-Malonyl]$^-$, 961.5504[M-H-Malonyl-Ara/Xyl]$^-$, 799.4852[M-H-Malonyl-Ara/Xyl-Glc]$^-$, 637.4352[M-H-Malonyl-Ara/Xyl-2Glc]$^-$, 475.3770[M-H-Malonyl-Ara/Xyl-3Glc]$^-$	Protopanaxatriol+Ara/Xyl+2Glc+Malonyl Glc
R177	15.63	$C_{48}H_{82}O_{17}$	975.5547 ^{b}	1.85	929.5592[M-H]$^-$, 767.4908[M-H-Glc]$^-$, 621.4373[M-H-Glc-Rha]$^-$, 475.3784[M-H-Glc-2Rha]$^-$	Protopanaxatriol+Glc+2Rha
R178	15.66	$C_{58}H_{98}O_{27}$	1225.6140	1.88	1093.5677[M-H-Xyl]$^-$, 961.5416[M-H-Xyl-Ara]$^-$,	Protopanaxatriol+3Glc+Xyl+Ara

					799.4874 [M-H-Xyl-Ara-Glc]$^-$, 781.4717[M-H-Xyl-Ara-Glc-H_2O]$^-$, 637.4327[M-H-Xyl-Ara-2Glc]$^-$, 619.4221[M-H-Xyl-Ara-2Glc-H_2O]$^-$, 475.3810[M-H-Xyl-Ara-3Glc]$^-$	
R179	15.70	$C_{61}H_{98}O_{30}$	1309.6077	0.92	1223.6089[M-H-Malonyl]$^-$, 1091.5605[M-H-Malonyl-Xyl]$^-$, 959.5303[M-H-Malonyl-Xyl-Ara]$^-$, 797.4695[M-H-Malonyl-Xyl-Ara-Glc]$^-$, 779.4562[M-H-Malonyl-Xyl-Ara-Glc-H_2O]$^-$, 635.4097[M-H-Malonyl-Xyl-Ara-2Glc]$^-$, 617.4057[M-H-Malonyl-Xyl-Ara-2Glc-H_2O]$^-$, 473.3648[M-H-Malonyl-Xyl-Ara-3Glc]$^-$	Dehydrogenated-protopanaxatriol+2Glc +Malonyl Glc+Xyl+Ara
R180	15.72	$C_{42}H_{72}O_{14}$	799.4854	1.25	637.4229[M-H-Glc]$^-$, 491.3701[M-H-Glc-Rha]$^-$	Oxidated-protopanaxatriol+Glc+Rha
R181	15.76	$C_{64}H_{108}O_{31}$	1371.6781	-1.09	1239.6310[M-H-Xyl]$^-$, 1107.5912[M-H-Xyl-Ara]$^-$, 945.5331[M-H-Xyl-Ara-Glc]$^-$, 783.4913[M-H-Xyl-Ara-2Glc]$^-$, 621.4344[M-H-Xyl-Ara-3Glc]$^-$, 459.3840[M-H-Xyl-Ara-4Glc]$^-$	Protopanaxadiol+Xyl+Ara+4Glc
R182	15.78	$C_{46}H_{76}O_{16}$	883.5063	0.91	637.4381[M-H-2Ac-Glc]$^-$, 619.4207[M-H-2Ac-Glc-H_2O]$^-$, 475.3790[M-H-2Ac-2Glc]$^-$	Protopanaxatriol+Diacetyl+2Glc

R183	15.86	$C_{57}H_{92}O_{27}$	1207.5765	1.57	1121.5773[M-H-Malonyl]$^-$, 797.4738[M-H-Malonyl-2Glc]$^-$, 779.4620[M-H-Malonyl-2Glc-H_2O]$^-$, 635.4109[M-H-Malonyl-3Glc]$^-$, 617.4037[M-H-Malonyl-3Glc-H_2O]$^-$, 473.3679[M-H-Malonyl-4Glc]$^-$	Dehydrogenated-protopanaxatriol+3Glc +Malonyl Glc
R184	15.89	$C_{60}H_{102}O_{28}$	1269.6487	0.63	1107.5951[M-H-Glc]$^-$, 945.5402[M-H-2Glc]$^-$, 783.4882[M-H-3Glc]$^-$, 621.4368[M-H-4Glc]$^-$, 459.3851[M-H-5Glc]$^-$	Protopanaxadiol+5 Glc
R185	15.96	$C_{42}H_{72}O_{13}$	783.4890	-0.64	637.4326[M-H-Rha]$^-$, 475.3784[M-H-Rha-Glc]$^-$	Ginsenoside Rg_2 isomer
R186	16.10	$C_{48}H_{80}O_{19}$	1005.5276 [b]	0.60	959.5185[M-H]$^-$, 797.4698[M-H-Glc]$^-$, 779.4591[M-H-Glc-H_2O]$^-$, 635.4156[M-H-2Glc]$^-$, 617.4064[M-H-2Glc-H_2O]$^-$, 473.3707[M-H-3Glc]$^-$ 455.3529[M-H-3Glc-H_2O]$^-$	Dehydrogenated-protopanaxatriol+3Glc
R187	16.11	$C_{56}H_{90}O_{26}$	1177.5656	1.19	1091.5630[M-H-Malonyl]$^-$, 959.5215[M-H-Malonyl-Xyl/Ara]$^-$, 797.4700[M-H-Malonyl-Xyl/Ara-Glc]$^-$, 779.4589[M-H-Malonyl-Xyl/Ara-Glc-H_2O]$^-$, 635.4180[M-H-Malonyl-Xyl/Ara-2Glc]$^-$, 617.4060[M-H-Malonyl-Xyl/Ara-2Glc-	Dehydrogenated-protopanaxatriol+Malonyl Glc+2Glc+Xyl/Ara

					H_2O]⁻, 473.3636[M-H-Malonyl-Xyl/Ara-3Glc]⁻	
R188	16.14	$C_{51}H_{84}O_{22}$	1047.5382	0.57	961.5370[M-H-Malonyl]⁻, 799.4870[M-H-Malonyl-Glc]⁻, 637.4325[M-H-Malonyl-2Glc]⁻, 475.3807[M-H-Malonyl-3Glc]⁻	Malonyl-notoginsenoside N
R189	16.19	$C_{48}H_{82}O_{19}$	961.5376	0.42	799.4960[M-H-Glc]⁻, 637.4346[M-H-2Glc]⁻, 475.3803[M-H-3Glc]⁻	Vina-ginsenoside R_4 isomer
R190	16.25	$C_{54}H_{92}O_{23}$	1107.5951	0.00	945.5400[M-H-Glc]⁻, 783.4893[M-H-2Glc]⁻, 621.4345[M-H-3Glc]⁻, 459.3834[M-H-4Glc]⁻	Ginsenoside Rb_1 isomer
R191	16.25	$C_{50}H_{84}O_{20}$	1003.5493	1.49	961.5358[M-H-Ac]⁻, 799.4869[M-H-Ac-Glc]⁻, 637.4338[M-H-Ac-2Glc]⁻, 475.3797[M-H-Ac-3Glc]⁻	Acetyl ginsenoside Re_1/ Acetyl ginsenoside Re_2/ Acetyl ginsenoside Re_3/ Acetyl ginsenoside 20-Gluco-ginsenoside Rf/ Acetyl notoginsenoside N
R192	16.26	$C_{42}H_{72}O_{14}$	799.4854	1.25	637.4323[M-H-Glc]⁻, 475.3793[M-H-2Glc]⁻	Ginsenoside Rf isomer
R193	16.30	$C_{42}H_{72}O_{15}$	815.4791	-0.25	815.4696[M-H]⁻， 653.4249[M-H-2Glc]⁻， 491.3750[M-H-2Glc]⁻	Ginsenoside Re_5 isomer/Ginsenjilinol isomer
R194	16.31	$C_{42}H_{70}O_{14}$	843.4754 [b]	1.42	635.4182[M-H-Glc]⁻, 473.3656[M-H-2Glc]⁻	Dehydrogenated-protopanaxatriol+2Glc
R195	16.36	$C_{59}H_{100}O_{27}$	1239.6388	1.13	1107.5933[M-H-Glc]⁻, 945.5407[M-H-Glc-Xyl]⁻, 783.4976[M-H-2Glc-Xyl]⁻,	Notoginsenoside R_4 isomer/Ginsenoside Ra_3 isomer

					621.4368[M-H-3Glc-Xyl]-, 459.3808[M-H-4Glc-Xyl]-	
R196	16.39	$C_{46}H_{76}O_{16}$	883.5073	2.04	637.4356[M-H-2Ac-Glc]-, 619.4208[M-H-2Ac-Glc-H_2O]-, 475.3770[M-H-2Ac-2Glc]-	Protopanaxatriol+Diacetyl+2Glc
R197	16.41	$C_{46}H_{76}O_{16}$	883.5072	1.92	637.4335[M-H-2Ac-Glc]-, 619.4194[M-H-2Ac-Glc-H_2O]-, 475.3767[M-H-2Ac-2Glc]-	Protopanaxatriol+Diacetyl+2Glc
R198	16.42	$C_{69}H_{116}O_{35}$	1549.7268 [b]	-0.39	1503.7040[M-H]-, 1341.6509[M-H-Glc]-, 1209.6332[M-H-Glc-Xyl]-, 1077.5833[M-H-Glc-Xyl-Ara]-, 945.5369[M-H-Glc-Xyl-Ara-Xyl/Ara]-, 783.4903[M-H-2Glc-Xyl-Ara-Xyl/Ara]-, 621.4357[M-H-3Glc-Xyl-Ara-Xyl/Ara]-, 459.3835[M-H-4Glc-Xyl-Ara-Xyl/Ara]-	Protopanaxadiol+Xyl+Ara+Xyl/Ara+4Glc
R199	16.49	$C_{50}H_{82}O_{20}$	1001.5327	0.60	797.4761[M-H-Ac-Glc]-, 779.4597[M-H-Ac-Glc-H_2O]-, 617.4083[M-H-Ac-2Glc-H_2O]-, 455.3528[M-H-Ac-3Glc-H_2O]-	Dehydrogenated-protopanaxatriol+2Glc +Acetyl Glc
R200	16.50	$C_{42}H_{72}O_{14}$	799.4844	0.00	637.4310[M-H-Glc]-, 475.3797[M-H-2Glc]-	Ginsenoside Rf [a]
R201	16.55	$C_{59}H_{100}O_{27}$	1239.6376	0.16	1107.5955[M-H-Glc]-, 945.5423[M-H-Glc-Xyl]-, 783.4895[M-H-2Glc-Xyl]-, 621.4370[M-H-3Glc-Xyl]-, 459.3840[M-H-4Glc-Xyl]-	Notoginsenoside R_4 [a]
R202	16.65	$C_{56}H_{92}O_{26}$	1179.5806	0.68	1093.5795[M-H-Malonyl]-, 961.5365[M-H-Malonyl-Ara/Xyl]-,	Protopanaxatriol+ Ara/Xyl+2Glc+ Malonyl Glc

					799.4838[M-H-Malonyl-Ara/Xyl-Glc]$^-$, 637.4296[M-H-Malonyl-Ara/Xyl-2Glc]$^-$, 475.3789[M-H-Malonyl-Ara/Xyl-3Glc]$^-$	
R203	16.68	$C_{51}H_{84}O_{22}$	1047.5382	0.57	961.5382[M-H-Malonyl]$^-$, 799.4823[M-H-Malonyl-Glc]$^-$, 637.4295 [M-H-Malonyl-2Glc]$^-$, 475.3821[M-H-Malonyl-3Glc]$^-$	Malonyl-vina-ginsenoside R_4
R204	16.69	$C_{61}H_{102}O_{28}$	1281.6475	-0.31	1239.6373[M-H-Ac]$^-$, 1107.5952[M-H-Ac-Xyl/Ara]$^-$, 945.5424[M-H-Ac-Xyl/Ara-Glc]$^-$, 783.4902[M-H-Ac-Xyl/Ara-2Glc]$^-$, 621.4371[M-H-Ac-Xyl/Ara-3Glc]$^-$, 459.3857[M-H-Ac-Xyl/Ara-4Glc]$^-$	Acetyl-ginsenoside Ra_3/Acetyl-notoginsenoside R_4
R205	16.71	$C_{57}H_{94}O_{26}$	1193.5942	-1.09	1107.5931[M-H-Malonyl]$^-$, 945.5436[M-H-Malonyl-Glc]$^-$, 783.4905[M-H-Malonyl-2Glc]$^-$, 621.4352[M-H-Malonyl-3Glc]$^-$, 459.3823[M-H-Malonyl-4Glc]$^-$	Malonyl-ginsenoside Rb_1 isomer
R206	16.74	$C_{62}H_{102}O_{30}$	1325.6384	0.45	1239.6379[M-H-Malonyl]$^-$, 1107.5953[M-H-Malonyl-Xyl]$^-$, 945.5336[M-H-Malonyl-Xyl-Glc]$^-$, 783.4839[M-H-Malonyl-Xyl-2Glc]$^-$, 621.4380[M-H-Malonyl-Xyl-3Glc]$^-$, 459.3823[M-H-Malonyl-Xyl-4Glc]$^-$	Malonyl-notoginsenoside R_4 isomer/Malonyl-ginsenoside Ra_3 isomer
R207	16.76	$C_{48}H_{82}O_{19}$	961.5383	1.14	799.4841[M-H-Glc]$^-$, 637.4267[M-H-2Glc]$^-$, 475.3792[M-H-3Glc]$^-$	Vina-ginsenoside R_4 isomer
R208	16.78	$C_{64}H_{108}O_{31}$	1371.6790	-0.44	1239.6427[M-H-Xyl]$^-$, 1107.5883[M-H-Xyl-Ara]$^-$,	Protopanaxadiol+Xyl+Ara+4Glc

					945.5387[M-H-Xyl-Ara-Glc]-, 783.4902[M-H-Xyl-Ara-2Glc]-, 621.4384[M-H-Xyl-Ara-3Glc]-, 459.3811[M-H-Xyl-Ara-4Glc]-	
R209	16.83	$C_{61}H_{100}O_{29}$	1295.6267	-0.39	1209.6281[M-H-Malonyl] -, 1077.5908 [M-H-Malonyl-Xyl] -, 945.5458[M-H-Malonyl-Xyl-Ara] -, 783.4890[M-H-Malonyl-Xyl-Ara-Glc] -, 621.4402[M-H-Malonyl-Xyl-Ara-2Glc] -, 459.3851[M-H-Malonyl-Xyl-Ara-3Glc] -	Malonyl-ginsenoside Ra_1/ Malonyl-ginsenoside Ra_2
R210	16.88	$C_{42}H_{72}O_{13}$	783.4908	1.66	475.3784[M-H-Rha-Glc]-	Ginsenoside Rg_2 isomer
R211	16.88	$C_{62}H_{102}O_{30}$	1325.6356	-1.66	1239.6378[M-H-Malonyl]-, 1107.5924[M-H-Malonyl-Xyl]-, 945.5419[M-H-Malonyl-Xyl-Glc]-, 783.4904[M-H-Malonyl-Xyl-2Glc]-, 621.4343[M-H-Malonyl-Xyl-3Glc]-, 459.3821[M-H-Malonyl-Xyl-4Glc]-	Malonyl-notoginsenoside R_4
R212	16.91	$C_{61}H_{100}O_{29}$	1295.6267	-0.39	1209.6287[M-H-Malonyl] -, 1077.5913[M-H-Malonyl-Xyl] -, 945.5440[M-H-Malonyl-Xyl-Ara] -, 783.4888[M-H-Malonyl-Xyl-Ara-Glc] -, 621.4340[M-H-Malonyl-Xyl-Ara-2Glc] -, 459.3855[M-H-Malonyl-Xyl-Ara-3Glc] -	Malonyl-ginsenoside Ra_1/ Malonyl-ginsenoside Ra_2
R213	16.97	$C_{42}H_{72}O_{13}$	783.4907	1.53	475.3777[M-H-Rha-Glc]-	Ginsenoside Rg_2 isomer

R214	16.98	$C_{59}H_{100}O_{27}$	1239.6382	0.65	1107.5958[M-H-Glc]$^-$, 945.5446[M-H-Glc-Xyl]$^-$, 783.4906[M-H-2Glc-Xyl]$^-$, 621.4390[M-H-3Glc-Xyl]$^-$, 459.3846[M-H-4Glc-Xyl]$^-$	Notoginsenoside R_4 isomer/Ginsenoside Ra_3 isomer
R215	17.08	$C_{61}H_{102}O_{28}$	1281.6471	-0.62	1239.6389[M-H-Ac]$^-$, 1107.5958[M-H-Ac-Xyl/Ara]$^-$, 945.5429[M-H-Ac-Xyl/Ara-Glc]$^-$, 783.4897[M-H-Ac-Xyl/Ara-2Glc]$^-$, 621.4372[M-H-Ac-Xyl/Ara-3Glc]$^-$, 459.3835[M-H-Ac-Xyl/Ara-4Glc]$^-$	Acetyl-ginsenoside Ra_3/Acetyl-notoginsenoside R_4
R216	17.17	$C_{41}H_{70}O_{13}$	769.4741	0.39	637.4324[M-H-Xyl]$^-$, 475.3792[M-H-Xyl-Glc]$^-$	Notoginsenoside R_2 [a]
R217	17.18	$C_{51}H_{84}O_{22}$	1047.5367	-0.86	961.5367[M-H-Malonyl]$^-$, 799.4769[M-H-Malonyl-Glc]$^-$, 637.4283[M-H-Malonyl-2Glc]$^-$, 475.3801[M-H-Malonyl-3Glc]$^-$	Malonyl-vina-ginsenoside R_4 isomer
R218	17.18	$C_{65}H_{108}O_{31}$	1383.6803	0.51	1341.6703[M-H-Ac]$^-$, 1323.6552[M-H-Ac-H_2O]$^-$, 1209.6250[M-H-Ac-Xyl]$^-$, 1191.6195[M-H-Ac-Xyl-H_2O]$^-$, 1077.5776[M-H-Ac-Xyl-Ara]$^-$, 1059.5737[M-H-Ac-Xyl-Ara-H_2O]$^-$, 945.5395[M-H-Ac-Xyl-Ara-Xyl/Ara]$^-$, 783.48555[M-H-Ac-Xyl-Ara-Xyl/Ara-Glc]$^-$, 621.4359[M-H-Ac-Xyl-Ara-Xyl/Ara-2Glc]$^-$, 459.3844[M-H-Ac-Xyl-Ara-Xyl/Ara-3G	Protopanaxadiol+Xyl+Ara+Xyl/Ara+3Glc+Acetyl

					lc]$^-$	
R219	17.21	$C_{60}H_{102}O_{28}$	1269.6471	-0.63	1107.5922[M-H-Glc]$^-$, 945.5416[M-H-2Glc]$^-$, 783.4902[M-H-3Glc]$^-$, 621.4380[M-H-4Glc]$^-$, 459.3835[M-H-5Glc]$^-$	Protopanaxadiol+5 Glc
R220	17.21	$C_{56}H_{92}O_{26}$	1179.5784	-1.19	1093.5788[M-H-Malonyl]$^-$, 961.5355[M-H-Malonyl-Ara/Xyl]$^-$, 799.4799[M-H-Malonyl-Ara/Xyl-Glc]$^-$, 637.4306[M-H-Malonyl-Ara/Xyl-2Glc]$^-$, 475.3744[M-H-Malonyl-Ara/Xyl-3Glc]$^-$	Protopanaxatriol+ Ara/Xyl+2Glc+ Malonyl Glc
R221	17.21	$C_{53}H_{84}O_{23}$	1087.5337	1.10	793.4382[M-H-Glc-Xyl/Ara]$^-$, 731.4387[M-H-Glc-Xyl/Ara-CO_2-H_2O]$^-$, 613.3707[M-H-Glc-Xyl/Ara-Glc-H_2O]$^-$, 569.3850[M-H-Glc-Xyl/Ara-Glc-H_2O-CO_2]$^-$, 455.3531[M-H-2Glc-Xyl/Ara-Glu A]$^-$	Oleanolic aglycone+2Glc+Xyl/ Ara+Glu A
R222	17.23	$C_{44}H_{74}O_{15}$	841.4945	0.91	799.4879[M-H-Ac]$^-$, 637.4313[M-H-Ac-Glc]$^-$, 475.3795[M-H-Ac-2Glc]$^-$	Yesanchinoside D
R223	17.25	$C_{57}H_{94}O_{26}$	1193.5932	-1.93	1107.5909[M-H-Malonyl]$^-$, 945.5381[M-H-Malonyl-Glc]$^-$, 783.4945[M-H-Malonyl-2Glc]$^-$, 621.4410[M-H-Malonyl-3Glc]$^-$, 459.3853[M-H-Malonyl-4Glc]$^-$	Malonyl-ginsenoside Rb$_1$ isomer
R224	17.27	$C_{42}H_{70}O_{15}$	859.4685 [b]	-0.70	813.4393[M-H]$^-$, 769.4633[M-H-CO_2]$^-$, 637.4297[M-H-Glu A]$^-$, 475.3783[M-H-Glu A-Glc]$^-$	Protopanaxatriol+Glu A+Glc
R225	17.28	$C_{59}H_{100}O_{27}$	1239.6375	0.08	1107.5924[M-H-Glc]$^-$,	Notoginsenoside R$_4$

					945.5419[M-H-Glc-Xyl]$^-$, 783.4904[M-H-2Glc-Xyl]$^-$, 621.4343[M-H-3Glc-Xyl]$^-$, 459.3804[M-H-4Glc-Xyl]$^-$	isomer/Ginsenoside Ra_3 isomer
R226	17.28	$C_{43}H_{68}O_{14}$	807.4534	0.37	765.4423[M-H-Ac]$^-$, 603.3893[M-H-Ac-Glc]$^-$, 441.3388[M-H-Ac-2Glc]$^-$	Dehydrated-protopanaxadiol+Acetyl Glc+Glc
R227	17.33	$C_{62}H_{102}O_{30}$	1325.6377	-0.08	1239.6313[M-H-Malonyl]$^-$, 1107.5941[M-H-Malonyl-Xyl]$^-$, 945.5395[M-H-Malonyl-Xyl-Glc]$^-$, 783.4912[M-H-Malonyl-Xyl-2Glc]$^-$, 621.4358[M-H-Malonyl-Xyl-3Glc]$^-$, 459.3817[M-H-Malonyl-Xyl-4Glc]$^-$	Malonyl-notoginsenoside R_4 isomer/Malonyl-ginsenoside Ra_3 isomer
R228	17.34	$C_{45}H_{74}O_{17}$	885.4843	-0.56	637.4288[M-H-Malonyl-Glc]$^-$, 475.3795[M-H-Malonyl-2Glc]$^-$	Malonyl-ginsenoside Ia
R229	17.34	$C_{61}H_{102}O_{28}$	1281.6484	0.39	1239.6371[M-H-Ac]$^-$, 1107.5908[M-H-Ac-Xyl/Ara]$^-$, 945.5460[M-H-Ac-Xyl/Ara-Glc]$^-$, 783.4949[M-H-Ac-Xyl/Ara-2Glc]$^-$, 621.4390[M-H-Ac-Xyl/Ara-3Glc]$^-$, 459.3823[M-H-Ac-Xyl/Ara-4Glc]$^-$	Acetyl-ginsenoside Ra_3/Acetyl-notoginsenoside R_4
R230	17.38	$C_{41}H_{70}O_{13}$	769.4743	0.65	637.4335[M-H-Ara]$^-$, 475.3577[M-H-Ara-Glc]$^-$	Ginsenoside F_3/Ginsenoside F_5
R231	17.38	$C_{54}H_{90}O_{23}$	1105.5796	0.09	943.5290[M-H-Glc]$^-$, 781.4714[M-H-2Glc]$^-$, 619.4185[M-H-3Glc]$^-$, 457.3685[M-H-4Glc]$^-$	Dehydrated-protopanaxatriol+4Glc
R232	17.40	$C_{42}H_{72}O_{14}$	799.4845	0.13	637.4327[M-H-Glc]$^-$,	Ginsenoside Rf

					475.3800[M-H-2Glc]$^-$	isomer
R233	17.41	$C_{60}H_{100}O_{27}$	1251.6364	-0.80	1077.5898[M-H-Ac-Xyl]$^-$, 945.5436[M-H-Ac-Xyl-ara(p)]$^-$, 783.4938[M-H-Ac-Xyl- ara(p)-Glc]$^-$, 621.4387[M-H-Ac-Xyl- ara(p)-2Glc]$^-$, 459.3845[M-H-Ac-Xyl- ara(p)-3Glc]$^-$	Ginsenoside Ra_5 isomer
R234	17.42	$C_{58}H_{98}O_{26}$	1209.6272	0.33	1077.5869[M-H-Xyl]$^-$, 945.5433[M-H-Xyl-Ara]$^-$, 783.4961[M-H-Xyl-Ara-Glc]$^-$, 621.4344[M-H-Xyl-Ara-2Glc]$^-$, 459.3845[M-H-Xyl-Ara-3Glc]$^-$	Ginsenoside Ra_1 isomer/Ginsenoside Ra_2 isomer
R235	17.45	$C_{56}H_{94}O_{24}$	1149.6029	-2.44	1107.5918[M-H-Ac]$^-$, 945.5482 [M-H-Ac-Glc]$^-$, 783.4918[M-H-Ac-2Glc]$^-$, 621.4401[M-H-Ac-3Glc]$^-$, 459.3868[M-H-Ac-4Glc]$^-$	Quinquenoside R_1 isomer
R236	17.48	$C_{59}H_{100}O_{27}$	1239.6377	0.24	1107.5844[M-H-Glc]$^-$, 945.5328[M-H-Glc-Xyl]$^-$, 783.4883[M-H-2Glc-Xyl]$^-$, 621.4341[M-H-3Glc-Xyl]$^-$, 459.3811[M-H-4Glc-Xyl]$^-$	Notoginsenoside R_4 isomer/Ginsenoside Ra_3 isomer
R237	17.49	$C_{41}H_{70}O_{13}$	769.4753	1.95	637.4319[M-H-Ara]$^-$, 475.3796[M-H-Ara-Glc]$^-$	Ginsenoside F_3/Ginsenoside F_5
R238	17.50	$C_{59}H_{98}O_{27}$	1237.6221	0.32	1105.5785[M-H-Xyl/Ara]$^-$, 943.5281[M-H-Xyl/Ara-Glc]$^-$, 781.4758[M-H-Xyl/Ara-2Glc]$^-$, 763.4532[M-H-Xyl/Ara-2Glc-H_2O]$^-$, 619.4229[M-H-Xyl/Ara-3Glc]$^-$, 457.3721[M-H-Xyl/Ara-4Glc]$^-$	Dehydrated-protopanaxatriol+Xyl/Ara +4Glc
R239	17.52	$C_{44}H_{74}O_{15}$	841.4941	0.39	799.4834[M-H-Ac]$^-$, 637.4296[M-H-Ac-Glc]$^-$,	Yesanchinoside D isomer

					475.3789[M-H-Ac-2Glc]$^-$	
R240	17.52	$C_{48}H_{76}O_{19}$	955.4905	0.21	793.4385[M-H-Glc]$^-$, 731.4379[M-H-Glc-CO_2-H_2O]$^-$, 569.3853[M-H-2Glc-H_2O-CO_2]$^-$, 455.3515[M-H-2Glc-Glu A]$^-$	Ginsenoside Ro isomer
R241	17.55	$C_{50}H_{82}O_{20}$	1001.5337	1.60	959.5232[M-H-Ac]$^-$, 797.4665[M-H-Ac-Glc]$^-$, 635.4185[M-H-Ac-2Glc]$^-$, 473.3651[M-H-Ac-3Glc]$^-$	Dehydrogenated-protopanaxatriol+2Glc +Acetyl Glc
R242	17.59	$C_{56}H_{94}O_{24}$	1149.6071	1.22	945.5449 [M-H-Ac-Glc]$^-$, 783.4938[M-H-Ac-2Glc]$^-$, 621.4384[M-H-Ac-3Glc]$^-$, 459.3892[M-H-Ac-4Glc]$^-$	Quinquenoside R_1 isomer
R243	17.60	$C_{42}H_{72}O_{14}$	799.4840	-0.50	637.4350[M-H-Glc]$^-$, 475.3799[M-H-2Glc]$^-$	Ginsenoside Rf isomer
R244	17.61	$C_{62}H_{102}O_{30}$	1325.6378	0.00	1239.6312[M-H-Malonyl]$^-$, 1107.5948[M-H-Malonyl-Xyl]$^-$, 945.5328[M-H-Malonyl-Xyl-Glc]$^-$, 783.4883[M-H-Malonyl-Xyl-2Glc]$^-$, 621.4341[M-H-Malonyl-Xyl-3Glc]$^-$, 459.3831[M-H-Malonyl-Xyl-4Glc]$^-$	Malonyl-notoginsenoside R_4 isomer/Malonyl-ginsenoside Ra_3 isomer
R245	17.62	$C_{60}H_{102}O_{28}$	1269.6476	-0.24	1107.6014[M-H-Glc]$^-$, 945.5500[M-H-2Glc]$^-$, 783.4929[M-H-3Glc]$^-$, 621.4399[M-H-4Glc]$^-$, 459.3786[M-H-5Glc]$^-$	Protopanaxadiol+5 Glc
R246	17.64	$C_{41}H_{68}O_{13}$	767.4588	0.78	635.4172[M-H-Xyl/Ara]$^-$, 473.3636[M-H-Xyl/Ara-Glc]$^-$	Dehydrogenated-protopanaxatriol+ Xyl/Ara+Glc
R247	17.68	$C_{51}H_{84}O_{22}$	1047.5367	-0.86	799.4898[M-H-Malonyl-Glc]$^-$,	Malonyl-vina-ginse

					637.4297[M-H-Malonyl-2Glc]$^-$, 475.3849 [M-H-Malonyl-3Glc]$^-$	noside R_4 isomer
R248	17.69	$C_{58}H_{98}O_{26}$	1209.6262	-0.50	1077.5844[M-H-Xyl]$^-$, 945.5457[M-H-Xyl-Ara(f)]$^-$, 783.4891[M-H-Xyl-Ara(f)-Glc]$^-$, 621.4388[M-H-Xyl-Ara(f)-2Glc]$^-$, 459.3869[M-H-Xyl-Ara(f)-3Glc]$^-$	Ginsenoside Ra_2 [a]
R249	17.70	$C_{60}H_{102}O_{28}$	1269.6465	-1.10	1107.5936[M-H-Glc]$^-$, 945.5430[M-H-2Glc]$^-$, 783.4886[M-H-3Glc]$^-$, 621.4363[M-H-4Glc]$^-$, 459.3849[M-H-5Glc]$^-$	Protopanaxadiol+5 Glc
R250	17.70	$C_{61}H_{102}O_{28}$	1281.6488	0.70	1239.6315[M-H-Ac]$^-$, 1107.5852[M-H-Ac-Xyl/Ara]$^-$, 945.5457[M-H-Ac-Xyl/Ara-Glc]$^-$, 783.4911[M-H-Ac-Xyl/Ara-2Glc]$^-$, 621.4362[M-H-Ac-Xyl/Ara-3Glc]$^-$, 459.3851[M-H-Ac-Xyl/Ara-4Glc]$^-$	Acetyl-ginsenoside Ra_3/Acetyl-notogins enoside R_4
R251	17.72	$C_{64}H_{108}O_{31}$	1371.6782	-1.02	1239.6383[M-H-Xyl]$^-$, 1107.5880[M-H-Xyl-Ara]$^-$, 945.5468[M-H-Xyl-Ara-Glc]$^-$, 783.4961[M-H-Xyl-Ara-2Glc]$^-$, 621.4357[M-H-Xyl-Ara-3Glc]$^-$, 459.3835[M-H-Xyl-Ara-4Glc]$^-$	Protopanaxadiol+X yl+Ara+4Glc
R252	17.76	$C_{61}H_{100}O_{29}$	1295.6267	-0.39	1077.5815[M-H-Malonyl-Xyl]$^-$, 945.5440[M-H-Malonyl-Xyl-Ara]$^-$, 783.4857[M-H-Malonyl-Xyl-Ara-Glc]$^-$, 621.4341[M-H-Malonyl-Xyl-Ara-2Glc]$^-$,	Malonyl-ginsenosid e Ra_1/ Malonyl-ginsenosid e Ra_2

					459.3857[M-H-Malonyl-Xyl-Ara-3Glc]$^{-}$	
R253	17.77	$C_{48}H_{76}O_{19}$	955.4910	0.73	793.4365[M-H-Glc]$^{-}$, 731.4373[M-H-Glc-CO_2-H_2O]$^{-}$, 613.3727[M-H-2Glc-H_2O]$^{-}$, 569.3859[M-H-2Glc-H_2O-CO_2]$^{-}$, 455.3513[M-H-2Glc-Glu A]$^{-}$	Ginsenoside Ro isomer
R254	17.78	$C_{61}H_{102}O_{28}$	1281.6470	-0.70	1239.6387[M-H-Ac]$^{-}$, 1107.5953[M-H-Ac-Xyl/Ara]$^{-}$, 945.5441[M-H-Ac-Xyl/Ara-Glc]$^{-}$, 783.4907[M-H-Ac-Xyl/Ara-2Glc]$^{-}$, 621.4378[M-H-Ac-Xyl/Ara-3Glc]$^{-}$, 459.3849[M-H-Ac-Xyl/Ara-4Glc]$^{-}$	Acetyl-ginsenoside Ra_3/Acetyl-notoginsenoside R_4
R255	17.81	$C_{53}H_{84}O_{23}$	1087.5334	0.83	793.4398[M-H-Glc-Xyl/Ara]$^{-}$, 731.4387[M-H-Glc-Xyl/Ara-CO_2-H_2O]$^{-}$, 613.3809[M-H-Glc-Xyl/Ara-Glc-H_2O]$^{-}$, 455.3527[M-H-2Glc-Xyl/Ara-Glu A]$^{-}$	Oleanolic aglycone+2Glc+Xyl/Ara+Glu A
R256	17.83	$C_{62}H_{102}O_{30}$	1325.6378	0.00	1239.6409[M-H-Malonyl]$^{-}$, 1107.5947[M-H-Malonyl-Xyl]$^{-}$, 945.5432[M-H-Malonyl-Xyl-Glc]$^{-}$, 783.4896[M-H-Malonyl-Xyl-2Glc]$^{-}$, 621.4371[M-H-Malonyl-Xyl-3Glc]$^{-}$, 459.3842[M-H-Malonyl-Xyl-4Glc]$^{-}$	Malonyl-notoginsenoside R_4 isomer/Malonyl-ginsenoside Ra_3 isomer
R257	17.86	$C_{59}H_{100}O_{27}$	1239.6375	0.08	1107.5947[M-H-Glc]$^{-}$, 945.5432[M-H-Glc-Xyl]$^{-}$, 783.4896[M-H-2Glc-Xyl]$^{-}$, 621.4371[M-H-3Glc-Xyl]$^{-}$, 459.3842[M-H-4Glc-Xyl]$^{-}$	Ginsenoside Ra_3 [a]
R258	17.87	$C_{58}H_{96}O_{24}$	1175.6230	1.45	945.5481[M-H-(*E*)-but-2-enoyl-Glc]$^{-}$, 783.4847[M-H-(*E*)-but-2-enoyl-2Glc]$^{-}$,	Ginsenoside Ra_6 isomer

					621.4365[M-H-(*E*)-but-2-enoyl-3Glc]-, 459.3833[M-H-(*E*)-but-2-enoyl-4Glc]-	
R259	17.90	$C_{54}H_{92}O_{23}$	1107.5953	0.18	945.5349[M-H-Glc]-, 783.4890[M-H-2Glc]-, 621.4276[M-H-3Glc]-, 459.3860[M-H-4Glc]-	Ginsenoside Rb_1 [a]
R260	17.94	$C_{52}H_{80}O_{20}$	1023.5168	0.29	955.4902[M-H-(*E*)-but-2-enoyl]-, 793.4432[M-H-(*E*)-but-2-enoyl-Glc]-, 731.4376[M-H-(*E*)-but-2-enoyl-Glc-CO 2-H2O]-, 613.3828[M-H-(*E*)-but-2-enoyl-2Glc-H2 O]-, 569.3899[M-H-(*E*)-but-2-enoyl-2Glc-H2 O-CO2]-, 455.3538[M-H-(*E*)-but-2-enoyl-2Glc-Gl u A]-	(*E*)-But-2-enoyl ginsenoside Ro
R261	17.96	$C_{42}H_{72}O_{13}$	783.4894	-0.13	637.4330[M-H-Rha]-, 475.3798[M-H-Rha-Glc]-	Ginsenoside 20(*S*)-Rg_2 [a]
R262	17.96	$C_{56}H_{92}O_{24}$	1147.5891	-0.78	1105.5797[M-H-Ac]-, 943.5302[M-H-Ac-Glc]-, 781.4748[M-H-Ac-2Glc]-, 619.4218[M-H-Ac-3Glc]-, 457.3690[M-H-Ac-4Glc]-	Dehydrated-protop anaxatriol+Acetyl Glc+3Glc
R263	17.98	$C_{36}H_{62}O_9$	637.4315	-0.16	475.3785[M-H-Glc]-	Ginsenoside 20(*S*)-Rh_1 [a]
R264	17.99	$C_{44}H_{74}O_{15}$	841.4949	1.43	799.4871[M-H-Ac]-, 637.4312[M-H-Ac-Glc]-, 475.3795[M-H-Ac-2Glc]-	Yesanchinoside D isomer
R265	17.99	$C_{48}H_{76}O_{19}$	955.4911	0.84	793.4365[M-H-Glc]-, 731.4370[M-H-Glc-CO2-H2O]-,	Ginsenoside Ro isomer

					613.3737[M-H-2Glc-H_2O]$^-$, 569.3853[M-H-2Glc-H_2O-CO_2]$^-$, 455.3526[M-H-2Glc-Glu A]$^-$	
R266	17.99	$C_{43}H_{72}O_{14}$	857.4907 [b]	0.93	769.4741[M-H-Ac]$^-$, 637.4314[M-H-Ac-Xyl/Ara]$^-$, 475.3787[M-H-Ac-Xyl/Ara-Glc]$^-$	Protopanaxatriol+ Xyl/Ara+Acetyl Glc
R267	18.00	$C_{56}H_{92}O_{25}$	1163.5829	-1.72	1077.5968[M-H-Malonyl]$^-$, 945.5432[M-H-Malonyl-Ara(f)]$^-$, 783.4899[M-H-Malonyl-Ara(f)-Glc]$^-$, 621.4388[M-H-Malonyl-Ara(f)-2Glc]$^-$, 459.3859[M-H-Malonyl-Ara(f)-3Glc]$^-$	Malonyl-ginsenoside Rc/ Malonyl-ginsenoside Rb_2/ Malonyl-ginsenoside Rb_3
R268	18.01	$C_{61}H_{100}O_{29}$	1295.6254	-1.39	1209.62268[M-H-Malonyl]$^-$, 1077.5892[M-H-Malonyl-Xyl]$^-$, 945.5394[M-H-Malonyl-Xyl-Ara]$^-$, 783.4913[M-H-Malonyl-Xyl-Ara-Glc]$^-$, 621.4399[M-H-Malonyl-Xyl-Ara-2Glc]$^-$, 459.3857[M-H-Malonyl-Xyl-Ara-3Glc]$^-$	Malonyl-ginsenoside Ra_1/ Malonyl-ginsenoside Ra_2
R269	18.03	$C_{60}H_{96}O_{29}$	1279.5951	-0.63	1107.5962[M-H-2Malonyl]$^-$, 945.5468[M-H-2Malonyl-Glc]$^-$, 783.4888[M-H-2Malonyl-2Glc]$^-$, 621.4373[M-H-2Malonyl-3Glc]$^-$, 459.3826[M-H-2Malonyl-4Glc]$^-$	Di-malonyl-ginsenoside Rb_1 isomer
R270	18.03	$C_{56}H_{92}O_{24}$	1147.5884	-1.39	1105.58367[M-H-Ac]$^-$, 943.5201[M-H-Ac-Glc]$^-$, 781.4744[M-H-Ac-2Glc]$^-$, 619.4216[M-H-Ac-3Glc]$^-$, 457.3681[M-H-Ac-4Glc]$^-$	Dehydrated-protopanaxatriol+Acetyl Glc+3Glc
R271	18.04	$C_{58}H_{98}O_{26}$	1209.6265	-0.25	1077.5844[M-H-Xyl]$^-$,	Ginsenoside Ra_1

					945.5478[M-H-Xyl-Ara]-, 783.4885[M-H-Xyl-Ara-Glc]-, 621.4399[M-H-Xyl-Ara-2Glc]-, 459.3831[M-H-Xyl-Ara-3Glc]-	isomer/Ginsenoside Ra_2 isomer
R271	18.08	$C_{59}H_{100}O_{27}$	1239.6378	0.32	1107.5983[M-H-Glc]-, 945.5456[M-H-Glc-Xyl]-, 783.4915[M-H-2Glc-Xyl]-, 621.4380[M-H-3Glc-Xyl]-, 459.3831[M-H-4Glc-Xyl]-	Notoginsenoside R_4 isomer/Ginsenoside Ra_3 isomer
R273	18.13	$C_{54}H_{92}O_{23}$	1107.5959	0..72	945.5441[M-H-Glc]-, 783.4879[M-H-2Glc]-, 621.4361[M-H-3Glc]-, 459.3828[M-H-4Glc]-	Ginsenoside Rb_1 isomer
R274	18.15	$C_{56}H_{92}O_{25}$	1163.5840	-0.77	1077.6033[M-H-Malonyl]-, 945.5459[M-H-Malonyl-Ara(f)]-, 783.4794[M-H-Malonyl-Ara(f)-Glc]-, 621.4474[M-H-Malonyl-Ara(f)-2Glc]-, 459.3839[M-H-Malonyl-Ara(f)-3Glc]-	Malonyl-ginsenoside Rc/ Malonyl-ginsenoside Rb_2/ Malonyl-ginsenoside Rb_3
R275	18.15	$C_{51}H_{84}O_{22}$	1047.5362	-1.34	961.5318[M-H-Malonyl]-, 799.4782[M-H-Malonyl-Glc]-, 637.4335[M-H-Malonyl-2Glc]-, 475.3820 [M-H-Malonyl-3Glc]-	Malonyl-vina-ginsenoside R_4 isomer
R276	18.16	$C_{60}H_{100}O_{27}$	1251.6384	0.80	1077.5820[M-H-Ac-Xyl]-, 945.5406[M-H-Ac-Xyl-ara(p)]-, 783.4877[M-H-Ac-Xyl- ara(p)-Glc]-, 621.4356[M-H-Ac-Xyl- ara(p)-2Glc]-, 459.3829[M-H-Ac-Xyl- ara(p)-3Glc]-	Ginsenoside Ra_5 isomer
R277	18.18	$C_{62}H_{102}O_{30}$	1325.6371	-0.53	1239.6536[M-H-Malonyl]-, 1107.5983[M-H-Malonyl-Xyl]-, 945.5456[M-H-Malonyl-Xyl-Glc]-,	Malonyl-ginsenoside Ra_3

					783.4915[M-H-Malonyl-Xyl-2Glc]$^-$, 621.4380[M-H-Malonyl-Xyl-3Glc]$^-$, 459.3831[M-H-Malonyl-Xyl-4Glc]$^-$	
R278	18.25	$C_{60}H_{100}O_{27}$	1251.6353	-1.68	1077.6005[M-H-Ac-Xyl]$^-$, 945.5560[M-H-Ac-Xyl-ara(p)]$^-$, 783.4991[M-H-Ac-Xyl- ara(p)-Glc]$^-$, 621.4448[M-H-Ac-Xyl- ara(p)-2Glc]$^-$, 459.3903[M-H-Ac-Xyl- ara(p)-3Glc]$^-$	Ginsenoside Ra_5 isomer
R279	18.25	$C_{57}H_{94}O_{26}$	1193.5947	-0.67	1107.5950[M-H-Malonyl]$^-$, 945.5453[M-H-Malonyl-Glc]$^-$, 783.4878[M-H-Malonyl-2Glc]$^-$, 621.4369[M-H-Malonyl-3Glc]$^-$, 459.3831[M-H-Malonyl-4Glc]$^-$	Malonyl-ginsenosid e Rb_1
R280	18.25	$C_{58}H_{96}O_{26}$	1207.6130	1.49	1075.5701[M-H-Xyl]$^-$, 943.5261[M-H-Xyl-Ara]$^-$, 781.4768[M-H-Xyl-Ara-Glc]$^-$, 619.4244[M-H-Xyl-Ara-2Glc]$^-$, 457.3690[M-H-Xyl-Ara-3Glc]$^-$	Dehydrated-protop anaxatriol+Xyl+Ara +3Glc
R281	18.29	$C_{42}H_{72}O_{13}$	783.4897	0.26	637.4318[M-H-Rha]$^-$, 475.3785[M-H-Rha-Glc]$^-$	Ginsenoside 20(*R*)-Rg_2 [a]
R282	18.29	$C_{50}H_{78}O_{20}$	1043.5052 [b]	-1.05	793.4557[M-H-Ac-Glc]$^-$, 731.4312[M-H-Ac-Glc-CO_2-H_2O]$^-$, 613.3776[M-H-Ac-2Glc-H_2O]$^-$, 569.3872[M-H-Ac-2Glc-H_2O-CO_2]$^-$, 455.3525[M-H-Ac-2Glc-Glu A]$^-$	Acetyl-ginsenoside Ro
R283	18.29	$C_{61}H_{102}O_{28}$	1281.6483	0.31	1239.6411[M-H-Ac]$^-$, 1107.5958[M-H-Ac-Xyl/Ara]$^-$, 945.5457[M-H-Ac-Xyl/Ara-Glc]$^-$, 783.4905[M-H-Ac-Xyl/Ara-2Glc]$^-$,	Acetyl-ginsenoside Ra_3/Acetyl-notogins enoside R_4

					621.4393[M-H-Ac-Xyl/Ara-3Glc]$^-$, 459.3840[M-H-Ac-Xyl/Ara-4Glc]$^-$	
R284	18.39	$C_{62}H_{102}O_{30}$	1325.6387	0.68	1239.6389[M-H-Malonyl]$^-$, 1107.59573[M-H-Malonyl-Xyl]$^-$, 945.5438[M-H-Malonyl-Xyl-Glc]$^-$, 783.4891[M-H-Malonyl-Xyl-2Glc]$^-$, 621.4385[M-H-Malonyl-Xyl-3Glc]$^-$, 459.3851[M-H-Malonyl-Xyl-4Glc]$^-$	Malonyl-notoginsenoside R_4 isomer/Malonyl-ginsenoside Ra_3 isomer
R285	18.46	$C_{53}H_{90}O_{22}$	1077.5856	1.02	945.5432[M-H-Ara(f)]$^-$, 783.4899[M-H-Ara(f)-Glc]$^-$, 621.4377[M-H-Ara(f)-2Glc]$^-$, 459.3862[M-H-Ara(f)-3Glc]$^-$	Ginsenoside Rc [a]
R286	18.48	$C_{36}H_{62}O_9$	637.4317	0.16	475.3794[M-H-Glc]$^-$	Ginsenoside 20(*R*)-Rh_1 [a]
R287	18.49	$C_{60}H_{100}O_{27}$	1251.6374	0.00	1077.5972[M-H-Ac-Xyl]$^-$, 945.5435[M-H-Ac-Xyl-ara(p)]$^-$, 783.4909[M-H-Ac-Xyl- ara(p)-Glc]$^-$, 621.4369[M-H-Ac-Xyl- ara(p)-2Glc]$^-$, 459.3839[M-H-Ac-Xyl- ara(p)-3Glc]$^-$	Ginsenoside Ra_5 isomer
R288	18.50	$C_{63}H_{104}O_{31}$	1355.6470	-0.96	1269.5906[M-H-Malonyl]$^-$, 1107.6016[M-H-Malonyl-Glc]$^-$, 945.5383[M-H-Malonyl-2Glc]$^-$, 783.4890[M-H-Malonyl-3Glc]$^-$, 621.4332 [M-H-Malonyl-4Glc]$^-$, 459.3844[M-H-Malonyl-5Glc]$^-$	Protopanaxadiol+4 Glc+Malonyl Glc
R289	18.54	$C_{58}H_{98}O_{26}$	1209.6272	0.33	1077.5841[M-H-Xyl]$^-$, 945.5413[M-H-Xyl-Ara(p)]$^-$, 783.4902[M-H-Xyl-Ara(p)-Glc]$^-$, 621.4371[M-H-Xyl-Ara(p)-2Glc]$^-$,	Ginsenoside Ra_1 [a]

					459.3835[M-H-Xyl-Ara(p)-3Glc]$^-$	
R290	18.56	$C_{60}H_{100}O_{27}$	1251.6355	-1.52	1077.5945[M-H-Ac-Xyl]$^-$, 945.5418[M-H-Ac-Xyl-ara(p)]$^-$, 783.4897[M-H-Ac-Xyl- ara(p)-Glc]$^-$, 621.4377[M-H-Ac-Xyl- ara(p)-2Glc]$^-$, 459.3839[M-H-Ac-Xyl- ara(p)-3Glc]$^-$	Ginsenoside Ra_5 isomer
R291	18.57	$C_{62}H_{102}O_{29}$	1309.6445	1.22	1223.6338[M-H-Malonyl]$^-$, 1077.5852[M-H-Malonyl-Rha]$^-$, 945.5426[M-H-Malonyl-Rha-Xyl/Ara]$^-$, 783.4899[M-H-Malonyl-Rha-Xyl/Ara-Glc]$^-$, 621.4382[M-H-Malonyl-Rha-Xyl/Ara-2 Glc]$^-$, 459.3824[M-H-Malonyl-Rha-Xyl/Ara-3 Glc]$^-$	Protopanaxadiol+ Rha+Xyl/Ara+2Glc+ Malonyl Glc
R292	18.58	$C_{57}H_{94}O_{26}$	1193.5937	-1.51	1107.5844[M-H-Malonyl]$^-$, 945.5449[M-H-Malonyl-Glc]$^-$, 783.4917[M-H-Malonyl-2Glc]$^-$, 621.4375[M-H-Malonyl-3Glc]$^-$, 459.3895 [M-H-Malonyl-4Glc]$^-$	Malonyl-ginsenoside Rb_1 isomer
R293	18.59	$C_{59}H_{100}O_{27}$	1239.6377	0.24	1107.5945[M-H-Glc]$^-$, 945.5399[M-H-Glc-Xyl]$^-$, 783.4880[M-H-2Glc-Xyl]$^-$, 621.4394[M-H-3Glc-Xyl]$^-$, 459.3839[M-H-4Glc-Xyl]$^-$	Notoginsenoside R_4 isomer/Ginsenoside Ra_3 isomer
R294	18.60	$C_{62}H_{102}O_{30}$	1325.6361	-1.28	1239.6375[M-H-Malonyl]$^-$, 1107.5938[M-H-Malonyl-Xyl]$^-$, 945.5428[M-H-Malonyl-Xyl-Glc]$^-$, 783.4893[M-H-Malonyl-Xyl-2Glc]$^-$, 621.4374[M-H-Malonyl-Xyl-3Glc]$^-$, 459.3838[M-H-Malonyl-Xyl-4Glc]$^-$	Malonyl-notoginsenoside R_4 isomer/Malonyl-ginsenoside Ra_3 isomer

R295	18.60	$C_{54}H_{90}O_{24}$	1121.5722	-1.96	1077.5829[M-H-CO_2]$^-$, 945.5391[M-H-Glu A]$^-$, 783.4904[M-H-Glu A-Glc]$^-$, 621.4357[M-H-Glu A-2Glc]$^-$, 459.3828[M-H-Glu A-3Glc]$^-$	Protopanaxadiol+Glu A+3Glc
R296	18.61	$C_{54}H_{92}O_{23}$	1107.5950	-0.09	945.5437[M-H-Glc]$^-$, 783.4900[M-H-2Glc]$^-$, 621.4373[M-H-3Glc]$^-$, 459.3863[M-H-4Glc]	Ginsenoside Rb_1 isomer
R297	18.61	$C_{60}H_{96}O_{29}$	1279.5959	0.00	1107.5977[M-H-2Malonyl]$^-$, 945.5449[M-H-2Malonyl-Glc]$^-$, 783.4931[M-H-2Malonyl-2Glc]$^-$, 621.4354[M-H-2Malonyl-3Glc]$^-$, 459.3859[M-H-2Malonyl-4Glc]$^-$	Di-malonyl-ginsenoside Rb_1 isomer
R298	18.63	$C_{53}H_{88}O_{22}$	1075.5700	1.02	943.5248[M-H-Xyl/Ara]$^-$, 781.4762[M-H-Xyl/Ara-Glc]$^-$, 619.4203[M-H-Xyl/Ara-2Glc]$^-$, 457.3685[M-H-Xyl/Ara-3Glc]$^-$	Dehydrated-protopanaxatriol+3Glc+Xyl/Ara
R299	18.66	$C_{44}H_{74}O_{14}$	825.4988	-1.45	783.4869[M-H-Ac]$^-$, 637.4297[M-H-Ac-Rha]$^-$, 475.3792[M-H-Ac-Rha-Glc]$^-$	Acetyl-ginsenoside Rg_2
R300	18.69	$C_{54}H_{90}O_{24}$	1121.5761	1.52	1077.5846[M-H-CO_2]$^-$, 945.5435[M-H-Glu A]$^-$, 783.4907[M-H-Glu A-Glc]$^-$, 621.4352[M-H-Glu A-2Glc]$^-$, 459.3841[M-H-Glu A-3Glc]$^-$	Protopanaxadiol+Glu A+3Glc
R301	18.70	$C_{48}H_{76}O_{19}$	955.4899	-0.42	793.4385[M-H-Glc]$^-$, 731.4386[M-H-Glc-CO_2-H_2O]$^-$,	Ginsenoside Ro [a]

					613.3731[M-H-2Glc-H_2O]$^-$, 569.3847[M-H-2Glc-H_2O-CO_2]$^-$, 455.3528[M-H-2Glc-Glu A]$^-$	
R302	18.71	$C_{60}H_{100}O_{27}$	1251.6389	1.20	1209.6281[M-H-Ac]$^-$, 1077.5845[M-H-Ac-Xyl]$^-$, 945.5412[M-H-Ac-Xyl-ara(p)]$^-$, 783.4886[M-H-Ac-Xyl- ara(p)-Glc]$^-$, 621.4381[M-H-Ac-Xyl- ara(p)-2Glc]$^-$, 459.3839[M-H-Ac-Xyl- ara(p)-3Glc]$^-$	Ginsenoside Ra_5 isomer
R303	18.71	$C_{55}H_{92}O_{23}$	1119.5968	1.25	1077.5806[M-H-Ac]$^-$, 945.5352[M-H-Ac-Ara(p)]$^-$, 783.4962[M-H-Ac-Ara(p)-Glc]$^-$, 621.4344[M-H-Ac-Ara(p)-2Glc]$^-$, 459.3892[M-H-Ac-Ara(p)-3Glc]$^-$	Ginsenoside Rs_2 isomer
R304	18.73	$C_{42}H_{66}O_{14}$	793.4357	-2.14	631.3856[M-H-Glc]$^-$, 455.3538[M-H-Glc-Glu A]$^-$	Chikusetsusaponin Iva isomer
R305	18.75	$C_{56}H_{94}O_{24}$	1149.6046	-0.96	1107.5938[M-H-Ac]$^-$, 945.5453[M-H-Ac-Glc]$^-$, 783.4894[M-H-Ac-2Glc]$^-$, 621.4366[M-H-Ac-3Glc]$^-$, 459.3822[M-H-Ac-4Glc]$^-$	Quinquenoside R_1 isomer
R306	18.77	$C_{50}H_{78}O_{20}$	1043.5060 [b]	-0.29	955.4778[M-H-Ac]$^-$, 793.4501[M-H-Ac-Glc]$^-$, 731.4313[M-H-Ac-Glc-CO_2-H_2O]$^-$, 613.3796[M-H-Ac-2Glc-H_2O]$^-$, 569.3850[M-H-Ac-2Glc-H_2O-CO_2]$^-$, 455.3524[M-H-Ac-2Glc-Glu A]$^-$	Acetyl-ginsenoside Ro
R307	18.78	$C_{56}H_{92}O_{25}$	1163.5837	-1.03	1077.5847[M-H-Malonyl]$^-$, 945.5422[M-H-Malonyl-Ara(f)]$^-$, 783.4894[M-H-Malonyl-Ara(f)-Glc]$^-$, 621.4321[M-H-Malonyl-Ara(f)-2Glc]$^-$,	Malonyl-ginsenoside Rc

					459.3841[M-H-Malonyl-Ara(f)-3Glc]-	
R308	18.78	$C_{57}H_{94}O_{25}$	1177.6007	0.08	945.5461[M-H-Malonyl-Rha]-, 783.4894[M-H-Malonyl-Rha-Glc]-, 621.4360[M-H-Malonyl-Rha-2Glc]-, 459.3818[M-H-Malonyl-Rha-3Glc]-	Protopanaxadiol+ Rha+2Glc+ Malonyl Glc
R309	18.80	$C_{51}H_{84}O_{21}$	1031.5433	0.58	945.5455[M-H-Malonyl]-, 783.4893[M-H-Malonyl-Glc]-, 621.4326[M-H-Malonyl-2Glc]-, 459.3844[M-H-Malonyl-3Glc]-	Malonyl-ginsenosid e Rd isomer
R310	18.80	$C_{61}H_{100}O_{29}$	1295.6268	-0.31	1209.6307[M-H-Malonyl]-, 1077.5858[M-H-Malonyl-Xyl] -, 945.5420[M-H-Malonyl-Xyl-Ara] -, 783.4886[M-H-Malonyl-Xyl-Ara-Glc] -, 621.4398[M-H-Malonyl-Xyl-Ara-2Glc] -, 459.3825[M-H-Malonyl-Xyl-Ara-3Glc] -	Malonyl-ginsenosid e Ra_1/ Malonyl-ginsenosid e Ra_2
R311	18.81	$C_{42}H_{72}O_{14}$	799.4856	1.50	637.4308[M-H-Glc]-, 475.3791[M-H-2Glc]-	Ginsenoside Rf isomer
R312	18.86	$C_{58}H_{98}O_{26}$	1209.6247	-1.74	1077.5752[M-H-Xyl]-, 945.5474[M-H-Xyl-Ara]-, 783.4880[M-H-Xyl-Ara-Glc]-, 621.4359[M-H-Xyl-Ara-2Glc]-, 459.3845[M-H-Xyl-Ara-3Glc]-	Ginsenoside Ra_1 isomer/Ginsenoside Ra_2 isomer
R313	18.87	$C_{60}H_{96}O_{29}$	1279.5957	-0.16	1107.5918[M-H-2Malonyl]-, 945.5438[M-H-2Malonyl-Glc]-, 783.4860[M-H-2Malonyl-2Glc]-, 621.4385[M-H-2Malonyl-3Glc]-, 459.3857[M-H-2Malonyl-4Glc]-	Di-malonyl-ginseno side Rb_1
R314	18.91	$C_{55}H_{92}O_{23}$	1119.5965	0.98	1077.5841[M-H-Ac]-, 945.5428[M-H-Ac-Ara(p)]-,	Ginsenoside Rs_2 isomer

					783.4896[M-H-Ac-Ara(p)-Glc]$^-$, 621.4369[M-H-Ac-Ara(p)-2Glc]$^-$, 459.3840[M-H-Ac-Ara(p)-3Glc]$^-$	
R315	18.91	$C_{48}H_{76}O_{19}$	955.4904	0.10	793.4389[M-H-Glc]$^-$, 731.4376[M-H-Glc-CO_2-H_2O]$^-$, 613.3753[M-H-2Glc-H_2O]$^-$, 569.3854[M-H-2Glc-H_2O-CO_2]$^-$, 455.3521[M-H-2Glc-Glu A]$^-$	Ginsenoside Ro isomer
R316	18.96	$C_{62}H_{102}O_{30}$	1325.6381	0.23	1239.6345[M-H-Malonyl]$^-$, 1107.5945[M-H-Malonyl-Xyl]$^-$, 945.5399[M-H-Malonyl-Xyl-Glc]$^-$, 783.4880[M-H-Malonyl-Xyl-2Glc]$^-$, 621.4394[M-H-Malonyl-Xyl-3Glc]$^-$, 459.3839[M-H-Malonyl-Xyl-4Glc]$^-$	Malonyl-notoginsenoside R_4 isomer/Malonyl-ginsenoside Ra_3 isomer
R317	18.96	$C_{44}H_{74}O_{14}$	825.4994	-0.73	783.4867[M-H-Ac]$^-$, 637.4291[M-H-Ac-Rha]$^-$, 475.3800[M-H-Ac-Rha-Glc]$^-$	Acetyl-ginsenoside Rg_2
R318	18.96	$C_{54}H_{90}O_{24}$	1121.5755	0.98	1077.5817[M-H-CO_2]$^-$, 945.5435[M-H-Glu A]$^-$, 783.4898[M-H-Glu A-Glc]$^-$, 621.4363[M-H-Glu A-2Glc]$^-$, 459.3836[M-H-Glu A-3Glc]$^-$	Protopanaxadiol+Glu A+3Glc
R319	19.03	$C_{58}H_{98}O_{26}$	1209.6290	1.82	1077.5848[M-H-Xyl]$^-$, 945.5434[M-H-Xyl-Ara]$^-$, 783.4905[M-H-Xyl-Ara-Glc]$^-$, 621.4381[M-H-Xyl-Ara-2Glc]$^-$, 459.3849[M-H-Xyl-Ara-3Glc]$^-$	Ginsenoside Ra_1 isomer/Ginsenoside Ra_2 isomer
R320	19.03	$C_{57}H_{94}O_{26}$	1193.5947	-0.67	1107.5983[M-H-Malonyl]$^-$, 945.5463[M-H-Malonyl-Glc]$^-$, 783.4893[M-H-Malonyl-2Glc]$^-$, 621.4371[M-H-Malonyl-3Glc]$^-$,	Malonyl-ginsenoside Rb_1 isomer

					459.3842[M-H-Malonyl-4Glc]-	
R321	19.07	$C_{43}H_{72}O_{14}$	857.4911 [b]	1.40	637.4381[M-H-Ac-Xyl/Ara]-, 475.3815[M-H-Ac-Xyl/Ara-Glc]-	Protopanaxatriol+ Xyl/Ara+Acetyl Glc
R322	19.08	$C_{60}H_{102}O_{28}$	1269.6456	-1.81	1107.5911[M-H-Glc]-, 945.5439[M-H-2Glc]-, 783.4919[M-H-3Glc]-, 621.4399[M-H-4Glc]-, 459.3881[M-H-5Glc]-	Protopanaxadiol+5 Glc
R323	19.08	$C_{47}H_{74}O_{18}$	925.4802	0.54	793.4316[M-H-Xyl/Ara]-, 763.4323[M-H-Glc]-, 613.3762[M-H-Xyl/Ara-Glc-H_2O]-, 455.3538[M-H-Xyl/Ara-Glc-Glu A]-	Pseudo-ginsenoside -RT_1 isomer/Chikusetsus aponin IV isomer
R324	19.10	$C_{53}H_{90}O_{22}$	1077.5839	-0.56	945.5392[M-H-Ara(p)]-, 783.4884[M-H-Ara(p)-Glc]-, 621.4386[M-H-Ara(p)-2Glc]-, 459.3867[M-H-Ara(p)-3Glc]-	Ginsenoside Rb_2 [a]
R325	19.12	$C_{61}H_{102}O_{28}$	1281.6491	0.94	1239.6388[M-H-Ac]-, 1107.5963[M-H-Ac-Xyl/Ara]-, 945.5442[M-H-Ac-Xyl/Ara-Glc]-, 783.4909[M-H-Ac-Xyl/Ara-2Glc]-, 621.4378[M-H-Ac-Xyl/Ara-3Glc]-, 459.3850[M-H-Ac-Xyl/Ara-4Glc]-	Acetyl-ginsenoside Ra_3/Acetyl-notogins enoside R_4
R326	19.13	$C_{60}H_{100}O_{27}$	1251.6381	0.56	1209.6289[M-H-Ac]-, 1077.5865[M-H-Ac-Xyl]-, 945.5471[M-H-Ac-Xyl-ara(p)]-, 783.4902[M-H-Ac-Xyl- ara(p)-Glc]-, 621.4373[M-H-Ac-Xyl- ara(p)-2Glc]-, 459.3838[M-H-Ac-Xyl- ara(p)-3Glc]-	Ginsenoside Ra_5 isomer
R327	19.14	$C_{53}H_{82}O_{20}$	1083.5381	0.46	955.4847[M-H-Malonyl-Ac]-,	Malonyl+Acetyl ginsenoside Ro

					793.4380[M-H-Malonyl-Ac-Glc]$^-$, 731.4424[M-H-Malonyl-Ac-Glc-CO_2-H_2O]$^-$, 613.3773[M-H-Malonyl-Ac-2Glc-H_2O]$^-$, 569.3868[M-H-Malonyl-Ac-2Glc-H_2O-CO_2]$^-$, 455.3549[M-H-Malonyl-Ac-2Glc-Glu A]$^-$	
R328	19.17	$C_{61}H_{100}O_{29}$	1295.6271	-0.08	1209.6307[M-H-Malonyl]$^-$, 1077.5858[M-H-Malonyl-Xyl]$^-$, 945.5420[M-H-Malonyl-Xyl-Ara]$^-$, 783.4886[M-H-Malonyl-Xyl-Ara-Glc]$^-$, 621.4398[M-H-Malonyl-Xyl-Ara-2Glc]$^-$, 459.3825[M-H-Malonyl-Xyl-Ara-3Glc]$^-$	Malonyl-ginsenoside Ra_1/ Malonyl-ginsenoside Ra_2
R329	19.21	$C_{42}H_{72}O_{14}$	799.4840	-0.50	637.4321[M-H-Glc]$^-$, 475.3770[M-H-2Glc]$^-$	Ginsenoside Rf isomer
R330	19.22	$C_{42}H_{66}O_{14}$	793.4386	1.51	631.3849[M-H-Glc]$^-$, 613.3763[M-H-Glc-H_2O]$^-$, 569.3846[M-H-Glc-H_2O-CO_2]$^-$, 455.3526[M-H-Glc-Glu A]$^-$	Chikusetsusaponin Iva isomer
R331	19.23	$C_{60}H_{100}O_{27}$	1251.6051	-1.84	1209.6217[M-H-Ac]$^-$, 1077.5846[M-H-Ac-Xyl]$^-$, 945.5435[M-H-Ac-Xyl-ara(p)]$^-$, 783.4898[M-H-Ac-Xyl- ara(p)-Glc]$^-$, 621.4362[M-H-Ac-Xyl- ara(p)-2Glc]$^-$, 459.3823[M-H-Ac-Xyl- ara(p)-3Glc]$^-$	Ginsenoside Ra_5 isomer
R332	19.23	$C_{62}H_{102}O_{30}$	1325.6381	0.23	1239.6373[M-H-Malonyl]$^-$, 1107.5939[M-H-Malonyl-Xyl]$^-$,	Malonyl-notoginsenoside R_4 isomer/Malonyl-gin

					945.5414[M-H-Malonyl-Xyl-Glc]-, 783.4899[M-H-Malonyl-Xyl-2Glc]-, 621.4370[M-H-Malonyl-Xyl-3Glc]-, 459.3854[M-H-Malonyl-Xyl-4Glc]-	senoside Ra_3 isomer
R333	19.23	$C_{36}H_{60}O_9$	681.4225 [b]	1.61	473.3669[M-H-Glc]-	Dehydrogenated-protopanaxatriol+Glc
R334	19.24	$C_{60}H_{96}O_{29}$	1279.5957	-0.16	1107.5880[M-H-2Malonyl]-, 945.5450[M-H-2Malonyl-Glc]-, 783.4890[M-H-2Malonyl-2Glc]-, 621.4353[M-H-2Malonyl-3Glc]-, 459.3817[M-H-2Malonyl-4Glc]-	Di-malonyl-ginsenoside Rb_1 isomer
R335	19.28	$C_{53}H_{90}O_{22}$	1077.5830	-1.39	945.5438[M-H-Xyl]-, 783.4917[M-H-Xyl-Glc]-, 621.4368[M-H-Xyl-2Glc]-, 459.3821[M-H-Xyl-3Glc]-	Ginsenoside Rb_3 [a]
R336	19.31	$C_{54}H_{92}O_{23}$	1107.5949	-0.18	945.5427[M-H-Glc]-, 783.4897[M-H-2Glc]-, 621.4379[M-H-3Glc]-, 459.3847[M-H-4Glc]	Ginsenoside Rb_1 isomer
R337	19.37	$C_{51}H_{78}O_{22}$	1041.4895	-1.06	997.5104[M-H-CO_2]-, 955.4934[M-H-Malonyl]-, 937.4802[M-H-Malonyl-H_2O]-, 835.4501[M-H-CO_2-Glc]-, 793.4376[M-H-Malonyl-Glc]-, 731.4386[M-H-Malonyl-Glc-CO_2-H_2O]-, 613.3726[M-H-Malonyl-2Glc-H_2O]-, 569.3844[M-H-Malonyl-2Glc-H_2O-CO_2	Malonyl-ginsenoside Ro

					]$^-$, 455.3516[M-H-Malonyl-2Glc-Glu A]$^-$	
R338	19.37	$C_{62}H_{102}O_{29}$	1309.6443	1.07	1223.6461[M-H-Malonyl]$^-$, 1077.5978[M-H-Malonyl-Rha]$^-$, 945.5418[M-H-Malonyl-Rha-Xyl/Ara]$^-$, 783.4874[M-H-Malonyl-Rha-Xyl/Ara-Glc]$^-$, 621.4355[M-H-Malonyl-Rha-Xyl/Ara-2 Glc]$^-$, 459.3845[M-H-Malonyl-Rha-Xyl/Ara-3 Glc]$^-$	Protopanaxadiol+ Rha+Xyl/Ara+2Glc+ Malonyl Glc
R339	19.39	$C_{56}H_{92}O_{25}$	1163.5847	-0.17	1077.5862[M-H-Malonyl]$^-$, 945.5449[M-H-Malonyl-Ara(p)]$^-$, 783.4901[M-H-Malonyl-Ara(p)-Glc]$^-$, 621.4371[M-H-Malonyl-Ara(p)-2Glc]$^-$, 459.3861[M-H-Malonyl-Ara(p)-3Glc]$^-$	Malonyl-ginsenoside Rb_2
R340	19.41	$C_{53}H_{88}O_{22}$	1075.5670	-1.77	943.5227[M-H-Xyl/Ara]$^-$, 781.4702[M-H-Xyl/Ara-Glc]$^-$, 619.4219[M-H-Xyl/Ara-2Glc]$^-$, 457.3678[M-H-Xyl/Ara-3Glc]$^-$	Dehydrated-protopanaxatriol+3Glc+Xyl /Ara
R341	19.42	$C_{36}H_{64}O_{10}$	701.4478 [b]	0.29	493.3874[M-H-Glc]$^-$	Double bond hydrated-protopanaxatriol+Glc
R342	19.42	$C_{40}H_{68}O_{12}$	785.4696 [b]	1.15	607.4248[M-H-Xyl]$^-$, 475.3793[M-H-Xyl-Ara]$^-$	Protopanaxatriol+ Xyl+Ara
R343	19.43	$C_{44}H_{68}O_{15}$	835.4500	2.39	793.4376[M-H-Ac]$^-$, 775.4375[M-H-Ac-H_2O]$^-$, 631.3806[M-H-Ac-Glc]$^-$, 613.3728[M-H-Ac-Glc-H_2O]$^-$, 455.3527[M-H-Ac-Glc-Glu A]$^-$	Acetyl-zingibroside R_1

R344	19.45	$C_{47}H_{74}O_{18}$	925.4798	0.11	793.4359[M-H-Xyl]-, 763.4279[M-H-Glc]-, 613.3743[M-H-Xyl-Glc-H_2O]-, 455.3523[M-H-Xyl-Glc-Glu A]-	Pseudoginsenoside-RT_1
R345	19.49	$C_{58}H_{98}O_{26}$	1209.6259	-0.74	1077.5853[M-H-Xyl]-, 945.5446[M-H-Xyl-Ara]-, 783.4912[M-H-Xyl-Ara-Glc]-, 621.4352[M-H-Xyl-Ara-2Glc]-, 459.3871[M-H-Xyl-Ara-3Glc]-	Ginsenoside Ra_1 isomer/Ginsenoside Ra_2 isomer
R346	19.49	$C_{48}H_{76}O_{18}$	939.4953	0.00	777.4439[M-H-Glc]-, 733.4515[M-H-Glc-CO_2]-, 715.4413[M-H-Glc-CO_2-H_2O]-, 613.3743[M-H-Glc-Rha-H_2O]-, 569.3852[M-H-Glc-Rha-H_2O-CO_2]-, 455.3532[M-H-Glc-Rha-Glu A]-	Oleanolic aglycone+ Glc+Rha+Glu A
R347	19.52	$C_{60}H_{96}O_{29}$	1279.5951	-0.63	1107.5962[M-H-2Malonyl]-, 945.5401[M-H-2Malonyl-Glc]-, 783.4888[M-H-2Malonyl-2Glc]-, 621.4383[M-H-2Malonyl-3Glc]-, 459.3836[M-H-2Malonyl-4Glc]-	Di-malonyl-ginsenoside Rb_1 isomer
R348	19.54	$C_{47}H_{74}O_{18}$	925.4796	-0.11	793.4389[M-H-Ara]-, 613.3744[M-H-Ara-Glc-H_2O]-, 455.3550[M-H-Ara-Glc-Glu A]-	Chikusetsusaponin IV
R349	19.55	$C_{56}H_{92}O_{25}$	1163.5842	-0.60	1077.5854[M-H-Malonyl]-, 945.5452[M-H-Malonyl-Xyl]-, 783.4902[M-H-Malonyl-Xyl-Glc]-, 621.4372[M-H-Malonyl-Xyl-2Glc]-, 459.3836[M-H-Malonyl-Xyl-3Glc]-	Malonyl-ginsenoside Rb_3
R350	19.55	$C_{50}H_{78}O_{20}$	997.5024	1.60	955.4882[M-H-Ac]-, 793.4370[M-H-Ac-Glc]-, 731.4380[M-H-Ac-Glc-CO_2-H_2O]-,	Acetyl ginsenoside Ro

					613.3746[M-H-Ac-2Glc-H_2O]$^-$, 569.3854[M-H-Ac-2Glc-H_2O-CO_2]$^-$, 455.3538[M-H-Ac-2Glc-Glu A]$^-$	
R351	19.59	$C_{53}H_{90}O_{22}$	1077.5831	-1.30	945.5436[M-H-Xyl/Ara]$^-$, 783.4892[M-H-Xyl/Ara-Glc]$^-$, 621.4373[M-H-Xyl/Ara-2Glc]$^-$, 459.3839[M-H-Xyl/Ara-3Glc]$^-$	Ginsenoside Rc isomer/Ginsenoside Rb_2 isomer/Ginsenoside Rb_3 isomer
R352	19.62	$C_{60}H_{96}O_{29}$	1279.5959	0.00	1107.5955[M-H-2Malonyl]$^-$, 945.5436[M-H-2Malonyl-Glc]$^-$, 783.4926[M-H-2Malonyl-2Glc]$^-$, 621.4370[M-H-2Malonyl-3Glc]$^-$, 459.3842[M-H-2Malonyl-4Glc]$^-$	Di-malonyl-ginsenoside Rb_1 isomer
R353	19.69	$C_{52}H_{80}O_{20}$	1023.5167	0.20	793.4422[M-H-(*E*)-but-2-enoyl-Glc]$^-$, 613.3751[M-H-(*E*)-but-2-enoyl-2Glc-H_2O]$^-$, 569.3848[M-H-(*E*)-but-2-enoyl-2Glc-H_2O-CO_2]$^-$, 455.3507[M-H-(*E*)-but-2-enoyl-2Glc-Glu A]$^-$	(*E*)-But-2-enoyl ginsenoside Ro
R354	19.70	$C_{54}H_{90}O_{24}$	1121.5757	1.16	1077.5817[M-H-CO_2]$^-$, 945.5435[M-H-Glu A]$^-$, 783.4898[M-H-Glu A-Glc]$^-$, 621.4363[M-H-Glu A-2Glc]$^-$, 459.3836[M-H-Glu A-3Glc]$^-$	Protopanaxadiol+Glu A+3Glc
R355	19.72	$C_{57}H_{94}O_{26}$	1193.5941	-1.17	1107.5844[M-H-Malonyl]$^-$, 945.5494[M-H-Malonyl-Glc]$^-$, 783.4945[M-H-Malonyl-2Glc]$^-$, 621.4383[M-H-Malonyl-3Glc]$^-$, 459.3871[M-H-Malonyl-4Glc]$^-$	Malonyl-ginsenoside Rb_1 isomer
R356	19.75	$C_{53}H_{88}O_{23}$	1137.5699 [b]	0.53	1091.6007[M-H]$^-$,	Protopanaxadiol+Rha+3Glc

					945.5386[M-H-Rha]-, 783.4919[M-H-Rha-Glc]-, 621.4356[M-H-Rha-2Glc]-, 459.3863[M-H-Rha-3Glc]-	
R357	19.76	$C_{60}H_{100}O_{27}$	1251.6399	2.00	1209.6260[M-H-Ac]-, 1077.5836[M-H-Ac-Xyl]-, 945.5417[M-H-Ac-Xyl-ara(p)]-, 783.4888[M-H-Ac-Xyl- ara(p)-Glc]-, 621.4368[M-H-Ac-Xyl- ara(p)-2Glc]-, 459.3839[M-H-Ac-Xyl- ara(p)-3Glc]-	Ginsenoside Ra_5 isomer
R358	19.76	$C_{56}H_{94}O_{24}$	1149.6056	-0.09	1107.5945[M-H-Ac]-, 945.5436[M-H-Ac-Glc]-, 783.4880[M-H-Ac-2Glc]-, 621.4371[M-H-Ac-3Glc]-, 459.3827 [M-H-Ac-4Glc]-	Quinquenoside R_1 [a]
R359	19.81	$C_{54}H_{92}O_{23}$	1107.5951	0.00	945.5396[M-H-Glc]-, 783.4886[M-H-2Glc]-, 621.4380[M-H-3Glc]-, 459.3831[M-H-4Glc]-	Ginsenoside Rb_1 isomer
R360	19.81	$C_{48}H_{80}O_{18}$	943.5251	-1.59	781.4766[M-H-Glc]-, 619.4234[M-H-2Glc]-, 457.3694[M-H-3Glc]-	Dehydrated-protop anaxatriol+3Glc
R361	19.84	$C_{42}H_{72}O_{14}$	799.4847	0.38	637.4288[M-H-Glc]-, 475.3797[M-H-2Glc]-	Ginsenoside Rf isomer
R362	19.84	$C_{42}H_{66}O_{14}$	793.4374	0.00	631.3944[M-H-Glc]-, 455.3521[M-H-Glc-Glu A]-	Chikusetsusaponin Iva isomer
R363	19.85	$C_{51}H_{84}O_{21}$	1031.5433 [b]	0.58	943.5408[M-H-Ac]-, 781.4747[M-H-Ac-Glc]-, 763.4615[M-H-Ac-Glc-H_2O]-,	Dehydrated-protop anaxatriol+Acetyl Glc+2Glc

					619.4192[M-H-Ac-2Glc]$^{-}$, 457.3654[M-H-Ac-3Glc]$^{-}$	
R364	19.86	$C_{50}H_{84}O_{19}$	987.5538	0.91	945.5446[M-H-Ac]$^{-}$, 783.4880[M-H-Ac-Glc]$^{-}$, 621.4377[M-H-Ac-2Glc]$^{-}$, 459.3817[M-H-Ac-3Glc]$^{-}$	Acetyl-ginsenoside Rd
R365	19.87	$C_{57}H_{94}O_{25}$	1177.6007	0.08	945.5461[M-H-Malonyl-Rha]$^{-}$, 783.4894[M-H-Malonyl-Rha-Glc]$^{-}$, 621.4360[M-H-Malonyl-Rha-2Glc]$^{-}$, 459.3818[M-H-Malonyl-Rha-3Glc]$^{-}$	Protopanaxadiol+ Rha+2Glc+Malonyl Glc
R366	19.89	$C_{50}H_{82}O_{20}$	1047.5387 [b]	1.05	915.5361[M-H-Malonyl]$^{-}$, 783.4871[M-H-Malonyl-Xyl/Ara]$^{-}$, 621.4372[M-H-Malonyl-Xyl/Ara-Glc]$^{-}$, 459.3854[M-H-Malonyl-Xyl/Ara-2Glc]$^{-}$	Protopanaxadio+Xyl/Ara+Glc+malonyl Glc
R367	19.91	$C_{61}H_{102}O_{28}$	1281.6464	-1.17	1239.6306[M-H-Ac]$^{-}$, 1107.5948[M-H-Ac-Xyl/Ara]$^{-}$, 945.5497[M-H-Ac-Xyl/Ara-Glc]$^{-}$, 783.4878[M-H-Ac-Xyl/Ara-2Glc]$^{-}$, 621.4354[M-H-Ac-Xyl/Ara-3Glc]$^{-}$, 459.3835[M-H-Ac-Xyl/Ara-4Glc]$^{-}$	Acetyl-ginsenoside Ra_3/Acetyl-notoginsenoside R_4
R368	19.92	$C_{47}H_{74}O_{18}$	925.4786	-1.19	793.4379[M-H-Xyl/Ara]$^{-}$, 763.4317[M-H-Glc]$^{-}$, 613.3748[M-H-Xyl/Ara-Glc-H_2O]$^{-}$, 455.3550[M-H-Xyl/Ara-Glc-Glu A]$^{-}$	Pseudo-ginsenoside -RT_1 isomer/Chikusetsusaponin IV isomer
R369	19.92	$C_{61}H_{100}O_{29}$	1295.6278	0.46	1209.6318[M-H-Malonyl]$^{-}$, 1077.5848[M-H-Malonyl-Xyl]$^{-}$, 945.5450[M-H-Malonyl-Xyl-Ara]$^{-}$, 783.4901[M-H-Malonyl-Xyl-Ara-Glc]$^{-}$,	Malonyl-ginsenoside Ra_1/Malonyl-ginsenoside Ra_2

					621.4370[M-H-Malonyl-Xyl-Ara-2Glc]$^-$, 459.3867[M-H-Malonyl-Xyl-Ara-3Glc]$^-$	
R370	19.94	$C_{60}H_{100}O_{27}$	1251.6377	0.24	1209.6288[M-H-Ac]$^-$, 1077.5878[M-H-Ac-Xyl]$^-$, 945.5459[M-H-Ac-Xyl-ara(p)]$^-$, 783.4923[M-H-Ac-Xyl- ara(p)-Glc]$^-$, 621.4391[M-H-Ac-Xyl- ara(p)-2Glc]$^-$, 459.3857[M-H-Ac-Xyl- ara(p)-3Glc]$^-$	Ginsenoside Ra$_5$ isomer
R371	19.94	$C_{42}H_{72}O_{14}$	799.4850	0.75	637.4313[M-H-Glc]$^-$, 475.3790[M-H-2Glc]$^-$	Ginsenoside Rf isomer
R372	19.95	$C_{57}H_{94}O_{23}$	1145.6084	-2.09	945.5468[M-H-(*E*)-but-2-enoyl-Ara]$^-$, 783.4857[M-H-(*E*)-but-2-enoyl-Ara-Glc]$^-$, 621.4389[M-H-(*E*)-but-2-enoyl-Ara-2Glc]$^-$, 459.3842[M-H-(*E*)-but-2-enoyl-Ara-3Glc]$^-$	Ginsenoside Ra$_7$ ismoer/Ginsenoside Ra$_8$ isomer/Ginsenoside Ra$_9$ isomer
R373	19.97	$C_{57}H_{94}O_{26}$	1193.5945	-0.84	1107.5961[M-H-Malonyl]$^-$, 945.5444[M-H-Malonyl-Glc]$^-$, 783.4915[M-H-Malonyl-2Glc]$^-$, 621.4359[M-H-Malonyl-3Glc]$^-$, 459.3847[M-H-Malonyl-4Glc]$^-$	Malonyl-ginsenoside Rb$_1$ isomer
R374	19.98	$C_{51}H_{78}O_{22}$	1041.4905	-0.10	997.5105[M-H-CO_2]$^-$, 955.4903[M-H-Malonyl]$^-$, 937.4792[M-H-Malonyl-H_2O]$^-$, 835.4482[M-H-CO_2-Glc]$^-$, 793.4376[M-H-Malonyl-Glc]$^-$, 731.4372[M-H-Malonyl-Glc-CO_2-H_2O]$^-$, 613.3749[M-H-Malonyl-2Glc-H_2O]$^-$, 569.3847[M-H-Malonyl-2Glc-H_2O-CO_2	Malonyl-ginsenoside Ro

					]$^{-}$, 455.3528[M-H-Malonyl-2Glc-Glu A]$^{-}$	
R375	19.98	$C_{53}H_{88}O_{23}$	1137.5675 [b]	-1.58	1091.5061[M-H]$^{-}$, 945.5502[M-H-Rha]$^{-}$, 783.4934[M-H-Rha-Glc]$^{-}$, 621.4410[M-H-Rha-2Glc]$^{-}$, 459.3882[M-H-Rha-3Glc]$^{-}$	Protopanaxadiol+Rha+3Glc
R376	19.99	$C_{56}H_{94}O_{24}$	1149.6063	0.52	1107.5995[M-H-Ac]$^{-}$, 945.5463[M-H-Ac-Glc]$^{-}$, 783.4916[M-H-Ac-2Glc]$^{-}$, 621.4371[M-H-Ac-3Glc]$^{-}$, 459.3832[M-H-Ac-4Glc]$^{-}$	Quinquenoside R_1 isomer
R377	20.00	$C_{50}H_{82}O_{20}$	1047.5400 [b]	2.29	915.5372[M-H-Malonyl]$^{-}$, 753.4806[M-H-Malonyl-Glc]$^{-}$, 621.4372[M-H-Malonyl-Glc-Xyl/Ara]$^{-}$, 459.3854[M-H-Malonyl-2Glc-Xyl/Ara]$^{-}$	Protopanaxadio+Xyl/Ara+Glc+ malonyl Glc
R378	20.01	$C_{50}H_{78}O_{20}$	997.5012	0.40	955.4804[M-H-Ac]$^{-}$, 793.4379[M-H-Ac-Glc]$^{-}$, 731.4380[M-H-Ac-Glc-CO_2-H_2O]$^{-}$, 613.3776[M-H-Ac-2Glc-H_2O]$^{-}$, 569.3849[M-H-Ac-2Glc-H_2O-CO_2]$^{-}$, 455.3527[M-H-Ac-2Glc-Glu A]$^{-}$	Acetyl ginsenoside Ro
R379	20.06	$C_{54}H_{90}O_{24}$	1121.5760	1.43	1077.5817[M-H-CO_2]$^{-}$, 945.5435[M-H-Glu A]$^{-}$, 783.4898[M-H-Glu A-Glc]$^{-}$, 621.4363[M-H-Glu A-2Glc]$^{-}$, 459.3836[M-H-Glu A-3Glc]$^{-}$	Protopanaxadiol+Glu A+3Glc
R380	20.07	$C_{53}H_{88}O_{22}$	1075.5671	-1.67	943.5288[M-H-Xyl/Ara]$^{-}$, 781.4751[M-H-Xyl/Ara-Glc]$^{-}$,	Dehydrated-protopanaxatriol+3Glc+Xyl/Ara

					619.4372[M-H-Xyl/Ara-2Glc]$^-$, 457.3701[M-H-Xyl/Ara-3Glc]$^-$	
R381	20.10	$C_{48}H_{82}O_{17}$	929.5488	1.51	767.4883[M-H-Glc]$^-$, 605.4429[M-H-2Glc]$^-$, 443.3901[M-H-3Glc]$^-$	Vina-ginsenosideR3
R382	20.11	$C_{50}H_{84}O_{19}$	987.5524	-0.51	621.4370[M-H-Ac-2Glc]$^-$, 459.3854[M-H-Ac-3Glc]$^-$	Acetyl-ginsenoside Rd
R383	20.13	$C_{53}H_{90}O_{22}$	1077.5834	-1.02	945.5458[M-H-Xyl/Ara]$^-$, 783.4869[M-H-Xyl/Ara-Glc]$^-$, 621.4376[M-H-Xyl/Ara-2Glc]$^-$, 459.3828[M-H-Xyl/Ara-3Glc]$^-$	Ginsenoside Rc isomer/Ginsenoside Rb_2 isomer/Ginsenoside Rb_3 isomer
R384	20.13	$C_{54}H_{90}O_{24}$	1121.5736	-0.71	1077.5930[M-H-CO_2]$^-$, 945.5456[M-H-Glu A]$^-$, 783.4904[M-H-Glu A-Glc]$^-$, 621.4372[M-H-Glu A-2Glc]$^-$, 459.3824[M-H-Glu A-3Glc]$^-$	Protopanaxadiol+Glu A+3Glc
R385	20.16	$C_{48}H_{76}O_{19}$	955.4908	0.52	793.4373[M-H-Glc]$^-$, 731.4371[M-H-Glc-CO_2-H_2O]$^-$, 613.3723[M-H-2Glc-H_2O]$^-$, 569.3849[M-H-2Glc-H_2O-CO_2]$^-$, 455.3546[M-H-2Glc-Glu A]$^-$	Ginsenoside Ro isomer
R386	20.16	$C_{42}H_{66}O_{14}$	793.4380	0.76	631.3845[M-H-Glc]$^-$, 613.3694[M-H-Glc-H_2O]$^-$, 569.3824[M-H-Glc-H_2O-CO_2]$^-$, 455.3539[M-H-Glc-Glu A]$^-$	Chikusetsusaponin Iva isomer
R387	20.20	$C_{56}H_{92}O_{25}$	1163.5840	-0.77	1077.5858[M-H-Malonyl]$^-$, 945.5435[M-H-Malonyl-Xyl]$^-$, 783.4874[M-H-Malonyl-Xyl-Glc]$^-$, 621.4385[M-H-Malonyl-Xyl-2Glc]$^-$,	Malonyl-ginsenoside Rc/Malonyl-ginsenoside Rb_2/Malonyl-ginsen

					459.3840[M-H-Malonyl-Xyl-3Glc]$^-$	oside Rb_3
R388	20.21	$C_{50}H_{82}O_{19}$	985.5356	-1.62	943.5279[M-H-Ac]$^-$, 781.4781[M-H-Ac-Glc]$^-$, 619.4225[M-H-Ac-2Glc]$^-$, 457.3687[M-H-Ac-3Glc]$^-$	Dehydrated-protopanaxatriol+2Glc+Acetyl Glc
R389	20.22	$C_{50}H_{84}O_{19}$	987.5529	0.00	945.5433[M-H-Ac]$^-$, 783.4898[M-H-Ac-Glc]$^-$, 621.4373[M-H-Ac-2Glc]$^-$, 459.3843[M-H-Ac-3Glc]$^-$	Acetyl-ginsenoside Rd
R390	20.22	$C_{42}H_{72}O_{14}$	799.4855	1.38	637.4311[M-H-Glc]$^-$, 475.3783[M-H-2Glc]$^-$	Ginsenoside Rf isomer
R391	20.22	$C_{53}H_{86}O_{22}$	1073.5514	-1.68	945.5403[M-H-Malonyl-Ac]$^-$, 783.4893[M-H-Malonyl-Ac-Glc]$^-$, 621.4393[M-H-Malonyl-Ac-2Glc]$^-$, 459.3838[M-H-Malonyl-Ac-3Glc]$^-$	Acetyl malonyl-ginsenoside Rd
R392	20.22	$C_{55}H_{86}O_{22}$	1097.5515	-1.55	1011.4634[M-H-Malonyl]$^-$, 943.5273[M-H-Malonyl-(*E*)-but-2-enoyl]$^-$, 781.4724[M-H-Malonyl-(*E*)-but-2-enoyl-Glc]$^-$, 619.4225[M-H-Malonyl-(*E*)-but-2-enoyl-2Glc]$^-$, 457.3666[M-H-Malonyl-(*E*)-but-2-enoyl-3Glc]$^-$	Dehydrated-protopanaxatriol+3Glc+Malonyl+(*E*)-but-2-enoyl
R393	20.24	$C_{53}H_{90}O_{22}$	1077.5870	2.32	945.5370[M-H-Xyl/Ara]$^-$, 783.4865[M-H-Xyl/Ara-Glc]$^-$, 621.4413[M-H-Xyl/Ara-2Glc]$^-$, 459.3839[M-H-Xyl/Ara-3Glc]$^-$	Ginsenoside Rc isomer/Ginsenoside Rb_2 isomer/Ginsenoside Rb_3 isomer
R394	20.25	$C_{56}H_{94}O_{24}$	1149.6073	1.39	1107.5934[M-H-Ac]$^-$, 945.5349[M-H-Ac-Glc]$^-$, 783.4432[M-H-Ac-2Glc]$^-$,	Quinquenoside R_1 isomer

					621.4408[M-H-Ac-3Glc]$^-$, 459.3830[M-H-Ac-4Glc]$^-$	
R395	20.25	$C_{42}H_{66}O_{14}$	793.4375	0.13	631.3785[M-H-Glc]$^-$, 455.3568[M-H-Glc-Glu A]$^-$	Chikusetsusaponin Iva
R396	20.28	$C_{60}H_{100}O_{27}$	1251.6399	2.00	1209.6232[M-H-Ac]$^-$, 1077.5782[M-H-Ac-Xyl]$^-$, 945.5428[M-H-Ac-Xyl-ara(p)]$^-$, 783.4841[M-H-Ac-Xyl- ara(p)-Glc]$^-$, 621.4386[M-H-Ac-Xyl- ara(p)-2Glc]$^-$, 459.3860[M-H-Ac-Xyl- ara(p)-3Glc]$^-$	Ginsenoside Ra_5
R397	20.29	$C_{52}H_{80}O_{20}$	1023.5171	0.59	955.4908[M-H-(*E*)-but-2-enoyl]$^-$, 793.4405[M-H-(*E*)-but-2-enoyl-Glc]$^-$, 455.3537[M-H-(*E*)-but-2-enoyl-2Glc-Glu A]$^-$	(*E*)-But-2-enoyl ginsenoside Ro
R398	20.31	$C_{61}H_{102}O_{28}$	1281.6474	-0.39	1239.6392[M-H-Ac]$^-$, 1107.5917[M-H-Ac-Xyl/Ara]$^-$, 945.5443[M-H-Ac-Xyl/Ara-Glc]$^-$, 783.4888[M-H-Ac-Xyl/Ara-2Glc]$^-$, 621.4402[M-H-Ac-Xyl/Ara-3Glc]$^-$, 459.3836[M-H-Ac-Xyl/Ara-4Glc]$^-$	Acetyl-ginsenoside Ra_3/Acetyl-notoginsenoside R_4
R399	20.34	$C_{56}H_{92}O_{25}$	1163.5846	-0.26	1077.5853[M-H-Malonyl]$^-$, 945.5468[M-H-Malonyl-Xyl]$^-$, 783.4906[M-H-Malonyl-Xyl-Glc]$^-$, 621.4352[M-H-Malonyl-Xyl-2Glc]$^-$, 459.3855[M-H-Malonyl-Xyl-3Glc]$^-$	Malonyl-ginsenoside Rc/Malonyl-ginsenoside Rb_2/Malonyl-ginsenoside Rb_3
R400	20.36	$C_{54}H_{85}O_{24}$	1117.5428	-0.27	945.5454[M-H-2Malonyl]$^-$, 783.4838[M-H-2Malonyl-Glc]$^-$, 621.4314[M-H-2Malonyl-2Glc]$^-$, 459.3779[M-H-2Malonyl-3Glc]$^-$	Di-malonyl-ginsenoside Rd isomer

R401	20.38	$C_{55}H_{92}O_{23}$	1119.5958	0.36	1077.5853[M-H-Ac]-, 945.5424[M-H-Ac-Ara(f)]-, 783.4914[M-H-Ac-Ara(f)-Glc]-, 621.4385[M-H-Ac-Ara(f)-2Glc]-, 459.3856[M-H-Ac-Ara(f)-3Glc]-	Ginsenoside Rs_2 [a]
R402	20.40	$C_{54}H_{92}O_{23}$	1107.5929	-1.99	945.5450[M-H-Glc]-, 783.4948[M-H-2Glc]-, 621.4366[M-H-3Glc]-, 459.3872[M-H-4Glc]-	Ginsenoside Rb_1 isomer
R403	20.43	$C_{53}H_{86}O_{22}$	1073.5518	-1.30	945.5427[M-H-Malonyl-Ac]-, 783.4915[M-H-Malonyl-Ac-Glc]-, 621.4370[M-H-Malonyl-Ac-2Glc]-, 459.3838[M-H-Malonyl-Ac-3Glc]-	Acetyl malonyl-ginsenoside Rd
R404	20.44	$C_{51}H_{78}O_{22}$	1041.4911	0.48	997.4968[M-H-CO_2]-, 835.4471[M-H-CO_2-Glc]-, 793.4371[M-H-Malonyl-Glc]-, 731.4387[M-H-Malonyl-Glc-CO_2-H_2O]-, 613.3772[M-H-Malonyl-2Glc-H_2O]-, 569.3847[M-H-Malonyl-2Glc-H_2O-CO_2]-, 455.3516[M-H-Malonyl-2Glc-Glu A]-	Malonyl-ginsenoside Ro
R405	20.46	$C_{50}H_{78}O_{20}$	1041.5047 [b]	-1.53	793.4395[M-H-Ac-Glc]-, 731.4311[M-H-Ac-Glc-CO_2-H_2O]-, 613.3771[M-H-Ac-2Glc-H_2O]-, 569.3856[M-H-Ac-2Glc-H_2O-CO_2]-, 455.3533[M-H-Ac-2Glc-Glu A]-	Acetyl-ginsenoside Ro
R406	20.47	$C_{48}H_{82}O_{18}$	945.5446	2.33	783.4901[M-H-Glc]-, 621.4373[M-H-2Glc]-, 459.3842[M-H-3Glc]-	Ginsenoside Rd [a]

R407	20.49	$C_{51}H_{78}O_{22}$	1041.4917 [b]	1.06	997.5104[M-H-CO2]-, 835.4457[M-H-CO2-Glc]-, 793.4380[M-H-Malonyl-Glc]-, 731.4377[M-H-Malonyl-Glc-CO2-H2O]-, 613.3754 [M-H-Malonyl-2Glc-H2O]-, 569.3849[M-H-Malonyl-2Glc-H2O-CO2]-, 455.3526[M-H-Malonyl-2Glc-Glu A]-	Malonyl-ginsenoside Ro
R408	20.50	$C_{57}H_{94}O_{25}$	1177.6014	0.68	1091.5942[M-H-Malonyl]-, 945.5306[M-H-Malonyl-Rha]-, 783.5013[M-H-Malonyl-Rha-Glc]-, 621.4434[M-H-Malonyl-Rha-2Glc]-, 459.3832[M-H-Malonyl-Rha-3Glc]-	Protopanaxadiol+ Rha+2Glc+ Malonyl Glc
R409	20.51	$C_{56}H_{92}O_{25}$	1163.5844	-0.43	1077.5852[M-H-Malonyl]-, 945.5432[M-H-Malonyl-Xyl]-, 783.4932[M-H-Malonyl-Xyl-Glc]-, 621.4381[M-H-Malonyl-Xyl-2Glc]-, 459.3842[M-H-Malonyl-Xyl-3Glc]-	Malonyl-ginsenoside Rc/Malonyl-ginsenoside Rb_2/Malonyl-ginsenoside Rb_3
R410	20.51	$C_{42}H_{66}O_{14}$	793.4380	0.76	631.3845[M-H-Glc]-, 455.3537[M-H-Glc-Glu A]-	Chikusetsusaponin Iva isomer
R411	20.56	$C_{54}H_{92}O_{23}$	1107.5964	1.17	945.5460[M-H-Glc]-, 783.4965[M-H-2Glc]-, 621.4393[M-H-3Glc]-, 459.3831[M-H-4Glc]-	Ginsenoside Rb_1 isomer
R412	20.57	$C_{57}H_{94}O_{26}$	1193.5935	-1.68	1107.5872[M-H-Malonyl]-, 945.5467[M-H-Malonyl-Glc]-, 783.4908[M-H-Malonyl-2Glc]-, 621.4413[M-H-Malonyl-3Glc]-, 459.3840[M-H-Malonyl-4Glc]-	Malonyl-ginsenoside Rb_1 isomer

R413	20.58	$C_{62}H_{102}O_{29}$	1309.6423	-0.46	1223.6443[M-H-Malonyl]-, 1077.5836[M-H-Malonyl-Rha]-, 945.5417[M-H-Malonyl-Rha-Xyl/Ara]-, 783.4929[M-H-Malonyl-Rha-Xyl/Ara-Glc]-, 621.4376[M-H-Malonyl-Rha-Xyl/Ara-2 Glc]-, 459.3863[M-H-Malonyl-Rha-Xyl/Ara-3 Glc]-	Protopanaxadiol+ Rha+Xyl/Ara+2Glc+ Malonyl Glc
R414	20.60	$C_{51}H_{84}O_{21}$	1031.5428	0.10	945.5450[M-H-Malonyl]-, 783.4902[M-H-Malonyl-Glc]-, 621.4332[M-H-Malonyl-2Glc]-, 459.3843[M-H-Malonyl-3Glc]-	Malonyl-ginsenoside Rd isomer
R415	20.63	$C_{52}H_{86}O_{20}$	1029.5641	0.68	945.5451[M-H-2Ac]-, 783.4882[M-H-2Ac-Glc]-, 621.4320[M-H-2Ac-2Glc]-, 459.3834[M-H-2Ac-3Glc]-	Acetyl-pseudoginsenoside RC_1
R416	20.69	$C_{56}H_{92}O_{25}$	1163.5850	0.09	1077.5864[M-H-Malonyl]-, 945.5447[M-H-Malonyl-Xyl]-, 783.4913[M-H-Malonyl-Xyl-Glc]-, 621.4382[M-H-Malonyl-Xyl-2Glc]-, 459.3841[M-H-Malonyl-Xyl-3Glc]-	Malonyl-ginsenoside Rc/Malonyl-ginsenoside Rb_2/Malonyl-ginsenoside Rb_3
R417	20.69	$C_{57}H_{94}O_{25}$	1177.6024	1.53	1091.5985[M-H-Malonyl]-, 945.5323[M-H-Malonyl-Rha]-, 783.4916[M-H-Malonyl-Rha-Glc]-, 621.4364[M-H-Malonyl-Rha-2Glc]-, 459.3833[M-H-Malonyl-Rha-3Glc]-	Protopanaxadiol+ Rha+2Glc+ Malonyl Glc
R418	20.69	$C_{52}H_{84}O_{20}$	1027.5476	-0.19	943.5278[M-H-2Ac]-, 925.5139[M-H-2Ac-H_2O]-, 781.4760[M-H-2Ac-Glc]-, 763.4612[M-H-2Ac-Glc-H_2O]-,	Dehydrated-protopanaxatriol+2Acetyl+ 3Glc

					619.4198[M-H-2Ac-2Glc]$^-$, 457.3698[M-H-2Ac-3Glc]$^-$	
R419	20.70	$C_{60}H_{100}O_{27}$	1251.6403	2.32	1209.6299[M-H-Ac]$^-$, 1077.5831[M-H-Ac-Xyl]$^-$, 945.5436[M-H-Ac-Xyl- ara(p)]$^-$, 783.4929[M-H-Ac-Xyl- ara(p)-Glc]$^-$, 621.4393[M-H-Ac-Xyl- ara(p)-2Glc]$^-$, 459.3872[M-H-Ac-Xyl- ara(p)-3Glc]$^-$	Ginsenoside Ra_5 isomer
R420	20.70	$C_{56}H_{94}O_{24}$	1149.6053	-0.35	1107.5966[M-H-Ac]$^-$, 945.5464[M-H-Ac-Glc]$^-$, 783.4889 [M-H-Ac-2Glc]$^-$, 621.4332[M-H-Ac-3Glc]$^-$, 459.3873[M-H-Ac-4Glc]$^-$	Quinquenoside R_1 isomer
R421	20.70	$C_{53}H_{88}O_{23}$	1091.5658	1.83	945.5308[M-H-Rha]$^-$, 783.4868[M-H-Rha-Glc]$^-$, 621.4352[M-H-Rha-2Glc]$^-$, 459.3866[M-H-Rha-3Glc]$^-$	Protopanaxadiol+R ha+3Glc
R422	20.75	$C_{53}H_{90}O_{22}$	1077.5868	2.13	945.5454[M-H-Xyl/Ara]$^-$, 783.4930[M-H-Xyl/Ara-Glc]$^-$, 621.4349[M-H-Xyl/Ara-2Glc]$^-$, 459.3842[M-H-Xyl/Ara-3Glc]$^-$	Ginsenoside Rc isomer/Ginsenoside Rb_2 isomer/Ginsenoside Rb_3 isomer
R423	20.75	$C_{51}H_{84}O_{21}$	1031.5424	-0.29	945.5437[M-H-Malonyl]$^-$, 783.4904[M-H-Malonyl-Glc]$^-$, 621.4370[M-H-Malonyl-2Glc]$^-$, 459.3864[M-H-Malonyl-3Glc]$^-$	Malonyl-ginsenosid e Rd
R424	20.76	$C_{48}H_{76}O_{18}$	939.4968	1.60	777.4389[M-H-Glc]$^-$, 715.4407[M-H-Glc-CO_2-H_2O]$^-$, 631.3880[M-H-Glc-Rha]$^-$, 613.3713[M-H-Glc-Rha-H_2O]$^-$, 569.3848[M-H-Glc-Rha-H_2O-CO_2]$^-$,	Oleanolic aglycone+ Glc+Rha+Glu A

					455.3532[M-H-Glc-Rha-Glu A]-	
R425	20.85	$C_{54}H_{85}O_{24}$	1117.5428	-0.27	945.5441[M-H-2Malonyl]-, 783.4751[M-H-2Malonyl-Glc]-, 621.4387[M-H-2Malonyl-2Glc]-, 459.3778[M-H-2Malonyl-3Glc]-	Di-malonyl-ginsenoside Rd isomer
R426	20.86	$C_{53}H_{88}O_{23}$	1091.5620	-1.65	945.5422[M-H-Rha]-, 783.4988[M-H-Rha-Glc]-, 621.4345[M-H-Rha-2Glc]-, 459.3867[M-H-Rha-3Glc]-	Protopanaxadiol+Rha+3Glc
R427	20.87	$C_{38}H_{64}O_{10}$	679.4432	1.62	475.3795[M-H-Ac-Glc]-	Protopanaxatriol+Acetyl Glc
R428	20.91	$C_{51}H_{84}O_{21}$	1031.5427	0.00	945.5433[M-H-Malonyl]-, 783.4901[M-H-Malonyl-Glc]-, 621.4374[M-H-Malonyl-2Glc]-, 459.3849[M-H-Malonyl-3Glc]-	Malonyl-ginsenoside Rd isomer
R429	20.91	$C_{50}H_{84}O_{19}$	987.5532	0.30	945.5436[M-H-Ac]-, 783.4965[M-H-Ac-Glc]-, 621.4378[M-H-Ac-2Glc]-, 459.3877[M-H-Ac-3Glc]-	Acetyl-ginsenoside Rd
R430	20.95	$C_{55}H_{92}O_{23}$	1119.5951	-0.27	1077.5840[M-H-Ac]-, 945.5427[M-H-Ac-Ara(p)]-, 783.4883[M-H-Ac-Ara(p)-Glc]-, 621.4360[M-H-Ac-Ara(p)-2Glc]-, 459.3837[M-H-Ac-Ara(p)-3Glc]-	Ginsenoside Rs_2 isomer
R431	20.95	$C_{56}H_{94}O_{24}$	1149.6058	0.09	1107.5840[M-H-Ac]-, 945.5446[M-H-Ac-Glc]-, 783.4883[M-H-Ac-2Glc]-, 621.4358[M-H-Ac-3Glc]-, 459.3831[M-H-Ac-4Glc]-	Quinquenoside R_1 isomer
R432	20.98	$C_{57}H_{94}O_{26}$	1193.5936	-1.59	1107.6016[M-H-Malonyl]-, 945.5438[M-H-Malonyl-Glc]-, 783.4922[M-H-Malonyl-2Glc]-, 621.4422[M-H-Malonyl-3Glc]-,	Malonyl-ginsenoside Rb_1 isomer

					459.3853[M-H-Malonyl-4Glc]$^-$	
R433	20.98	$C_{47}H_{74}O_{18}$	925.4797	0.00	793.4382[M-H-Xyl/Ara]$^-$, 763.4349[M-H-Glc]$^-$, 613.3756[M-H-Xyl/Ara-Glc-H_2O]$^-$, 455.3536[M-H-Xyl/Ara-Glc-Glu A]$^-$	Pseudo-ginsenoside -RT_1 isomer/Chikusetsus aponin IV isomer
R434	20.99	$C_{51}H_{82}O_{21}$	1029.5279	0.00	943.5275[M-H-Malonyl]$^-$, 781.4807[M-H-Malonyl-Glc]$^-$, 763.4688[M-H-2Ac-Glc-H_2O]$^-$, 619.4215[M-H-Malonyl-2Glc]$^-$, 457.3689[M-H-Malonyl-3Glc]$^-$	Dehydrated-protop anaxatriol+Malonyl Glc+2Glc
R435	21.03	$C_{54}H_{92}O_{23}$	1107.5936	-1.35	945.5419[M-H-Glc]$^-$, 783.4886[M-H-2Glc]$^-$, 621.4385[M-H-3Glc]$^-$, 459.3854[M-H-4Glc]$^-$	Ginsenoside Rb_1 isomer
R436	21.05	$C_{53}H_{88}O_{23}$	1137.5692 [b]	0.09	945.5434[M-H-Rha]$^-$, 783.4969[M-H-Rha-Glc]$^-$, 621.4361[M-H-Rha-2Glc]$^-$, 459.3797[M-H-Rha-3Glc]$^-$	Protopanaxadiol+R ha+3Glc
R437	21.11	$C_{62}H_{102}O_{27}$	1277.6522	-0.63	1209.6308[M-H-(*E*)-but-2-enoyl]$^-$, 1077.5751[M-H-(*E*)-but-2-enoyl-Xyl]$^-$, 945.5579[M-H-(*E*)-but-2-enoyl-Xyl-ara(p)]$^-$, 783.4861[M-H-(*E*)-but-2-enoyl-Xyl-ara(p)-Glc]$^-$, 621.4409[M-H-(*E*)-but-2-enoyl-Xyl-ara(p)-2Glc]$^-$, 459.3841[M-H-(*E*)-but-2-enoyl-Xyl-ara(p)-3Glc]$^-$	Ginsenoside Ra_4 isomer
R438	21.17	$C_{48}H_{76}O_{19}$	955.4912	0.94	793.4358[M-H-Glc]$^-$,	Ginsenoside Ro

					731.4333[M-H-Glc-CO_2-H_2O]$^-$, 613.3773[M-H-2Glc-H_2O]$^-$, 569.3846[M-H-2Glc-H_2O-CO_2]$^-$, 455.3546[M-H-2Glc-Glu A]$^-$	isomer
R439	21.19	$C_{44}H_{74}O_{14}$	825.5000	0.00	783.4904[M-H-Ac]$^-$, 637.4363[M-H-Ac-Rha]$^-$, 619.4239[M-H-Ac-Rha-H_2O]$^-$, 475.3794[M-H-Ac-Rha-Glc]$^-$	Acetyl-ginsenoside Rg_2
R440	21.20	$C_{57}H_{94}O_{26}$	1193.5952	-0.25	1107.5831[M-H-Malonyl]$^-$, 945.5392[M-H-Malonyl-Glc]$^-$, 783.4866[M-H-Malonyl-2Glc]$^-$, 621.4386[M-H-Malonyl-3Glc]$^-$, 459.3846[M-H-Malonyl-4Glc]$^-$	Malonyl-ginsenoside Rb_1 isomer
R441	21.23	$C_{53}H_{86}O_{22}$	1073.5526	-0.56	945.5450[M-H-Malonyl-Ac]$^-$, 783.4891[M-H-Malonyl-Ac-Glc]$^-$, 621.4370[M-H-Malonyl-Ac-2Glc]$^-$, 459.3848[M-H-Malonyl-Ac-3Glc]$^-$	Acetyl malonyl-ginsenoside Rd
R442	21.23	$C_{54}H_{85}O_{24}$	1117.5427	-0.36	945.5416[M-H-2Malonyl]$^-$, 783.4908[M-H-2Malonyl-Glc]$^-$, 621.4375[M-H-2Malonyl-2Glc]$^-$, 459.3872[M-H-2Malonyl-3Glc]$^-$	Di-malonyl-ginsenoside Rd
R443	21.23	$C_{43}H_{68}O_{14}$	807.4536	0.62	765.4451[M-H-Ac]$^-$, 603.3922[M-H-Ac-Glc]$^-$, 441.3367[M-H-Ac-2Glc]$^-$	Dehydrated-protopanaxadiol+Acetyl Glc+Glc
R444	21.25	$C_{52}H_{86}O_{20}$	1029.5630	-0.39	945.5422[M-H-2Ac]$^-$, 783.4902[M-H-2Ac-Glc]$^-$, 621.4365[M-H-2Ac-2Glc]$^-$, 459.3865 [M-H-2Ac-3Glc]$^-$	Acetyl-pseudoginsenoside RC_1
R445	21.25	$C_{47}H_{74}O_{18}$	925.4801	0.43	793.4344[M-H-Xyl/Ara]$^-$, 731.4366[M-H-Xyl/Ara-CO_2-H_2O]$^-$,	Pseudo-ginsenoside-RT_1 isomer/Chikusetsus

					613.3762[M-H-Xyl/Ara-Glc-H_2O]$^-$, 569.3815[M-H-Xyl/Ara-Glc-H_2O-CO_2]$^-$, 455.3551[M-H-Xyl/Ara-Glc-Glu A]$^-$	aponin IV isomer
R446	21.33	$C_{43}H_{68}O_{14}$	807.4531	0.00	765.4421[M-H-Ac]$^-$, 603.3807[M-H-Ac-Glc]$^-$, 441.3345[M-H-Ac-2Glc]$^-$	Dehydrated-protopanaxadiol+Acetyl Glc+Glc
R447	21.35	$C_{53}H_{90}O_{22}$	1077.5837	-0.74	945.5419[M-H-Xyl/Ara]$^-$, 783.4922[M-H-Xyl/Ara-Glc]$^-$, 621.4381[M-H-Xyl/Ara-2Glc]$^-$, 459.3848[M-H-Xyl/Ara-3Glc]$^-$	Ginsenoside Rc isomer/Ginsenoside Rb_2 isomer/Ginsenoside Rb_3 isomer
R448	21.35	$C_{57}H_{94}O_{26}$	1193.5932	-1.93	1107.5895[M-H-Malonyl]$^-$, 945.5457[M-H-Malonyl-Glc]$^-$, 783.4879[M-H-Malonyl-2Glc]$^-$, 621.4395[M-H-Malonyl-3Glc]$^-$, 459.3821[M-H-Malonyl-4Glc]$^-$	Malonyl-ginsenoside Rb_1 isomer
R449	21.37	$C_{57}H_{94}O_{25}$	1177.6016	0.85	1091.5903[M-H-Malonyl]$^-$, 945.5390[M-H-Malonyl-Rha]$^-$, 783.4928[M-H-Malonyl-Rha-Glc]$^-$, 621.4377[M-H-Malonyl-Rha-2Glc]$^-$, 459.3842[M-H-Malonyl-Rha-3Glc]$^-$	Protopanaxadiol+ Rha+2Glc+ Malonyl Glc
R450	21.37	$C_{50}H_{82}O_{19}$	985.5359	-1.32	943.5255[M-H-Ac]$^-$, 781.4734[M-H-Ac-Glc]$^-$, 619.4212[M-H-Ac-2Glc]$^-$, 457.3695[M-H-Ac-3Glc]$^-$	Dehydrated-protopanaxatriol+2Glc+Acetyl Glc
R451	21.37	$C_{52}H_{80}O_{20}$	1023.5179	1.37	731.4371[M-H-(*E*)-but-2-enoyl-Glc-CO_2-H_2O]$^-$, 613.3745[M-H-(*E*)-but-2-enoyl-2Glc-H_2O]$^-$, 569.3858[M-H-(*E*)-but-2-enoyl-2Glc-H_2O-CO_2]$^-$,	(*E*)-But-2-enoyl ginsenoside Ro

					455.3513[M-H-(*E*)-but-2-enoyl-2Glc-Glu A]$^-$	
R452	21.39	$C_{36}H_{60}O_9$	681.4221 ^{b}	1.03	473.3669[M-H-Glc]$^-$	Ginsenoside Rh_7
R453	21.41	$C_{58}H_{96}O_{24}$	1175.6238	2.13	1107.5961[M-H-(*E*)-but-2-enoyl]$^-$,<br>945.5278[M-H-(*E*)-but-2-enoyl-Glc]$^-$,<br>783.4851[M-H-(*E*)-but-2-enoyl-2Glc]$^-$,<br>621.4348[M-H-(*E*)-but-2-enoyl-3Glc]$^-$,<br>459.3815[M-H-(*E*)-but-2-enoyl-4Glc]$^-$	Ginsenoside Ra_6
R454	21.43	$C_{54}H_{85}O_{24}$	1117.5428	-0.27	945.5416[M-H-2Malonyl]$^-$,<br>783.4908[M-H-2Malonyl-Glc]$^-$,<br>621.4375[M-H-2Malonyl-2Glc]$^-$,<br>459.3872[M-H-2Malonyl-3Glc]$^-$	Di-malonyl-ginsenoside Rd isomer
R455	21.44	$C_{60}H_{100}O_{27}$	1251.6401	2.16	1209.6194[M-H-Ac]$^-$,<br>1077.5831[M-H-Ac-Xyl]$^-$,<br>945.5413[M-H-Ac-Xyl- ara(p)]$^-$,<br>783.4884[M-H-Ac-Xyl- ara(p)-Glc]$^-$,<br>621.4351[M-H-Ac-Xyl- ara(p)-2Glc]$^-$,<br>459.3881[M-H-Ac-Xyl- ara(p)-3Glc]$^-$	Ginsenoside Ra_5 isomer
R456	21.49	$C_{63}H_{104}O_{28}$	1307.6643	0.54	1239.6508[M-H-(*E*)-but-2-enoyl]$^-$,<br>1107.5946[M-H-(*E*)-but-2-enoyl-Glc]$^-$,<br>945.5443[M-H-(*E*)-but-2-enoyl-Glc-Xyl]$^-$,<br>783.4894[M-H-(*E*)-but-2-enoyl-2Glc-Xyl]$^-$,<br>621.4363[M-H-(*E*)-but-2-enoyl-3Glc-Xyl]$^-$,<br>459.3818[M-H-(*E*)-but-2-enoyl-4Glc-Xyl]$^-$	(*E*)-But-2-enoyl-ginsenoside Ra_3/(*E*)-But-2-enoyl-notoginsenoside R_4
R457	21.50	$C_{46}H_{76}O_{15}$	867.5114	0.92	799.4860[M-H-(*E*)-but-2-enoyl]$^-$,<br>637.4326[M-H-(*E*)-but-2-enoyl-Glc]$^-$,<br>475.3796[M-H-(*E*)-but-2-enoyl-2Glc]$^-$	Koryoginsenoside R_1/Ginsenoside Re_6
R458	21.54	$C_{48}H_{82}O_{18}$	945.5416	-0.74	783.4871[M-H-Glc]$^-$,	Ginsenoside Rd

					621.4375[M-H-2Glc]$^-$, 459.3847[M-H-3Glc]$^-$	isomer
R459	21.54	$C_{55}H_{92}O_{23}$	1119.5946	-0.71	1077.5846[M-H-Ac]$^-$, 945.5458[M-H-Ac-Ara(p)]$^-$, 783.4878[M-H-Ac-Ara(p)-Glc]$^-$, 621.4374[M-H-Ac-Ara(p)-2Glc]$^-$, 459.3867[M-H-Ac-Ara(p)-3Glc]$^-$	Ginsenoside Rs_2 isomer
R460	21.55	$C_{50}H_{84}O_{19}$	987.5530	0.10	945.5439[M-H-Ac]$^-$, 783.4905[M-H-Ac-Glc]$^-$, 621.4355[M-H-Ac-2Glc]$^-$, 459.3843 [M-H-Ac-3Glc]$^-$	Acetyl-ginsenoside Rd
R461	21.57	$C_{52}H_{86}O_{20}$	1029.5638	0.39	945.5418[M-H-2Ac]$^-$, 783.4908[M-H-2Ac-Glc]$^-$, 621.4369[M-H-2Ac-2Glc]$^-$, 459.3848[M-H-2Ac-3Glc]$^-$	Acetyl-pseudoginsenoside RC_1
R462	21.67	$C_{54}H_{92}O_{23}$	1107.5967	1.44	945.5404[M-H-Glc]$^-$, 783.4837[M-H-2Glc]$^-$, 621.4324[M-H-3Glc]$^-$, 459.3803[M-H-4Glc]$^-$	Ginsenoside Rb_1 isomer
R463	21.70	$C_{42}H_{72}O_{13}$	783.4887	-1.02	475.3744[M-H-Rha-Glc]$^-$	Ginsenoside Rg_2 isomer
R464	21.70	$C_{53}H_{88}O_{23}$	1091.5622	-1.47	945.5419[M-H-Rha]$^-$, 783.4901[M-H-Rha-Glc]$^-$, 621.4414[M-H-Rha-2Glc]$^-$, 459.3853[M-H-Rha-3Glc]$^-$	Protopanaxadiol+Rha+3Glc
R465	21.71	$C_{51}H_{84}O_{21}$	1031.5426	-0.10	945.5422[M-H-Malonyl]$^-$, 783.4875[M-H-Malonyl-Glc]$^-$, 621.4376[M-H-Malonyl-2Glc]$^-$, 459.3843[M-H-Malonyl-3Glc]$^-$	Malonyl-ginsenoside Rd isomer
R466	21.73	$C_{55}H_{92}O_{23}$	1119.5942	-1.07	1077.5846[M-H-Ac]$^-$, 945.5414[M-H-Ac-Ara(p)]$^-$, 783.4888[M-H-Ac-Ara(p)-Glc]$^-$,	Ginsenoside Rs_2 isomer

					621.4365[M-H-Ac-Ara(p)-2Glc]$^-$,<br>459.3842[M-H-Ac-Ara(p)-3Glc]$^-$	
R467	21.74	$C_{58}H_{96}O_{24}$	1175.6234	1.79	1107.5952[M-H-(*E*)-but-2-enoyl]$^-$,<br>945.5443[M-H-(*E*)-but-2-enoyl-Glc]$^-$,<br>783.4917[M-H-(*E*)-but-2-enoyl-2Glc]$^-$,<br>621.4363[M-H-(*E*)-but-2-enoyl-3Glc]$^-$,<br>459.3848[M-H-(*E*)-but-2-enoyl-4Glc]$^-$	Ginsenoside Ra_6 isomer
R468	21.78	$C_{53}H_{88}O_{23}$	1091.5658	1.83	945.5442[M-H-Rha]$^-$,<br>783.4897[M-H-Rha-Glc]$^-$,<br>621.4383[M-H-Rha-2Glc]$^-$,<br>459.3836[M-H-Rha-3Glc]$^-$	Protopanaxadiol+Rha+3Glc
R469	21.82	$C_{47}H_{78}O_{17}$	913.5122	1.84	781.4792[M-H-Xyl/Ara]$^-$,<br>619.4233[M-H-Xyl/Ara-Glc]$^-$,<br>457.3702[M-H-Xyl/Ara-2Glc]$^-$	Dehydrated-protopanaxatriol+Xyl/Ara+2Glc
R470	21.82	$C_{51}H_{82}O_{21}$	1029.5277	0.68	943.5300[M-H-Malonyl]$^-$,<br>781.4759[M-H-Malonyl-Glc]$^-$,<br>763.4671[M-H-2Ac-Glc-H_2O]$^-$,<br>619.4210[M-H-Malonyl-2Glc]$^-$,<br>457.3686[M-H-Malonyl-3Glc]$^-$	Dehydrated-protopanaxatriol+Malonyl Glc+2Glc
R471	21.84	$C_{53}H_{90}O_{22}$	1077.5825	-1.86	945.5468[M-H-Xyl/Ara]$^-$,<br>783.4909[M-H-Xyl/Ara-Glc]$^-$,<br>621.4401[M-H-Xyl/Ara-2Glc]$^-$,<br>459.3853[M-H-Xyl/Ara-3Glc]$^-$	Ginsenoside Rc isomer/Ginsenoside Rb_2 isomer/Ginsenoside Rb_3 isomer
R472	21.84	$C_{53}H_{86}O_{22}$	1073.5526	-0.56	987.5481[M-H-Malonyl]$^-$,<br>945.5444[M-H-Malonyl-Ac]$^-$,<br>783.4901[M-H-Malonyl-Ac-Glc]$^-$,<br>621.4376[M-H-Malonyl-Ac-2Glc]$^-$,<br>459.3846[M-H-Malonyl-Ac-3Glc]$^-$	Acetyl malonyl-ginsenoside Rd
R473	21.85	$C_{52}H_{86}O_{20}$	1029.5643	0.87	945.5439[M-H-2Ac]$^-$,<br>783.4916[M-H-2Ac-Glc]$^-$,	Acetyl-pseudoginsenoside RC_1

					621.4376[M-H-2Ac-2Glc]$^-$, 459.3840[M-H-2Ac-3Glc]$^-$	
R474	21.86	$C_{62}H_{102}O_{27}$	1277.6549	1.49	1209.6573[M-H-(*E*)-but-2-enoyl]$^-$, 1077.5818[M-H-(*E*)-but-2-enoyl-Xyl]$^-$, 945.5376[M-H-(*E*)-but-2-enoyl-Xyl-ara(p)]$^-$, 783.4933[M-H-(*E*)-but-2-enoyl-Xyl-ara(p)-Glc]$^-$, 621.4390[M-H-(*E*)-but-2-enoyl-Xyl-ara(p)-2Glc]$^-$, 459.3856[M-H-(*E*)-but-2-enoyl-Xyl-ara(p)-3Glc]$^-$	Ginsenoside Ra_4
R475	21.86	$C_{42}H_{72}O_{13}$	783.4908	1.66	475.3717[M-H-Rha-Glc]$^-$	Ginsenoside Rg_2 isomer
R476	21.87	$C_{50}H_{84}O_{19}$	987.5538	0.91	945.5443[M-H-Ac]$^-$, 783.4906[M-H-Ac-Glc]$^-$, 621.4380[M-H-Ac-2Glc]$^-$, 459.3850 [M-H-Ac-3Glc]$^-$	Acetyl-ginsenoside Rd
R477	21.89	$C_{51}H_{84}O_{21}$	1031.5430	0.29	945.5432[M-H-Malonyl]$^-$, 783.4888[M-H-Malonyl-Glc]$^-$, 621.4373[M-H-Malonyl-2Glc]$^-$, 459.3849[M-H-Malonyl-3Glc]$^-$	Malonyl-ginsenoside Rd isomer
R478	21.89	$C_{47}H_{80}O_{17}$	915.5320	0.33	621.4365[M-H-Xyl-Glc]$^-$, 459.3826[M-H-Xyl-2Glc]$^-$	Vina-ginsenoside R_{16} isomer
R479	21.92	$C_{57}H_{94}O_{26}$	1193.5929	-2.18	1107.6017[M-H-Malonyl]$^-$, 945.5327[M-H-Malonyl-Glc]$^-$, 783.4842[M-H-Malonyl-2Glc]$^-$, 621.4359[M-H-Malonyl-3Glc]$^-$, 459.3882[M-H-Malonyl-4Glc]$^-$	Malonyl-ginsenoside Rb_1 isomer
R480	21.93	$C_{51}H_{84}O_{19}$	1045.5594 [b]	1.05	1003.4670[M-H-Ac]$^-$, 959.5528[M-H-Malonyl]$^-$, 841.4089[M-H-Ac-Glc]$^-$,	Dehydrogenated-protopanaxatriol+2Glc +Malonyl Glc

					797.5042[M-H-Malonyl-Glc]$^-$, 779.4867[M-H-Malonyl-Glc-H_2O]$^-$, 635.4540[M-H-Malonyl-2Glc]$^-$, 473.4049[M-H-Malonyl-3Glc]$^-$	
R481	21.95	$C_{52}H_{86}O_{20}$	1029.5641	0.68	945.5437[M-H-2Ac]$^-$, 783.4911[M-H-2Ac-Glc]$^-$, 621.4375[M-H-2Ac-2Glc]$^-$, 459.3823[M-H-2Ac-3Glc]$^-$	Acetyl-pseudoginsenoside RC_1
R482	21.96	$C_{58}H_{96}O_{24}$	1175.6223	0.85	1107.5973[M-H-(*E*)-but-2-enoyl]$^-$, 945.5471[M-H-(*E*)-but-2-enoyl-Glc]$^-$, 783.4863[M-H-(*E*)-but-2-enoyl-2Glc]$^-$, 621.4360[M-H-(*E*)-but-2-enoyl-3Glc]$^-$, 459.3829[M-H-(*E*)-but-2-enoyl-4Glc]$^-$	Ginsenoside Ra_6 isomer
R483	21.96	$C_{57}H_{94}O_{23}$	1145.6112	0.35	1077.5985[M-H-(*E*)-but-2-enoyl]$^-$, 945.5368[M-H-(*E*)-but-2-enoyl-Ara]$^-$, 783.4862[M-H-(*E*)-but-2-enoyl-Ara-Glc]$^-$, 621.4388[M-H-(*E*)-but-2-enoyl-Ara-2Glc]$^-$, 459.3850[M-H-(*E*)-but-2-enoyl-Ara-3Glc]$^-$	Ginsenoside Ra_7 ismoer/Ginsenoside Ra_8 isomer/Ginsenoside Ra_9 isomer
R484	21.99	$C_{57}H_{94}O_{25}$	1177.6014	0.68	945.5462[M-H-Malonyl-Rha]$^-$, 783.4944[M-H-Malonyl-Rha-Glc]$^-$, 621.4376[M-H-Malonyl-Rha-2Glc]$^-$, 459.3835[M-H-Malonyl-Rha-3Glc]$^-$	Protopanaxadiol+ Rha+2Glc+ Malonyl Glc
R485	22.03	$C_{57}H_{94}O_{26}$	1193.5934	-1.76	1107.5944[M-H-Malonyl]$^-$, 945.5427[M-H-Malonyl-Glc]$^-$, 783.4875[M-H-Malonyl-2Glc]$^-$, 621.4416[M-H-Malonyl-3Glc]$^-$, 459.3840[M-H-Malonyl-4Glc]$^-$	Malonyl-ginsenoside Rb_1 isomer
R486	22.05	$C_{57}H_{94}O_{23}$	1145.6125	1.48	1077.5834[M-H-(*E*)-but-2-enoyl]$^-$, 945.5418[M-H-(*E*)-but-2-enoyl-Ara]$^-$, 783.4931[M-H-(*E*)-but-2-enoyl-Ara-Glc]$^-$, 621.4370[M-H-(*E*)-but-2-enoyl-Ara-2Gl	Ginsenoside Ra_7/Ginsenoside Ra_8/Ginsenoside Ra_9

					c]-, 459.3860[M-H-(*E*)-but-2-enoyl-Ara-3Glc]-	
R487	22.06	$C_{58}H_{96}O_{25}$	1191.6167 [b]	0.42	945.5418[M-H-(*E*)-but-2-enoyl-ara(f)]-, 783.4931[M-H-(*E*)-but-2-enoyl-ara(f)-Glc]-, 621.4370[M-H-(*E*)-but-2-enoyl-ara(f)-2Glc]-, 459.3807[M-H-(*E*)-but-2-enoyl-ara(f)-3Glc]-	(*E*)-but-2-enoyl-ginsenoside Rc
R488	22.11	$C_{60}H_{100}O_{27}$	1251.6375	0.08	1209.6157[M-H-Ac]-, 1077.5813[M-H-Ac-Xyl]-, 945.5343[M-H-Ac-Xyl- ara(p)]-, 783.4904[M-H-Ac-Xyl- ara(p)-Glc]-, 621.4333[M-H-Ac-Xyl- ara(p)-2Glc]-, 459.3824[M-H-Ac-Xyl- ara(p)-3Glc]-	Ginsenoside Ra_5 isomer
R489	22.11	$C_{52}H_{86}O_{20}$	1029.5642	0.78	945.5402[M-H-2Ac]-, 783.4921[M-H-2Ac-Glc]-, 621.4385[M-H-2Ac-2Glc]-, 459.3853[M-H-2Ac-3Glc]-	Acetyl-pseudoginsenoside RC_1
R490	22.12	$C_{53}H_{86}O_{22}$	1073.5535	0.28	987.5480[M-H-Malonyl]-, 945.5433[M-H-Malonyl-Ac]-, 783.4905[M-H-Malonyl-Ac-Glc]-, 621.4371[M-H-Malonyl-Ac-2Glc]-, 459.3823[M-H-Malonyl-Ac-3Glc]-	Acetyl malonyl-ginsenoside Rd
R491	22.12	$C_{54}H_{85}O_{24}$	1117.5425	-0.54	945.5449[M-H-2Malonyl]-, 783.4940[M-H-2Malonyl-Glc]-, 621.4393[M-H-2Malonyl-2Glc]-, 459.3851[M-H-2Malonyl-3Glc]-	Di-malonyl-ginsenoside Rd isomer
R492	22.13	$C_{48}H_{82}O_{18}$	945.5445	2.33	783.4898[M-H-Glc]-, 621.4384[M-H-2Glc]-,	Ginsenoside Rd isomer

					459.3847[M-H-3Glc]$^{-}$	
R493	22.13	$C_{49}H_{78}O_{19}$	969.5058	-0.10	807.4536[M-H-Glc]$^{-}$, 645.4028[M-H-2Glc]$^{-}$, 455.3528[M-H-2Glc-CH_2-Glu A]$^{-}$	Ginsenoside Ro methyl ester [a]
R494	22.28	$C_{50}H_{82}O_{20}$	1001.5319	-0.20	915.5311[M-H-Malonyl]$^{-}$, 783.4914[M-H-Malonyl-Xyl]$^{-}$, 621.4337[M-H-Malonyl-Xyl-Glc]$^{-}$, 459.3815[M-H-Malonyl-Xyl-2Glc]$^{-}$	Malonyl -vina-ginsenoside R_{16} isomer
R495	22.30	$C_{55}H_{92}O_{23}$	1119.5952	-0.18	1077.5906[M-H-Ac]$^{-}$, 945.5430[M-H-Ac-Ara(p)]$^{-}$, 783.4906[M-H-Ac-Ara(p)-Glc]$^{-}$, 621.4373[M-H-Ac-Ara(p)-2Glc]$^{-}$, 459.3837[M-H-Ac-Ara(p)-3Glc]$^{-}$	Ginsenoside Rs_2 isomer
R496	22.36	$C_{51}H_{84}O_{21}$	1031.5425	-0.19	945.5389[M-H-Malonyl]$^{-}$, 783.4896[M-H-Malonyl-Glc]$^{-}$, 621.4376[M-H-Malonyl-2Glc]$^{-}$, 459.3835[M-H-Malonyl-3Glc]$^{-}$	Malonyl-ginsenosid e Rd isomer
R497	22.37	$C_{57}H_{94}O_{23}$	1145.6097	-0.96	1077.5864[M-H-(*E*)-but-2-enoyl]$^{-}$, 945.5388[M-H-(*E*)-but-2-enoyl-Ara]$^{-}$, 783.4950[M-H-(*E*)-but-2-enoyl-Ara-Glc]$^{-}$, 621.4338[M-H-(*E*)-but-2-enoyl-Ara-2Gl c]$^{-}$, 459.3842[M-H-(*E*)-but-2-enoyl-Ara-3Gl c]$^{-}$	Ginsenoside Ra_7 ismoer/Ginsenoside Ra_8 isomer/Ginsenoside Ra_9 isomer
R498	22.37	$C_{53}H_{90}O_{22}$	1077.5835	-0.93	945.5372[M-H-Xyl/Ara]$^{-}$, 783.4919[M-H-Xyl/Ara-Glc]$^{-}$, 621.4313[M-H-Xyl/Ara-2Glc]$^{-}$, 459.3854[M-H-Xyl/Ara-3Glc]$^{-}$	Ginsenoside Rc isomer/Ginsenoside Rb_2 isomer/Ginsenoside Rb_3 isomer
R499	22.37	$C_{50}H_{84}O_{19}$	987.5544	1.52	945.5461[M-H-Ac]$^{-}$, 783.4906[M-H-Ac-Glc]$^{-}$, 621.4393[M-H-Ac-2Glc]$^{-}$, 459.3840[M-H-Ac-3Glc]$^{-}$	Acetyl-ginsenoside Rd

R500	22.37	$C_{56}H_{94}O_{24}$	1149.6062	0.43	1107.6040[M-H-Ac]$^-$, 945.5499[M-H-Ac-Glc]$^-$, 783.4938 [M-H-Ac-2Glc]$^-$, 621.4362[M-H-Ac-3Glc]$^-$, 459.3951[M-H-Ac-4Glc]$^-$	Quinquenoside R_1 isomer
R501	22.37	$C_{58}H_{96}O_{25}$	1191.6171 [b]	0.76	1077.5836[M-H-(*E*)-but-2-enoyl]$^-$, 945.5418[M-H-(*E*)-but-2-enoyl-ara/xyl]$^-$, 783.4931[M-H-(*E*)-but-2-enoyl-ara/xyl-Glc]$^-$, 621.4370[M-H-(*E*)-but-2-enoyl-ara/xyl-2Glc]$^-$, 459.3838[M-H-(*E*)-but-2-enoyl-ara/xyl-3Glc]$^-$	(*E*)-but-2-enoyl-ginsenoside Rb_2 isomer/(*E*)-but-2-enoyl-ginsenoside Rb_3 isomer/(*E*)-but-2-enoyl-ginsenoside Rc isomer
R502	22.38	$C_{58}H_{96}O_{24}$	1175.6224	0.94	1107.5970[M-H-(*E*)-but-2-enoyl]$^-$, 945.5442[M-H-(*E*)-but-2-enoyl-Glc]$^-$, 783.4881[M-H-(*E*)-but-2-enoyl-2Glc]$^-$, 621.4355[M-H-(*E*)-but-2-enoyl-3Glc]$^-$, 459.3817[M-H-(*E*)-but-2-enoyl-4Glc]$^-$	Ginsenoside Ra_6 isomer
R503	22.42	$C_{42}H_{72}O_{12}$	813.4995 [b]	-0.61	621.4367[M-H-Rha]$^-$, 475.3790[M-H-2Rha]$^-$	Protopanaxatriol+2 Rha
R504	22.43	$C_{48}H_{76}O_{19}$	955.4916	1.36	793.4395[M-H-Glc]$^-$, 731.4410[M-H-Glc-CO_2-H_2O]$^-$, 613.3735[M-H-2Glc-H_2O]$^-$, 569.3845[M-H-2Glc-H_2O-CO_2]$^-$, 455.3530[M-H-2Glc-Glu A]$^-$	Ginsenoside Ro isomer
R505	22.43	$C_{42}H_{66}O_{14}$	793.4387	1.64	631.3829[M-H-Glc]$^-$, 569.3837[M-H-Glc-H_2O-CO_2]$^-$, 455.3611[M-H-Glc-Glu A]$^-$	Zingibroside R_1 isomer
R506	22.45	$C_{50}H_{84}O_{19}$	987.5532	0.30	945.5412[M-H-Ac]$^-$, 783.4760[M-H-Ac-Glc]$^-$, 621.4381[M-H-Ac-2Glc]$^-$, 459.3845[M-H-Ac-3Glc]$^-$	Acetyl-ginsenoside Rd

R507	22.47	$C_{52}H_{86}O_{20}$	1029.5635	0.10	945.5447[M-H-2Ac]$^-$, 783.4893[M-H-2Ac-Glc]$^-$, 621.4305[M-H-2Ac-2Glc]$^-$, 459.3837[M-H-2Ac-3Glc]$^-$	Acetyl-pseudoginsenoside RC_1
R508	22.51	$C_{53}H_{86}O_{22}$	1073.5536	0.37	945.5449[M-H-Malonyl-Ac]$^-$, 783.4931[M-H-Malonyl-Ac-Glc]$^-$, 621.4382[M-H-Malonyl-Ac-2Glc]$^-$, 459.3879[M-H-Malonyl-Ac-3Glc]$^-$	Acetyl malonyl-ginsenoside Rd
R509	22.51	$C_{54}H_{85}O_{24}$	1117.5433	0.18	945.5372[M-H-2Malonyl]$^-$, 783.4890[M-H-2Malonyl-Glc]$^-$, 621.4385[M-H-2Malonyl-2Glc]$^-$, 459.3860[M-H-2Malonyl-3Glc]$^-$	Di-malonyl-ginsenoside Rd isomer
R510	22.53	$C_{47}H_{80}O_{17}$	915.5326	0.98	783.4955[M-H-Xyl]$^-$, 621.4334[M-H-Xyl-Glc]$^-$, 459.3295[M-H-Xyl-2Glc]$^-$	Vina-ginsenoside R_{16}
R511	22.55	$C_{57}H_{94}O_{23}$	1145.6121	1.13	1077.5851[M-H-(*E*)-but-2-enoyl]$^-$, 945.5434[M-H-(*E*)-but-2-enoyl-Ara]$^-$, 783.4898[M-H-(*E*)-but-2-enoyl-Ara-Glc]$^-$, 621.4379[M-H-(*E*)-but-2-enoyl-Ara-2Glc]$^-$, 459.3846[M-H-(*E*)-but-2-enoyl-Ara-3Glc]$^-$	Ginsenoside Ra_7/Ginsenoside Ra_8/Ginsenoside Ra_9
R512	22.55	$C_{58}H_{96}O_{25}$	1191.6162 [b]	0.00	945.5433[M-H-(*E*)-but-2-enoyl-ara(p)]$^-$, 783.4919[M-H-(*E*)-but-2-enoyl-ara(p)-Glc]$^-$, 621.4395[M-H-(*E*)-but-2-enoyl-ara(p)-2Glc]$^-$, 459.3864[M-H-(*E*)-but-2-enoyl-ara(p)-3Glc]$^-$	(*E*)-but-2-enoyl-ginsenoside Rb_2
R513	22.56	$C_{53}H_{90}O_{22}$	1077.5822	-2.13	945.5389[M-H-Xyl/Ara]$^-$, 783.4933[M-H-Xyl/Ara-Glc]$^-$, 621.4375[M-H-Xyl/Ara-2Glc]$^-$, 459.3814[M-H-Xyl/Ara-3Glc]$^-$	Ginsenoside Rc isomer/Ginsenoside Rb_2 isomer/Ginsenoside

						Rb3 isomer
R514	22.56	$C_{51}H_{84}O_{21}$	1031.5422	-0.48	945.5459[M-H-Malonyl]-, 783.4878[M-H-Malonyl-Glc]-, 621.4367[M-H-Malonyl-2Glc]-, 459.3848[M-H-Malonyl-3Glc]-	Malonyl-ginsenoside Rd isomer
R515	22.60	$C_{52}H_{86}O_{20}$	1029.5637	0.29	945.5423[M-H-2Ac]-, 783.4902[M-H-2Ac-Glc]-, 621.4372[M-H-2Ac-2Glc]-, 459.3856[M-H-2Ac-3Glc]-	Acetyl-pseudoginsenoside RC1
R516	22.61	$C_{47}H_{80}O_{17}$	915.5327	1.09	783.4889[M-H-Xyl]-, 621.4374[M-H-Xyl-Glc]-, 459.3845[M-H-Xyl-2Glc]-	Gypenoside IX
R517	22.63	$C_{54}H_{90}O_{24}$	1121.5752	0.71	1077.5817[M-H-CO_2]-, 945.5435[M-H-Glu A]-, 783.4898[M-H-Glu A-Glc]-, 621.4363[M-H-Glu A-2Glc]-, 459.3836[M-H-Glu A-3Glc]-	Protopanaxadiol+Glu A+3Glc
R518	22.65	$C_{62}H_{102}O_{27}$	1277.6528	-0.16	1209.6270[M-H-(*E*)-but-2-enoyl]-, 1077.5861[M-H-(*E*)-but-2-enoyl-Xyl]-, 945.5403[M-H-(*E*)-but-2-enoyl-Xyl-ara(p)]-, 783.4887[M-H-(*E*)-but-2-enoyl-Xyl-ara(p)-Glc]-, 621.4301[M-H-(*E*)-but-2-enoyl-Xyl-ara(p)-2Glc]-, 459.3835[M-H-(*E*)-but-2-enoyl-Xyl-ara(p)-3Glc]-	Ginsenoside Ra4 isomer
R519	22.67	$C_{57}H_{94}O_{23}$	1145.6122	1.22	1077.5808[M-H-(*E*)-but-2-enoyl]-, 945.5503[M-H-(*E*)-but-2-enoyl-Ara]-, 783.4907[M-H-(*E*)-but-2-enoyl-Ara-Glc]-, 621.4350[M-H-(*E*)-but-2-enoyl-Ara-2Glc]-,	Ginsenoside Ra7 ismoer/Ginsenoside Ra8 isomer/Ginsenoside Ra9 isomer

					459.3848[M-H-(*E*)-but-2-enoyl-Ara-3Glc]-	
R520	22.67	$C_{48}H_{82}O_{18}$	945.5441	1.90	783.4864[M-H-Glc]-, 621.4418[M-H-2Glc]-, 475.3783[M-H-2Glc-Rha]-	Protopanaxatriol+2Glc+Rha
R521	22.67	$C_{58}H_{96}O_{25}$	1191.6171 [b]	0.76	945.5412[M-H-(*E*)-but-2-enoyl-ara/xyl]-, 783.4940[M-H-(*E*)-but-2-enoyl-ara/xyl-Glc]-, 621.4380[M-H-(*E*)-but-2-enoyl-ara/xyl-2Glc]-, 459.3842[M-H-(*E*)-but-2-enoyl-ara/xyl-3Glc]-	(*E*)-but-2-enoyl-ginsenoside Rb_2 isomer/(*E*)-but-2-enoyl-ginsenoside Rb_3 isomer/(*E*)-but-2-enoyl-ginsenoside Rc isomer
R522	22.70	$C_{50}H_{82}O_{20}$	1001.5319	-0.20	915.5315[M-H-Malonyl]-, 783.4921[M-H-Malonyl-Xyl]-, 621.4342[M-H-Malonyl-Xyl-Glc]-, 459.3807[M-H-Malonyl-Xyl-2Glc]-	Malonyl-vina-ginsenoside R_{16} isomer
R523	22.71	$C_{42}H_{72}O_{14}$	799.4841	-0.38	637.4297[M-H-Glc]-, 475.3792[M-H-2Glc]-	Ginsenoside Rf isomer
R524	22.72	$C_{47}H_{80}O_{17}$	915.5322	0.55	783.4983[M-H-Ara(f)]-, 621.4363[M-H-Ara(f)-Glc]-, 459.3858[M-H-Ara(f)-2Glc]-	Notoginsenoside Fe
R525	22.73	$C_{53}H_{86}O_{22}$	1073.5533	0.09	945.5457[M-H-Malonyl-Ac]-, 783.4878[M-H-Malonyl-Ac-Glc]-, 621.4355[M-H-Malonyl-Ac-2Glc]-, 459.3851[M-H-Malonyl-Ac-3Glc]-	Acetyl malonyl-ginsenoside Rd
R526	22.75	$C_{42}H_{66}O_{14}$	793.4390	2.02	631.3881[M-H-Glc]-, 569.3845[M-H-Glc-H_2O-CO_2]-, 455.3521[M-H-Glc-Glu A]-	Zingibroside R_1 isomer
R527	22.75	$C_{54}H_{85}O_{24}$	1117.5438	0.63	945.5441[M-H-2Malonyl]-,	Di-malonyl-ginseno

					783.4939[M-H-2Malonyl-Glc]$^-$, 621.4410[M-H-2Malonyl-2Glc]$^-$, 459.3829[M-H-2Malonyl-3Glc]$^-$	side Rd isomer
R528	22.76	$C_{52}H_{86}O_{19}$	1013.5674	-1.09	945.5330[M-H-(*E*)-but-2-enoyl]$^-$, 783.4898[M-H-(*E*)-but-2-enoyl-Glc]$^-$, 621.4398[M-H-(*E*)-but-2-enoyl-2Glc]$^-$, 459.3850[M-H-(*E*)-but-2-enoyl-3Glc]$^-$,	(*E*)-But-2-enoyl ginsenoside Rd
R529	22.78	$C_{57}H_{94}O_{26}$	1193.5935	-1.68	1107.5873[M-H-Malonyl]$^-$, 945.5460[M-H-Malonyl-Glc]$^-$, 783.4899[M-H-Malonyl-2Glc]$^-$, 621.4369[M-H-Malonyl-3Glc]$^-$, 459.3852[M-H-Malonyl-4Glc]$^-$	Malonyl-ginsenoside Rb$_1$ isomer
R530	22.78	$C_{50}H_{82}O_{20}$	1001.5322	0.10	915.5314[M-H-Malonyl]$^-$, 783.4910[M-H-Malonyl-Xyl]$^-$, 621.4372[M-H-Malonyl-Xyl-Glc]$^-$, 459.3824[M-H-Malonyl-Xyl-2Glc]$^-$	Malonyl-vina-ginsenoside R$_{16}$
R531	22.79	$C_{58}H_{96}O_{25}$	1191.6167 [b]	0.42	945.5385[M-H-(*E*)-but-2-enoyl-xyl]$^-$, 783.4932[M-H-(*E*)-but-2-enoyl-xyl-Glc]$^-$, 621.4421[M-H-(*E*)-but-2-enoyl-xyl-2Glc]$^-$, 459.3813[M-H-(*E*)-but-2-enoyl-xyl-3Glc]$^-$	(*E*)-but-2-enoyl-ginsenoside Rb$_3$
R532	22.79	$C_{36}H_{60}O_9$	681.4217 [b]	0.44	473.3655[M-H-Glc]$^-$	Ginsenoside Rh$_8$
R533	22.80	$C_{53}H_{88}O_{23}$	1091.5618	-1.83	945.5430[M-H-Rha]$^-$, 783.4880[M-H-Rha-Glc]$^-$, 621.4366[M-H-Rha-2Glc]$^-$, 459.3823[M-H-Rha-3Glc]$^-$	Protopanaxadiol+Rha+3Glc
R534	22.82	$C_{54}H_{92}O_{23}$	1107.5934	-1.53	945.5362[M-H-Glc]$^-$, 783.4901[M-H-2Glc]$^-$,	Ginsenoside Rb$_1$ isomer

					621.4373[M-H-3Glc]-, 459.3844[M-H-4Glc]-	
R535	22.84	$C_{50}H_{84}O_{19}$	987.5537	0.81	945.5444[M-H-Ac]-, 783.4902[M-H-Ac-Glc]-, 621.4377[M-H-Ac-2Glc]-, 459.3849[M-H-Ac-3Glc]-	Acetyl-ginsenoside Rd
R536	22.84	$C_{47}H_{80}O_{17}$	915.5327	1.09	783.4981[M-H-Xyl]-, 621.4362[M-H-Ara(f)-Glc]-, 459.3819[M-H-Ara(f)-2Glc]-	Gypenoside IX isomer/ Notoginsenoside Fe isomer
R537	22.85	$C_{62}H_{102}O_{27}$	1277.6500	-2.35	945.5450[M-H-(*E*)-but-2-enoyl-Xyl-ara(p)]-, 783.4922[M-H-(*E*)-but-2-enoyl-Xyl-ara(p)-Glc]-, 621.4388[M-H-(*E*)-but-2-enoyl-Xyl-ara(p)-2Glc]-, 459.3835[M-H-(*E*)-but-2-enoyl-Xyl-ara(p)-3Glc]-	Ginsenoside Ra_4 isomer
R538	22.85	$C_{58}H_{96}O_{24}$	1175.6233	1.70	1107.5968[M-H-(*E*)-but-2-enoyl]-, 945.5483[M-H-(*E*)-but-2-enoyl-Glc]-, 783.4868[M-H-(*E*)-but-2-enoyl-2Glc]-, 621.4406[M-H-(*E*)-but-2-enoyl-3Glc]-, 459.3838[M-H-(*E*)-but-2-enoyl-4Glc]-	Ginsenoside Ra_6 isomer
R539	22.87	$C_{42}H_{72}O_{13}$	783.4906	1.40	621.4398[M-H-Glc]-, 475.3793[M-H-Rha-Glc]-	Ginsenoside Rg_2 isomer
R540	22.91	$C_{57}H_{94}O_{23}$	1145.6131	2.01	1077.5817[M-H-(*E*)-but-2-enoyl]-, 945.5501[M-H-(*E*)-but-2-enoyl-Ara]-, 783.4906[M-H-(*E*)-but-2-enoyl-Ara-Glc]-, 621.4371[M-H-(*E*)-but-2-enoyl-Ara-2Glc]-, 459.3833[M-H-(*E*)-but-2-enoyl-Ara-3Glc]-	Ginsenoside Ra_7/Ginsenoside Ra_8/Ginsenoside Ra_9
R541	22.91	$C_{58}H_{96}O_{25}$	1191.6168 [b]	0.50	945.5497[M-H-(*E*)-but-2-enoyl-ara/xyl]-, 783.4883[M-H-(*E*)-but-2-enoyl-ara/xyl-	(*E*)-but-2-enoyl-ginsenoside Rb_2 isomer/(*E*)-but-2-en

					Glc]$^-$, 621.4357 [M-H-(*E*)-but-2-enoyl-ara/xyl-2Glc]$^-$, 459.3866[M-H-(*E*)-but-2-enoyl-ara/xyl-3Glc]$^-$	oyl-ginsenoside Rb_3 isomer/(*E*)-but-2-enoyl-ginsenoside Rc isomer
R542	22.94	$C_{52}H_{86}O_{20}$	1029.56733	-0.10	945.5441[M-H-2Ac]$^-$, 783.4896[M-H-2Ac-Glc]$^-$, 621.4382[M-H-2Ac-2Glc]$^-$, 459.3838[M-H-2Ac-3Glc]$^-$	Acetyl-pseudoginsenoside RC_1
R543	23.04	$C_{48}H_{82}O_{17}$	929.5483	0.97	767.4928[M-H-Glc]$^-$, 621.4376[M-H-Glc-Rha]$^-$, 459.3810[M-H-2Glc-Rha]$^-$	Protopanaxadiol+Rha+2Glc
R544	23.11	$C_{55}H_{92}O_{23}$	1119.5955	0.09	1077.5805[M-H-Ac]$^-$, 945.5458[M-H-Ac-Ara(p)]$^-$, 783.4879[M-H-Ac-Ara(p)-Glc]$^-$, 621.439[M-H-Ac-Ara(p)-2Glc]$^-$, 459.3823[M-H-Ac-Ara(p)-3Glc]$^-$	Ginsenoside Rs_2 isomer
R545	23.14	$C_{50}H_{84}O_{19}$	987.5541	1.22	945.5470[M-H-Ac]$^-$, 783.4825[M-H-Ac-Glc]$^-$, 621.4386[M-H-Ac-2Glc]$^-$, 459.3846[M-H-Ac-3Glc]$^-$	Acetyl-ginsenoside Rd
R546	23.16	$C_{57}H_{94}O_{26}$	1193.5955	0.00	1107.5906[M-H-Malonyl]$^-$, 945.5380[M-H-Malonyl-Glc]$^-$, 783.4918[M-H-Malonyl-2Glc]$^-$, 621.4365[M-H-Malonyl-3Glc]$^-$, 459.3841[M-H-Malonyl-4Glc]$^-$	Malonyl-ginsenoside Rb_1 isomer
R547	23.18	$C_{50}H_{82}O_{20}$	1001.5325	0.40	915.5377[M-H-Malonyl]$^-$, 783.4985[M-H-Malonyl-Xyl]$^-$, 621.4335[M-H-Malonyl-Xyl-Glc]$^-$, 459.3814[M-H-Malonyl-Xyl-2Glc]$^-$	Malonyl-vina-ginsenoside R_{16} isomer
R548	23.19	$C_{53}H_{88}O_{23}$	1091.5658	1.83	945.5432[M-H-Rha]$^-$, 783.4899[M-H-Rha-Glc]$^-$,	Protopanaxadiol+Rha+3Glc

					621.4368[M-H-Rha-2Glc]-, 459.3850[M-H-Rha-3Glc]-	
R549	23.24	$C_{52}H_{86}O_{20}$	1029.5638	0.39	987.5574[M-H-Ac]-, 945.5454[M-H-2Ac]-, 783.4882[M-H-2Ac-Glc]-, 621.4359[M-H-2Ac-2Glc]-, 459.3846[M-H-2Ac-3Glc]-	Acetyl-pseudoginsenoside RC_1
R550	23.24	$C_{53}H_{86}O_{22}$	1073.5530	-0.19	945.5417[M-H-Malonyl-Ac]-, 783.4879[M-H-Malonyl-Ac-Glc]-, 621.4317[M-H-Malonyl-Ac-2Glc]-, 459.3862[M-H-Malonyl-Ac-3Glc]-	Acetyl malonyl-ginsenoside Rd
R551	23.24	$C_{58}H_{96}O_{25}$	1191.6168 [b]	0.50	945.5501[M-H-(*E*)-but-2-enoyl-ara/xyl]-, 783.4906[M-H-(*E*)-but-2-enoyl-ara/xyl-Glc]-, 621.4371[M-H-(*E*)-but-2-enoyl-ara/xyl-2Glc]-, 459.3833[M-H-(*E*)-but-2-enoyl-ara/xyl-3Glc]-	(*E*)-but-2-enoyl-ginsenoside Rb_2 isomer/(*E*)-but-2-enoyl-ginsenoside Rb_3 isomer/(*E*)-but-2-enoyl-ginsenoside Rc isomer
R552	23.25	$C_{57}H_{94}O_{23}$	1145.6136	2.44	1077.5845[M-H-(*E*)-but-2-enoyl]-, 945.5438[M-H-(*E*)-but-2-enoyl-Ara]-, 783.4897[M-H-(*E*)-but-2-enoyl-Ara-Glc]-, 621.4393[M-H-(*E*)-but-2-enoyl-Ara-2Glc]-, 459.3865[M-H-(*E*)-but-2-enoyl-Ara-3Glc]-	Ginsenoside Ra_7 ismoer/Ginsenoside Ra_8 isomer/Ginsenoside Ra_9 isomer
R553	23.25	$C_{51}H_{84}O_{19}$	999.5533	0.40	915.5260[M-H-2Ac]-, 783.4859[M-H-2Ac-Xyl]-, 621.4383[M-H-2Ac-Xyl-Glc]-, 459.3867[M-H-2Ac-Xyl-2Glc]-	Diacetyl-vina-ginsenoside R_{16}
R554	23.26	$C_{50}H_{82}O_{20}$	1001.5324	0.30	915.5379[M-H-Malonyl]-,	Malonyl-vina-ginse

					783.4991[M-H-Malonyl-Xyl]-, 621.4357[M-H-Malonyl-Xyl-Glc]-, 459.3828[M-H-Malonyl-Xyl-2Glc]-	noside R_{16} isomer
R555	23.26	$C_{54}H_{90}O_{24}$	1121.5757	1.16	945.5435[M-H-Glu A]-, 783.4855[M-H-Glu A-Glc]-, 621.4413[M-H-Glu A-2Glc]-, 459.3825[M-H-Glu A-3Glc]-	Protopanaxadiol+Glu A+3Glc
R556	23.36	$C_{42}H_{70}O_{12}$	765.4795	0.78	619.4229[M-H-Rha]-, 457.3707[M-H-Rha-Glc]-	Ginsenoside Rg_6 [a]
R557	23.38	$C_{42}H_{66}O_{14}$	793.4376	0.25	631.3796[M-H-Glc]-, 613.3732[M-H-Glc-H_2O]-, 569.3779[M-H-Glc-H_2O-CO_2]-, 455.3522[M-H-Glc-Glu A]-	Zingibroside R_1 isomer
R558	23.39	$C_{52}H_{86}O_{19}$	1013.5671	-1.38	783.4913[M-H-(*E*)-but-2-enoyl-Glc]-, 459.3881[M-H-(*E*)-but-2-enoyl-3Glc]-,	(*E*)-But-2-enoyl ginsenoside Rd
R559	23.44	$C_{53}H_{90}O_{22}$	1077.5827	-1.67	945.5430[M-H-Xyl/Ara]-, 783.4885[M-H-Xyl/Ara-Glc]-, 621.4370[M-H-Xyl/Ara-2Glc]-, 459.3825[M-H-Xyl/Ara-3Glc]-	Ginsenoside Rc isomer/Ginsenoside Rb_2 isomer/Ginsenoside Rb_3 isomer
R560	23.52	$C_{42}H_{66}O_{14}$	793.4357	-2.14	631.3824[M-H-Glc]-, 569.3779[M-H-Glc-H_2O-CO_2]-, 455.3513[M-H-Glc-Glu A]-	Zingibroside R_1 isomer
R561	23.56	$C_{48}H_{82}O_{17}$	929.5489	1.61	783.4911[M-H-Rha]-, 621.4376[M-H-Rha-Glc]-, 459.3846[M-H-Rha-2Glc]-	Protopanaxadiol+Rha+2Glc
R562	23.57	$C_{42}H_{72}O_{14}$	799.4846	0.25	637.4304[M-H-Glc]-, 475.3803[M-H-2Glc]-	Ginsenoside Rf isomer
R563	23.58	$C_{58}H_{96}O_{25}$	1191.6167 [b]	0.42	945.5428[M-H-(*E*)-but-2-enoyl-ara/xyl]-	(*E*)-but-2-enoyl-gins

					, 783.4925[M-H-(*E*)-but-2-enoyl-ara/xyl-Glc]-, 621.4429[M-H-(*E*)-but-2-enoyl-ara/xyl-2Glc]-, 459.3865[M-H-(*E*)-but-2-enoyl-ara/xyl-3Glc]-	enoside Rb_2 isomer/(*E*)-but-2-enoyl-ginsenoside Rb_3 isomer/(*E*)-but-2-enoyl-ginsenoside Rc isomer
R564	23.60	$C_{57}H_{94}O_{23}$	1145.6085	-2.01	1077.5881[M-H-(*E*)-but-2-enoyl]-, 945.5385[M-H-(*E*)-but-2-enoyl-Ara]-, 783.4932[M-H-(*E*)-but-2-enoyl-Ara-Glc]-, 621.4421[M-H-(*E*)-but-2-enoyl-Ara-2Glc]-, 459.3815[M-H-(*E*)-but-2-enoyl-Ara-3Glc]-	Ginsenoside Ra_7 ismoer/Ginsenoside Ra_8 isomer/Ginsenoside Ra_9 isomer
R565	23.61	$C_{50}H_{84}O_{18}$	971.5586	0.72	929.5477[M-H-Ac]-, 783.4919[M-H-Ac-Rha]-, 621.4371[M-H-Ac-Rha-Glc]-, 459.3843[M-H-Ac-Rha-2Glc]-	Protopanaxadiol+ Rha+Glc+Acetyl Glc
R566	23.64	$C_{42}H_{70}O_{12}$	765.4793	0.52	619.4216[M-H-Rha]-, 457.3697[M-H-Rha-Glc]-	Ginsenoside F_4 [a]
R567	23.64	$C_{52}H_{86}O_{19}$	1013.5687	0.20	945.5403[M-H-(*E*)-but-2-enoyl]-, 783.4888[M-H-(*E*)-but-2-enoyl-Glc]-, 621.4343[M-H-(*E*)-but-2-enoyl-2Glc]-, 459.3857[M-H-(*E*)-but-2-enoyl-3Glc]-,	(*E*)-But-2-enoyl ginsenoside Rd
R568	23.66	$C_{50}H_{84}O_{18}$	971.5580	0.10	929.5450[M-H-Ac]-, 621.4362[M-H-Ac-Rha-Glc]-, 459.3839[M-H-Ac-Rha-2Glc]-	Protopanaxadiol+ Rha+Glc+Acetyl Glc
R569	23.66	$C_{51}H_{84}O_{19}$	999.5548	1.90	915.5387[M-H-2Ac]-, 783.4864[M-H-2Ac-Xyl]-, 621.4387[M-H-2Ac-Xyl-Glc]-,	Diacetyl-vina-ginsenoside R_{16}

					459.3829[M-H-2Ac-Xyl-2Glc]$^-$	
R570	23.67	$C_{62}H_{102}O_{27}$	1277.6528	-0.16	1077.5958[M-H-(*E*)-but-2-enoyl-Xyl]$^-$, 945.5408[M-H-(*E*)-but-2-enoyl-Xyl-ara(p)]$^-$, 783.4843[M-H-(*E*)-but-2-enoyl-Xyl-ara(p)-Glc]$^-$, 621.4289[M-H-(*E*)-but-2-enoyl-Xyl-ara(p)-2Glc]$^-$, 459.3833[M-H-(*E*)-but-2-enoyl-Xyl-ara(p)-3Glc]$^-$	Ginsenoside Ra_4 isomer
R571	23.73	$C_{48}H_{82}O_{18}$	945.5441	1.90	783.4854[M-H-Glc]$^-$, 621.4340[M-H-2Glc]$^-$, 459.3810[M-H-3Glc]$^-$	Ginsenoside Rd isomer
R572	23.78	$C_{53}H_{90}O_{22}$	1077.5823	-2.04	945.5441[M-H-Xyl/Ara]$^-$, 783.4904[M-H-Xyl/Ara-Glc]$^-$, 621.4366[M-H-Xyl/Ara-2Glc]$^-$, 459.3875[M-H-Xyl/Ara-3Glc]$^-$	Ginsenoside Rc isomer/Ginsenoside Rb_2 isomer/Ginsenoside Rb_3 isomer
R573	23.86	$C_{49}H_{82}O_{18}$	957.5411	-1.25	915.5229[M-H-Ac]$^-$, 783.5002[M-H-Ac-Xyl]$^-$, 621.4393[M-H-Ac-Xyl-Glc]$^-$, 459.3856[M-H-Ac-Xyl-2Glc]$^-$	Acetyl-vina-ginsenoside R_{16}
R574	23.89	$C_{50}H_{84}O_{18}$	971.5565	-1.44	929.5476[M-H-Ac]$^-$, 783.4915[M-H-Ac-Rha]$^-$, 621.4382[M-H-Ac-Rha-Glc]$^-$, 459.3851[M-H-Ac-Rha-2Glc]$^-$	Protopanaxadiol+ Rha+Glc+Acetyl Glc
R575	23.92	$C_{44}H_{74}O_{14}$	825.5002	0.24	783.4849[M-H-Ac]$^-$, 621.4310[M-H-Ac-Glc]$^-$, 459.3870[M-H-Ac-2Glc]$^-$	Acetyl-ginsenoside Rg_3
R576	23.93	$C_{49}H_{82}O_{18}$	957.5415	-0.84	915.5327[M-H-Ac]$^-$, 783.4895[M-H-Ac-Xyl]$^-$,	Acetyl-vina-ginsenoside R_{16}

					621.4369[M-H-Ac-Xyl-Glc]-, 459.3841[M-H-Ac-Xyl-2Glc]-	
R577	23.94	$C_{54}H_{88}O_{20}$	1055.5774	-1.61	945.5427[M-H-(*E*)-but-2-enoyl-Ac]-, 783.4982[M-H-(*E*)-but-2-enoyl-Ac-Glc]-, 621.4403[M-H-(*E*)-but-2-enoyl-Ac-2Glc]-, 459.3823[M-H-(*E*)-but-2-enoyl-Ac-3Glc]-	(*E*)-But-2-enoyl-pseudoginsenoside RC_1
R578	23.99	$C_{48}H_{82}O_{18}$	945.5440	1.80	783.4922[M-H-Glc]-, 621.4371[M-H-2Glc]-, 459.3831[M-H-3Glc]-	Ginsenoside Rd isomer
R579	24.01	$C_{48}H_{82}O_{17}$	975.5515 [b]	-1.44	783.4911[M-H-Rha]-, 621.4363[M-H-Rha-Glc]-, 459.3846[M-H-Rha-2Glc]-	Protopanaxadiol+Rha+2Glc
R580	24.03	$C_{42}H_{66}O_{14}$	793.4370	-0.50	631.3844[M-H-Glc]-, 613.3732[M-H-Glc-H_2O]-, 569.3886[M-H-Glc-H_2O-CO_2]-, 455.3533[M-H-Glc-Glu A]-	Zingibroside R_1
R581	24.11	$C_{48}H_{82}O_{18}$	945.5443	2.12	783.4846[M-H-Glc]-, 621.4323[M-H-2Glc]-, 475.3751[M-H-2Glc-Rha]-	Protopanaxatriol+2Glc+Rha
R582	24.12	$C_{47}H_{74}O_{18}$	925.4802	0.54	793.4401[M-H-Xyl/Ara]-, 763.4348[M-H-Glc]-, 613.3751[M-H-Xyl/Ara-Glc-H_2O]-, 455.3525[M-H-Xyl/Ara-Glc-Glu A]-	Pseudo-ginsenoside-RT_1 isomer/Chikusetsusaponin IV isomer
R583	24.18	$C_{53}H_{90}O_{22}$	1077.5853	0.74	945.5417[M-H-Xyl/Ara]-, 783.4888[M-H-Xyl/Ara-Glc]-, 621.4379[M-H-Xyl/Ara-2Glc]-, 459.3851[M-H-Xyl/Ara-3Glc]-	Ginsenoside Rc isomer/Ginsenoside Rb_2 isomer/Ginsenoside Rb_3 isomer isomer

R584	24.19	$C_{48}H_{82}O_{17}$	975.5514 [b]	-1.54	783.4980[M-H-Rha]$^-$, 621.4443[M-H-Rha-Glc]$^-$, 459.3860[M-H-Rha-2Glc]$^-$	Protopanaxadiol+ Rha+2Glc
R585	24.28	$C_{44}H_{74}O_{14}$	825.5013	1.57	783.4891[M-H-Ac]$^-$, 621.4454[M-H-Ac-Glc]$^-$, 459.3877[M-H-Ac-2Glc]$^-$	Acetyl-ginsenoside Rg_3
R586	24.32	$C_{52}H_{86}O_{19}$	1013.5699	1.38	945.5543[M-H-(*E*)-but-2-enoyl]$^-$, 783.4907[M-H-(*E*)-but-2-enoyl-Glc]$^-$, 621.4354[M-H-(*E*)-but-2-enoyl-2Glc]$^-$, 459.3846[M-H-(*E*)-but-2-enoyl-3Glc]$^-$,	(*E*)-But-2-enoyl ginsenoside Rd
R587	24.33	$C_{44}H_{68}O_{15}$	835.4499	2.27	793.4344[M-H-Ac]$^-$, 631.3805[M-H-Ac-Glc]$^-$, 613.3746[M-H-Ac-Glc-H_2O]$^-$, 455.3546[M-H-Ac-Glc-Glu A]$^-$	Acetyl-chikusetsusa ponin Iva
R588	24.49	$C_{42}H_{66}O_{14}$	793.4382	1.01	631.3908[M-H-Glc]$^-$, 613.3768[M-H-Glc-H_2O]$^-$, 455.3523[M-H-Glc-Glu A]$^-$	Zingibroside R_1 isomer
R589	24.57	$C_{44}H_{74}O_{14}$	825.5001	0.12	783.4015[M-H-Ac]$^-$, 621.3644[M-H-Ac-Glc]$^-$, 459.3320[M-H-Ac-2Glc]$^-$	Acetyl-ginsenoside Rg_3
R590	24.61	$C_{50}H_{84}O_{18}$	1017.5620 [b]	-1.38	929.5466[M-H-Ac]$^-$, 783.4886[M-H-Ac-Rha]$^-$, 621.4365[M-H-Ac-Rha-Glc]$^-$, 459.3849[M-H-Ac-Rha-2Glc]$^-$	Protopanaxadiol+ Rha+Glc+Acetyl Glc
R591	24.65	$C_{42}H_{70}O_{13}$	827.4807 [b]	1.69	619.4224[M-H-Glc]$^-$, 457.3675[M-H-2Glc]$^-$	Dehydrated-protop anaxatriol+2Glc
R592	24.66	$C_{49}H_{82}O_{18}$	1003.5471 [b]	-0.73	915.5284[M-H-Ac]$^-$, 783.4954[M-H-Ac-Xyl]$^-$, 621.4387[M-H-Ac-Xyl-Glc]$^-$,	Acetyl-vina-ginseno side R_{16}

					459.3891[M-H-Ac-Xyl-2Glc]-	
R593	24.68	$C_{42}H_{72}O_{13}$	783.4900	0.64	621.4363[M-H-Glc]-, 459.3862[M-H-2Glc]-	Ginsenoside 20(*S*)-Rg_3 [a]
R594	24.72	$C_{44}H_{74}O_{14}$	825.4999	-0.12	783.4898[M-H-Ac]-, 621.4366[M-H-Ac-Glc]-, 459.3855[M-H-Ac-2Glc]-	Acetyl-ginsenoside Rg_3
R595	24.73	$C_{50}H_{84}O_{18}$	971.5569	-1.03	783.4908[M-H-Ac-Rha]-, 621.4406[M-H-Ac-Rha-Glc]-, 459.3844[M-H-Ac-Rha-2Glc]-	Protopanaxadiol+ Rha+Glc+Acetyl Glc
R596	24.78	$C_{57}H_{94}O_{23}$	1145.6096	-1.05	1077.5804[M-H-(*E*)-but-2-enoyl]-, 945.5497[M-H-(*E*)-but-2-enoyl-Ara]-, 783.4883[M-H-(*E*)-but-2-enoyl-Ara-Glc]-, 621.4357[M-H-(*E*)-but-2-enoyl-Ara-2Gl c]-, 459.3866[M-H-(*E*)-but-2-enoyl-Ara-3Gl c]-	Ginsenoside Ra_7 ismoer/Ginsenoside Ra_8 isomer/Ginsenoside Ra_9 isomer
R597	24.78	$C_{53}H_{90}O_{22}$	1077.5824	-1.95	945.5389[M-H-Xyl/Ara]-, 783.4782[M-H-Xyl/Ara-Glc]-, 621.4380[M-H-Xyl/Ara-2Glc]-, 459.3843[M-H-Xyl/Ara-3Glc]-	Ginsenoside Rc isomer/Ginsenoside Rb_2 isomer/Ginsenoside Rb_3 isomer
R598	24.80	$C_{48}H_{82}O_{17}$	929.5462	-1.29	783.4874[M-H-Rha]-, 621.4382[M-H-Rha-Glc]-, 459.3836[M-H-Rha-2Glc]-	Protopanaxadiol+ Rha+2Glc
R599	24.87	$C_{42}H_{72}O_{13}$	783.4905	1.28	621.4341[M-H-Glc]-, 459.3784[M-H-2Glc]-	Ginsenoside 20(*R*)-Rg_3 [a]
R600	24.90	$C_{56}H_{94}O_{24}$	1149.6052	-0.43	1107.5938[M-H-Ac]-, 945.5446[M-H-Ac-Glc]-, 783.4897 [M-H-Ac-2Glc]-, 621.4352[M-H-Ac-3Glc]-, 459.3840[M-H-Ac-4Glc]-	Quinquenoside R_1 isomer
R601	24.90	$C_{44}H_{74}O_{14}$	825.5005	0.61	783.4850[M-H-Ac]-,	Acetyl-ginsenoside

					621.4359[M-H-Ac-Glc]$^-$, 459.3834[M-H-Ac-2Glc]$^-$	Rg_3
R602	24.92	$C_{42}H_{70}O_{13}$	827.4801 [b]	0.97	619.4156[M-H-Glc]$^-$, 457.3686[M-H-2Glc]$^-$	Dehydrated-protopanaxatriol+2Glc
R603	25.00	$C_{42}H_{66}O_{14}$	793.4385	1.39	631.3843[M-H-Glc]$^-$, 613.3739[M-H-Glc-H_2O]$^-$, 569.3808[M-H-Glc-H_2O-CO_2]$^-$, 455.3543[M-H-Glc-Glu A]$^-$	Zingibroside R_1 isomer
R604	25.09	$C_{44}H_{74}O_{14}$	825.5007	0.85	783.4950[M-H-Ac]$^-$, 621.4361[M-H-Ac-Glc]$^-$, 459.3793[M-H-Ac-2Glc]$^-$	Acetyl-ginsenoside Rg_3
R605	25.13	$C_{51}H_{84}O_{21}$	1031.5428	0.10	945.5349[M-H-Malonyl]$^-$, 783.4881[M-H-Malonyl-Glc]$^-$, 621.4369[M-H-Malonyl-2Glc]$^-$, 459.3839[M-H-Malonyl-3Glc]$^-$	Malonyl-ginsenoside Rd isomer
R606	25.21	$C_{44}H_{74}O_{14}$	825.5006	0.73	783.4951[M-H-Ac]$^-$, 621.4360[M-H-Ac-Glc]$^-$, 459.3799[M-H-Ac-2Glc]$^-$	Acetyl-ginsenoside Rg_3
R607	25.48	$C_{41}H_{70}O_{12}$	753.4787	-0.27	621.4398[M-H-Ara]$^-$ 459.3819[M-H-Ara-Glc]$^-$	Ginsenoside MC
R608	25.77	$C_{44}H_{74}O_{14}$	825.5009	1.09	783.4903[M-H-Ac]$^-$, 621.4368[M-H-Ac-Glc]$^-$, 459.3835[M-H-Ac-2Glc]$^-$	Acetyl-ginsenoside Rg_3
R609	25.87	$C_{65}H_{100}O_{21}$	1215.6666	-1.07	955.4909[M-H-Polyacetylene]$^-$, 793.4325[M-H-Polyacetylene-Glc]$^-$, 731.4387[M-H-Polyacetylene-Glc-CO_2-H_2O]$^-$, 613.3630[M-H-Polyacetylene-2Glc-H_2O]$^-$, 455.3521[M-H-Polyacetylene-2Glc-Glu A]$^-$	Polyacetyleneginsenoside-Ro isomer

R610	25.95	$C_{42}H_{70}O_{13}$	827.4803 [b]	1.21	619.4288[M-H-Glc]$^-$, 457.3708[M-H-2Glc]$^-$	Dehydrated-protopanaxatriol+2Glc
R611	26.04	$C_{65}H_{100}O_{21}$	1215.6681	0.16	955.4936[M-H-Polyacetylene]$^-$, 793.4349[M-H-Polyacetylene-Glc]$^-$, 731.4348[M-H-Polyacetylene-Glc--CO_2-H_2O]$^-$, 613.3741[M-H-Polyacetylene-2Glc-H_2O]$^-$, 455.3537[M-H-Polyacetylene-2Glc-GluA]$^-$	Polyacetyleneginsenoside-Ro isomer
R612	26.19	$C_{65}H_{100}O_{21}$	1215.6685	0.49	955.4891[M-H-Polyacetylene]$^-$, 793.4378[M-H-Polyacetylene-Glc]$^-$, 731.4383[M-H-Polyacetylene-Glc--CO_2-H_2O]$^-$, 613.3792[M-H-Polyacetylene-2Glc-H_2O]$^-$, 455.3537[M-H-Polyacetylene-2Glc-GluA]$^-$	Polyacetyleneginsenoside-Ro
R613	26.23	$C_{50}H_{84}O_{19}$	987.5533	0.41	621.4418[M-H-Ac-2Glc]$^-$, 459.3831[M-H-Ac-3Glc]$^-$	Acetyl-ginsenoside Rd
R614	26.36	$C_{65}H_{100}O_{21}$	1215.6683	0.33	955.4894[M-H-Polyacetylene]$^-$, 793.4391[M-H-Polyacetylene-Glc]$^-$, 455.3493[M-H-Polyacetylene-2Glc-GluA]$^-$	Polyacetyleneginsenoside-Ro isomer
R615	26.67	$C_{36}H_{62}O_8$	621.4360	-0.97	459.3872[M-H-Glc]$^-$	Ginsenoside Compound K
R616	26.72	$C_{42}H_{70}O_{12}$	765.4795	0.78	603.4263[M-H-Glc]$^-$, 441.3346[M-H-2Glc]$^-$	Ginsenoside Rk1
R617	26.85	$C_{50}H_{84}O_{19}$	987.5538	0.91	945.5393[M-H-Ac]$^-$, 783.4979[M-H-Ac-Glc]$^-$, 621.4387[M-H-Ac-2Glc]$^-$, 459.3828[M-H-Ac-3Glc]$^-$	Acetyl-ginsenoside Rd

R618	26.90	$C_{42}H_{70}O_{12}$	765.4798	1.18	603.4271[M-H-Glc]$^-$, 441.3354[M-H-2Glc]$^-$	Ginsenoside Rg5
R619	27.14	$C_{36}H_{62}O_8$	621.4361	-0.80	459.3809[M-H-Glc]$^-$	20(*S*)-Ginsenoside Rh2 [a]
R620	27.27	$C_{36}H_{62}O_8$	621.4365	-0.16	459.3870[M-H-Glc]$^-$	20(*R*)-Ginsenoside Rh2 [a]

a: Compound identified by comparison with the standard reference; *b*: [M-H+HAc]$^-$ ion.
